# Lattice Structures in Additive Manufacturing for Biomedical Applications: A Systematic Review

**DOI:** 10.3390/polym17172285

**Published:** 2025-08-23

**Authors:** Samuel Polo, Amabel García-Domínguez, Eva María Rubio, Juan Claver

**Affiliations:** Department of Manufacturing Engineering, Industrial Engineering School, National University of Distance Education (UNED), St/Juan Del Rosal 12, E28040 Madrid, Spain; spolo64@alumno.uned.es (S.P.); agarcia@ind.uned.es (A.G.-D.); jclaver@ind.uned.es (J.C.)

**Keywords:** additive manufacturing, 3D printing, lattice structures, architected cellular materials, biomaterials, biomedical applications, systematic review

## Abstract

The present study offers a systematic review of the current state of research on lattice structures manufactured by additive technologies for biomedical applications, with the aim of identifying common patterns, such as the use of triply periodic minimal surfaces (TPMS) for bone scaffolds, as well as technological gaps and future research opportunities. Employing the PRISMA (Preferred Reporting Items for Systematic Reviews and Meta-Analyses) methodology, the review process ensures methodological rigor and replicability across the identification, screening, eligibility, and inclusion phases. Additionally, PRISMA was tailored by prioritizing technical databases and engineering-specific inclusion criteria, thereby aligning the methodology with the scope of this field. In recent years, a substantial surge in interdisciplinary research has underscored the promise of architected porous structures in enhancing mechanical compatibility, fostering osseointegration, and facilitating personalized medicine. A growing body of literature has emerged that explores the optimization of geometric features to replicate the behavior of biological tissues, particularly bone. Additive manufacturing (AM) has played a pivotal role in enabling the fabrication of complex geometries that are otherwise unachievable by conventional methods. The applications of lattice structures range from permanent load-bearing implants, commonly manufactured through selective laser melting (SLM), to temporary scaffolds for tissue regeneration, often produced with extrusion-based processes such as fused filament fabrication (FFF) or direct ink writing (DIW). Notwithstanding these advances, challenges persist in areas such as long-term in vivo validation, standardization of mechanical and biological testing, such as ISO standards for fatigue testing, and integration into clinical workflows.

## 1. Introduction

In the current context of Industry 4.0, engineering and industrial applications are undergoing a transition towards greater sustainability. This transition involves the design and manufacture of products that require less material, simpler manufacturing processes, and lower energy consumption, without compromising product functionality. Throughout history, humans have consistently turned to nature as a source of inspiration to solve complex design and functionality problems, enabling evolutionarily optimized solutions to be transferred to the field of engineering. In this context, cellular structures—found in bones, corals, and plant tissues—are establishing themselves as a benchmark model by offering an exceptional combination of lightness, mechanical strength, and geometric customization [1,2].

These structures are of particular importance because they represent one of the most effective methodologies for applying topological optimization, which is a design technique that allows material to be redistributed intelligently within certain boundary conditions. The primary objective of this method is to minimize the mass or volume of a component without compromising its functional performance in aspects such as rigidity, strength, or energy absorption capacity. Two primary methodologies can be employed for the removal or distribution of material through topological optimization: generative design and cellular structures. Generative design is a critical component of this process, leveraging advanced computational algorithms to explore a multitude of structural configurations. These algorithms are employed to identify regions of low mechanical demand, thereby enabling the removal of material. Cellular structures contribute to this optimization by redistributing material internally in the form of periodic, gradual, or hierarchical patterns that reproduce highly efficient geometric configurations from a structural point of view [3].

The notion of cellular structure was initially put forward by Gibson and Ashby in the 1980s, drawing upon the analysis of materials exhibiting a porous internal morphology, characterized by a network of cells dispersed throughout the material’s volume [4]. These structures can be categorized into two broad classifications: foams and lattices. Foams are distinguished by a stochastic distribution of cells, which can be either open or closed. These cells are characterized by their high porosity and mechanical behavior, which is dependent on the density and random connectivity of their walls. Conversely, lattice structures are a three-dimensional typology predicated on the periodic repetition of geometrically defined unit cells [5].

Lattice structures are therefore a particularly promising solution due to their controlled and repetitive architecture. A salient feature of these models is their capacity for customization through the utilization of computational modeling tools. This capability enables the manipulation of parameters such as relative density, stress direction, and mass distribution. This enables them to meet the demanding mechanical and functional properties required in today’s most competitive sectors [4]. Furthermore, their architectural design enables the optimization of the strength-to-weight ratio, as well as the incorporation of additional functionalities, such as thermal dissipation, impact absorption, and controlled permeability [6].

For years, the fabrication of cellular structures has been carried out through conventional methodologies, encompassing techniques such as molding, casting, laser cutting of overlapping sheets, profile assembly, and subtractive machining [7]. In essence, these methodologies provided functional solutions; however, they necessitated the compromise of design for technical feasibility. The resulting geometries were, in many cases, repetitive, orthogonal, and topologically simple. Moreover, any effort to modify critical properties such as porosity, element thickness, or internal connectivity resulted in an exponential escalation in process complexity or rendered it directly unfeasible. The result was a discrepancy between the theoretical potential of cellular structures and the actual products that could be produced. Nevertheless, this discrepancy did not impede the progression of scientific research. Throughout the 20th century, particularly in its final decades, there were concerted efforts to enhance the structural and functional performance of these materials. These efforts resulted in the development of optimized solutions, including sintered metal foams, aluminum honeycomb structures, and hybrid combinations of materials with specific functions, such as impact absorption, thermal insulation, or weight reduction. However, these advancements shared a common limitation: they were subject to processes with minimal control over the internal architecture. The advent of additive manufacturing (AM) technologies has profoundly transformed this paradigm [1,7].

AM technologies have effectively eliminated numerous restrictions imposed by conventional manufacturing methods. In contrast to the latter, where geometry was constrained by the capabilities of the available tools, AM inverts the logic, whereby the process adapts to the design. AM, also referred to as “3D printing,” is a term used to describe a set of technologies that construct three-dimensional objects by successively adding layers of material, beginning directly from a digital model. This method, which involves a layering process, eliminates the necessity for conventional tooling, molds, or intricate machining processes. Consequently, it enables the fabrication of components with highly intricate internal geometries, concealed channels, or meticulously detailed cellular structures [4].

Consequently, AM has emerged as the prevailing method for the fabrication of geometrically intricate materials, enabling the integration of internal topological variations without incurring additional costs or complexity. This facilitates the production of components with mechanically adapted properties tailored to their spatial environment. This level of control is particularly relevant for lattice structures, as key parameters such as elastic modulus, compressive strength, energy absorption, and permeability can be controlled by adjusting the geometry of the unit cell and its distribution in the overall volume [8,9]. The integration of lattice structures and AM technologies has exhibited considerable promise across a diverse array of industrial sectors. Within this extensive range of applications, the biomedical sector is particularly noteworthy for its significant advancements in this field, as it is an environment that demands highly specialized structural solutions to meet the intricate demands of functionality, anatomy, and biology [10].

Despite the recent surge in publications concerning the use of additive technologies for manufacturing lattice structures in the biomedical sector, several recent studies concur that there is a paucity of systematic, contemporary compilations that consolidate existing knowledge in this field. Moreover, a significant proportion of extant literature addresses particular case studies or emphasizes specific applications, omitting an overview of the structural types utilized, the manufacturing processes employed, or the design criteria adopted. The absence of a cohesive and interdisciplinary viewpoint on the extant knowledge severely restricts the capacity to delineate unambiguous progress, identify synergies across disciplines, or establish shared design and validation criteria. This predicament is particularly pronounced in a domain where interdisciplinarity is paramount and collaboration between engineering, medicine, and materials science is indispensable.

### Research Objective and Contribution

The objective of this review is to address the gap identified in the scientific literature on lattice structures manufactured using additive technologies applied to the biomedical sector, in a critical and systematic manner. In this manner, the AM and lattice structures most widely used or with the greatest potential in the biomedical sector will be explored. The most relevant information to date will be organized and analyzed, allowing not only to clarify the current state of knowledge, but also to detect common patterns and scientific gaps and propose new lines of research. This document is also intended to serve as a useful reference guide for researchers, engineers, and professionals in the biomedical field, providing a structured basis to facilitate the practical application of these technologies and their progressive integration into the design and manufacture of advanced biomedical solutions.

From this initial basis, the procedure for conducting the literature search was defined. The researchers elected to utilize a systematic review methodology based on the PRISMA (Preferred Reporting Items for Systematic Reviews and Meta-Analyses) guidelines, adapted to the engineering context. This choice enables the process of selecting, analyzing, and classifying information to be structured in a transparent and replicable manner. Finally, once the relevant information has been extracted, the results are presented in a summarized and organized manner, facilitating consultation of key contributions for researchers, professionals, and engineers interested in the design and application of lattice structures in the biomedical field. This facilitates the establishment of possible future lines of research and final conclusions on the state of the art.

## 2. Materials and Methods

As previously delineated, the PRISMA methodology, adapted to the domain of engineering by prioritizing technical databases and engineering-specific inclusion criteria, will be employed in this state-of-the-art study. This choice is justified by PRISMA’s ability to bring transparency, comprehensiveness, and traceability to the review process. This allows for the systematic identification, selection, and evaluation of the most important literature in the fields searched. Furthermore, PRISMA was initially developed within the domain of health sciences, and its application in engineering studies is becoming increasingly prevalent. This development offers a clear and replicable methodological structure suitable for the critical integration of results in interdisciplinary fields, such as the one addressed in this work. However, it is imperative to acknowledge the inherent limitations of PRISMA. One such limitation pertains to the dependence on the availability and quality of the studies included, which may be contingent upon the scope of the databases consulted or the precision of the terms employed in the search strategy. Furthermore, PRISMA does not provide specific criteria for assessing the methodological validity or relevance of the selected studies, delegating this responsibility to the researcher’s judgment. This may introduce bias if not managed rigorously.

**Figure 1:**
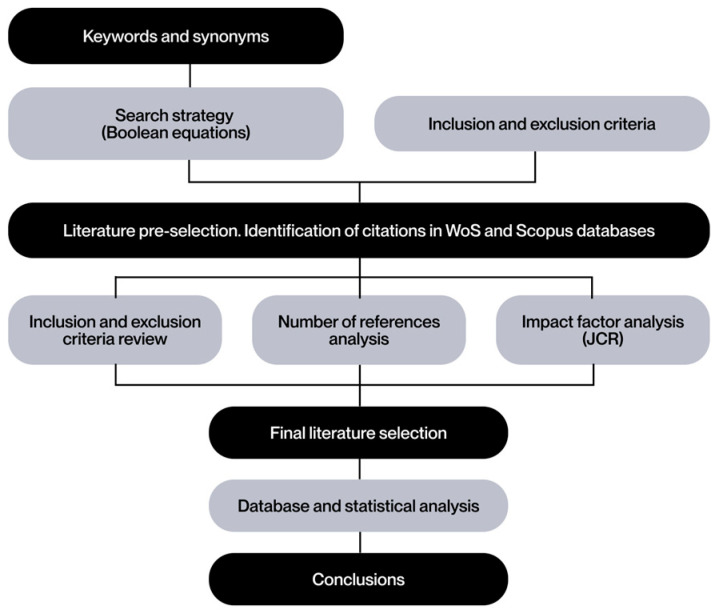
Literature search strategy based on Blanco et al. [11].

To orient the PRISMA methodology toward the field of engineering, the document published by Blanco et al., 2021 [11] is taken as a reference. This strategy is shown in Figure 1 as a guide for searching and selecting the literature on which the review will be based.

### 2.1. Inclusion and Exclusion Criteria Used in the Search

The preliminary definition of inclusion and exclusion criteria provides a solid foundation for research and ensures that results align with the stated objectives. These criteria not only delineate the scope of the study but also serve to minimize potential biases and ensure that the selected literature is relevant and representative for further analysis.

As previously indicated, the present analysis focuses on recently published studies. Consequently, the publication period under consideration is limited to the last five years, thereby excluding recent trends in the utilization of additively manufactured lattice structures within the biomedical sector. The selection of studies has been primarily oriented towards journal articles, given their recognition for using rigorous methodologies and presenting reliable results. In addition, the review articles provide a comprehensive overview of the current state of the field, emphasizing the major advancements. The relevance of conference proceedings for presenting the latest progress, innovations, and emerging trends has also been considered. These sources are of particular importance in identifying new approaches in the areas under investigation.

**Figure 2:**
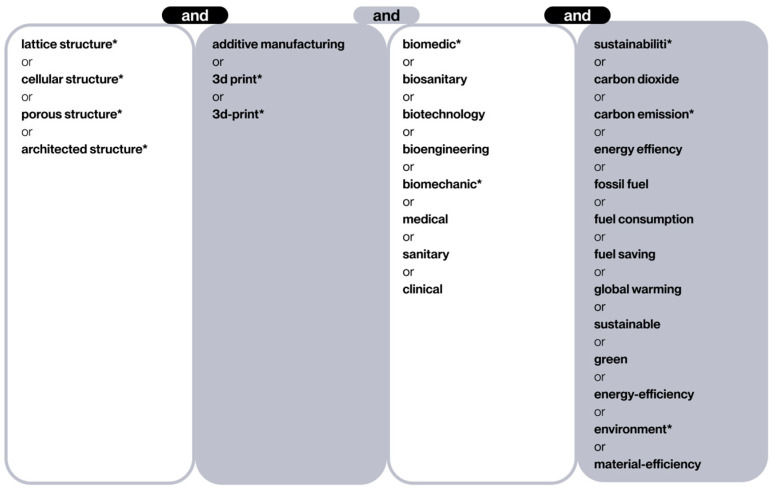
Terms and synonyms entered in this search. The asterisk (*) serves as an operator that captures any letters that follow it.

To refine the accuracy of the search within the defined topic, a set of keywords and synonyms has been defined in the form of Boolean equations (see Figure 2). This methodological approach ensures a high level of coverage and accuracy, thereby minimizing the likelihood of omitting relevant studies.

The databases selected for this review were Web of Science (WoS) and Scopus. This is attributable to their extensive collection of high-quality, peer-reviewed publications and their advanced search and analysis tools. The WoS database offers access to studies published in leading journals, thereby enhancing the credibility of the review. Scopus is another valuable source due to its multidisciplinary coverage, including a vast number of scientific articles, conference proceedings, and book chapters. The advanced filtering and Boolean search capabilities of both databases facilitate comprehensive and precise bibliographic searches, while their bibliometric tools enable trend analysis and citation tracking. The selection of WoS and Scopus is consistent with PRISMA’s fundamental principles of rigor, transparency, and methodological traceability, thereby ensuring the validity of the review and the inclusion of related studies.

The selection of publications has been limited to those that are open access, with the aim of ensuring their global accessibility and thus promoting collaboration in research. The application of strict quality criteria has been another essential element of the review process. The inclusion of articles has been limited to those peer-reviewed and published in journals classified in the first quartiles (Q1 and Q2), according to the Journal Impact Factor and the Journal Citation Reports. This guarantees a high level of reliability and rigor in the texts analyzed. These criteria are summarized in Table 1.

### 2.2. Definition of Search Criteria

The objective of this literature review is to address the main problem previously identified regarding the current situation of additively manufactured lattice structures in the biomedical sector. In accordance with the aforementioned search methodology, a Boolean search strategy was employed, utilizing keywords and their synonyms in structured equations.

The terminology employed during the search process is delineated in Figure 2. The initial column delineates lattice structures, which are a component of cellular structures. The second column restricts the search to the domain of AM technologies, colloquially referred to as “3D printing”. The third column filters the biomedical sector, including different terms in order to cover a broad spectrum of sub-areas in this field, from biomedical engineering to direct clinical applications. Finally, the final column incorporates terms related to sustainability and energy efficiency, with the objective of maintaining a focus on minimizing environmental impact. Given the broad scope of the topic and the anticipated abundance of irrelevant articles, the advanced “exact search” function of WoS was employed to guarantee that each selected document contained at least one term from each column. This function is performed automatically in Scopus searches.

The Boolean equations employed in the search are as follows:

WoS: TS = (lattice structure* OR cellular structure* OR porous structure* OR architected structure*) AND TS = (additive manufacturing OR 3d-print* OR 3D print*) AND TS = (biomedic* OR biosanitary OR biotechnology OR bioengineering OR biomechanic* OR medical OR sanitary OR clinical) AND TS = (sustainabili* OR carbon dioxide OR carbon emission* OR energy efficiency OR fossil fuel OR fuel consumption OR fuel saving OR global warming OR sustainable OR green OR energy-efficiency OR environment* OR material-efficiency)

Scopus: TITLE-ABS-KEY (“lattice structure*” OR “cellular structure*” OR “porous structure*” OR “architected structure*”) AND TITLE-ABS-KEY (“additive manufacturing” OR “3d-print*” OR “3D print*”) AND TITLE-ABS-KEY (“biomedic*” OR “biosanitary” OR “biotechnology” OR “bioengineering” OR “biomechanic*” OR “medical” OR “sanitary” OR “clinical”) AND ALL (“sustainabili*” OR “carbon dioxide” OR “carbon emission*” OR “energy efficiency” OR “fossil fuel” OR “fuel consumption” OR “fuel saving” OR “global warming” OR “sustainable” OR “green” OR “energy-efficiency” OR “environment*” OR “material-efficiency”)

Subsequent to the entry of these Boolean equations into the respective databases, a preliminary selection of 1369 articles was obtained in WoS and 486 in Scopus.

### 2.3. Literature Selection

This section delineates the final selection process for the documents obtained in the preceding search. Initially, the inclusion and exclusion criteria enumerated in Table 1 were implemented. The implementation of the filter was executed through the utilization of database search tools, with the selection criteria including exclusively open access documents. This approach led to the exclusion of 809 documents from the WoS database and 267 documents from the Scopus database. Subsequently, the publication date filter was applied, resulting in the exclusion of an additional 108 documents in WoS and 28 in Scopus. Finally, one additional document was excluded from WoS, and four were excluded from Scopus because they were not written in English. This resulted in a final list of 451 documents in WoS and 187 in Scopus for reading.

**Figure 3:**
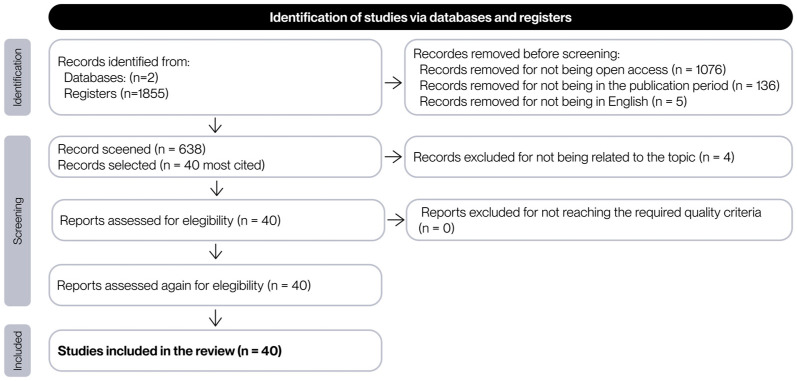
Flow chart of the process of selection of the documents found in the search.

For the purposes of this project, the reading of 40 documents is considered sufficient. Consequently, the 40 most frequently cited articles were subjected to an initial review to apply the remaining inclusion and exclusion criteria. During this review, documents that did not meet these criteria were discarded, and the next most cited document was selected in its place. The flowchart that summarizes the selection process of the literature found is presented in Figure 3.

### 2.4. Synthesis and Analysis of the Documents Included in the Literature Review

The 40 documents selected for review were subjected to an in-depth analysis, encompassing a content analysis with the objective of identifying methodologies, approaches, and findings. This process enabled the detection of recurring patterns, key contributions to the field, and potential gaps in existing research. To ensure proper understanding, the texts were reviewed to extract the data in Table 2.

The documents selected for this study were classified according to the type of contribution made, dividing them into three broad categories: state-of-the-art studies, process optimization work, and experimental research. The category with the highest representation is that of state-of-the-art studies, comprising 25 documents (62%), reflecting the community’s interest in consolidating and analyzing recent advances. This is notable despite the consensus among other works on the dearth of updated systematic compilations in this field. Secondly, 14 (35%) of the documents correspond to experimental research, in which biomedical applications have been manufactured and tested to evaluate their most important aspects, allowing the theoretical models or proposed designs to be validated in practice. A total of 10 documents (25%) were classified as process optimization studies, with a focus on the adjustment of design, simulation, or manufacturing parameters to improve the properties or performance of the applications.

**Figure 4:**
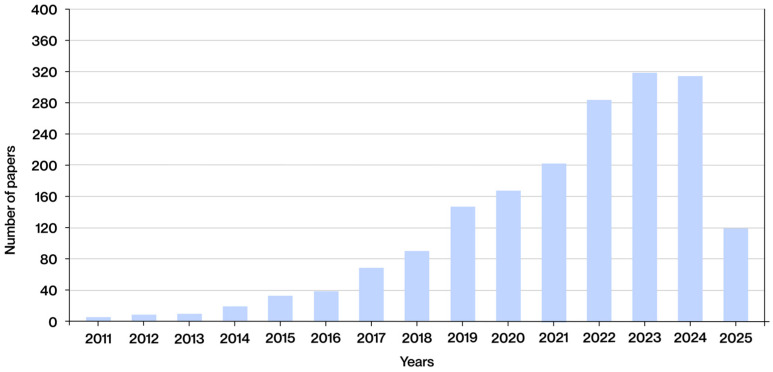
Date of publication of the documents selected from the search results, without applying the inclusion and exclusion criteria.

To accurately ascertain the publication date of the articles identified, it is imperative to revert to the literature search in which the inclusion and exclusion criteria were not implemented, resulting in the identification of 1855 documents. The publication date of these documents is displayed in Figure 4, which demonstrates a discernible upward trend in interest in lattice structures manufactured using additive technologies in the biomedical sector in recent years. From 2011 to 2016, the number of publications remained low, exhibiting a gradual upward trend. A more pronounced increase has been observed since 2017, reaching a substantial growth phase since 2019. This increase is particularly pronounced between 2021 and 2023, reaching a peak in 2023, when nearly 320 publications were documented. In 2024, the number remained high, with a slight decrease compared to the previous year, standing at 313 documents. At the time of the search, on 1 June 2025, 120 publications had been identified for that year. While this figure has yet to reach the peak values observed in previous years, it is anticipated that the total number of documents published in 2025 will continue to increase as the year progresses. These data confirm that the topic under investigation is an emerging focus of innovation and application in current manufacturing.

With respect to the geographical distribution of the studies, China is the nation with the highest number of publications, with a total of 16 documents (40%), followed by the United States, with 4 (10%). This underscores the profound commitment to development and innovation in this domain, aligning with the historical role of these two nations as leaders in global technological research. Europe has demonstrated a notable level of engagement, with nations such as the Netherlands and England particularly noteworthy, each contributing three publications (8%). Additional contributions have been made by Germany, Switzerland, Italy, Spain, Poland, Ireland, Belgium, and Finland. This commitment is further evidenced by the adoption of initiatives such as the Horizon Europe program, which underscores the region’s dedication to scientific advancement. In addition to China’s leadership, the United Arab Emirates, Pakistan, and Qatar have made significant contributions, highlighting the growing technological advancement of Asian countries. A subsequent investigation yielded a single publication from Australia, a nation with a distinguished technological legacy, notable institutions of higher education, and vibrant innovation hubs.

It is imperative to acknowledge that the identification of knowledge gaps presented in this review is not based on subjective assessments or arbitrary judgments. Rather, it is the result of a systematic procedure applied to the selected studies. In each study, the sections in which the authors themselves outlined the limitations of their work, made recommendations, or proposed future lines of research were analyzed. This analysis demonstrates that, despite the progress achieved, substantial deficiencies persist in the extant literature. These deficiencies include the absence of standardization in mechanical and biological tests, the dearth of long-term in vivo validations, the paucity of consideration for clinical factors in the design of structures, the lack of comparative methodologies that integrate materials, topologies, and manufacturing processes, and the inadequate coverage of certain types of geometries and applications. The aforementioned gaps, which have been repeatedly highlighted by the authors reviewed, underscore the necessity to establish common reference frameworks and to expand research into scenarios and parameters that have remained largely unexplored in the field of lattice structures for biomedical applications.

## 3. Results

### 3.1. AM Technologies

As previously stated in the introduction, the field of advanced manufacturing engineering has recently identified the fabrication of complex geometries with high precision and flexibility as a primary focus of research. In contradistinction to conventional subtractive processes, AM enables the fabrication of components through the methodical deposition of material layers, one on top of another, directly from a digital model. This technological advancement has led to the emergence of novel possibilities in the domains of component design and development, particularly for those components characterized by intricate internal structures. These characteristics render it a particularly suitable tool for the fabrication of lattice structures, whose morphology necessitates a high degree of design freedom and the capacity to produce geometries that are challenging to obtain by alternative means [6,12].

The standardization and classification of different AM technologies has been established by the international standard ISO/ASTM 52900:2021, which defines AM as “the process of joining materials to manufacture objects from 3D model data, typically layer by layer, as opposed to traditional subtractive or forming methods.” Consequently, this document serves as the international reference framework for the terminology, general principles, and classification of AM technologies. This classification does not rely on commercial criteria or specific manufacturer designations. Rather, it establishes seven main categories based on physical and operating principles, allowing for clear and standardized identification in any technical or scientific context [1].

The following Table 3 offers a concise overview of the seven categories identified by the standard. Tank photopolymerization, which is further subdivided into the categories of stereolithography (SLA), digital light processing (DLP), and continuous liquid interface production (CLIP), utilizes a liquid photopolymer that is solidified by a controlled light source. In the domain of material jetting, the subcategory of Polyjet and material jet modeling (MJP) involves the precise deposition of microdroplets of photopolymer, which are subsequently cured using UV light. Material extrusion, classified into fused filament fabrication (FFF), and atomic deposition manufacturing (ADAM), involves the melting and deposition of thermoplastic filaments through a nozzle, offering an economical and versatile alternative. Bonding agent jet, comprising binder jetting (BJ), multi-jet fusion (MJF), and selective powder jetting (SPJ), utilizes a liquid that binds the particles of a powder bed. Selective laser sintering (SLS), selective laser melting (SLM), direct metal laser sintering (DMLS), and electron beam melting (EBM) are collectively referred to as powder bed fusion (PBF). Direct energy deposition, comprising laser forming and powder deposition (LENS), electron beam melting with powder or wire feed (EBAM), laser material deposition (LMD), and arc and wire AM (WAAM), facilitates the addition of material in powder or wire form, which is melted by an energy source. Finally, sheet lamination, which is further subdivided into laminated object modeling (LOM) and ultrasonic AM (UAM), involves the superimposition and cutting of adhered or welded sheets [1,7,51].

In addition to the seven primary categories delineated in the standard, the extant literature has identified manufacturing methodologies that, while not explicitly included in this classification, adhere to the fundamental principle of AM: the generation of geometries through the controlled addition of material layer by layer. These methodologies include techniques such as direct ink writing (DIW), which is based on the direct extrusion of inks with specific rheological properties [13]; biological 3D printing (3DBP), which aims to deposit living cells and biomaterials [14]; and 4D printing (4DP), which incorporates smart materials capable of modifying their shape or properties after manufacture when exposed to external stimuli [15].

**Figure 5:**
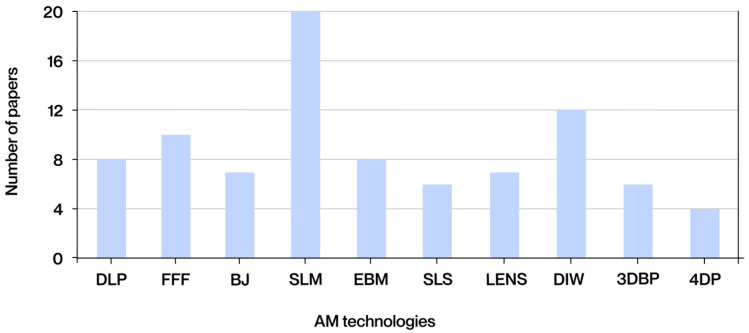
Technologies most frequently found in the literature obtained.

The graph in Figure 5 below illustrates the frequency with which the various AM technologies are mentioned in the analyzed documents.

In addition to the technologies illustrated in the graph, other manufacturing methodologies have been identified in the extant literature. Despite being mentioned in fewer documents, these methodologies are nevertheless relevant due to their applications in the biomedical sector. These include melt electrowriting (MEW), a hybrid technique combining the principles of 3D printing and electrospinning. MEW enables the precise deposition of molten microfibers, facilitating the fabrication of structured scaffolds for biomedical applications [15]. Another relevant manufacturing technique is layer-by-layer coating, a sequential method that allows the functionalization of surfaces through the alternate deposition of polymeric or bioactive layers. This technique is commonly used to give implants antibacterial properties or to release drugs [16]. In essence, 3D fiber deposition involves the extrusion of fibers to generate three-dimensional structures. A distinguishing feature of this process is its capacity to produce continuous, reinforced fiber networks, which find application in tissue engineering [17]. Despite their exclusion from the ISO/ASTM 52900:2021 standard and their present lack of ubiquity in comparison to other manufacturing processes, these technologies signify research domains with considerable potential for innovative biomedical applications.

#### 3.1.1. DLP

DLP is an AM technology based on the photopolymerization of liquid resins through the controlled projection of structured light. The use of a digital projector enables the emission of ultraviolet or visible light patterns, which solidify entire layers of the photopolymer in a simultaneous manner [18]. This process allows for a substantially higher manufacturing speed in comparison to methods such as SLA, wherein the curing process occurs point-by-point. The utilization of complete two-dimensional images in this manner has been demonstrated to enhance efficiency and ensure high geometric accuracy, particularly in the XY plane resolution [19]. The resins utilized in these processes are typically formulated from acrylate, methacrylate, or epoxy monomers, which are selected based on the desired final properties of the cured polymer [8,18,20].

The primary benefits of DLP include its high resolution, precision in reproducing contours, and capacity to generate complex microstructures with intricate geometries. These characteristics render DLP an ideal technique for obtaining customized architectures with controlled porosity and properties tailored to the needs of tissue engineering applications [21,22]. Furthermore, the simultaneous curing of entire layers results in a substantial reduction in manufacturing times when compared to other point-beam-based technologies, such as SLA or SLS [19]. However, the expenses related to equipment and specialized resins, as well as the necessity for additional post-processing, position this technology within the medium-to-high investment range, particularly in applications necessitating high resolution or complex geometries [20].

Regarding sustainability, the DLP process possesses distinctive characteristics that merit consideration. Its high resolution and curing precision have been demonstrated to minimize the need for rework and reduce the number of rejected parts, resulting in direct savings in resources and energy during production [21]. Furthermore, the utilization of resin deposited in each layer enables low-waste manufacturing, a notable distinction from alternative methods that necessitate the recovery or disposal of excess materials. Additionally, the inherent polymerization speed of DLP technology has been shown to reduce manufacturing times, leading to a comparatively reduced energy consumption per unit of output [18]. However, the sustainability of the process is contingent upon factors such as the management of unpolymerized resins, which necessitate specific treatments to circumvent environmental impacts [18,20].

#### 3.1.2. FFF

FFF is based on the controlled extrusion of a molten thermoplastic filament, which is deposited layer by layer on a build platform following digitally defined trajectories. This process, carried out at carefully controlled temperatures, allows three-dimensional parts to be built directly from computer-aided design (CAD) models, and its simplicity has established it as one of the most widely used technologies in both industry and prototype development [1,23]. FFF supports a wide range of materials, primarily thermoplastic polymers, though composite formulations with short fibers, nanoparticles, or metallic and ceramic fillers that necessitate subsequent sintering are also employed, enabling the adjustment of mechanical, thermal, or electrical properties in accordance with design requirements [6,8].

The primary benefits of this approach include minimal operational expenses, ease of access to equipment and materials, and the capacity to produce functional components with customized geometries without the necessity for specialized tooling. This has led to its extensive utilization in research, development, and short-term production. Despite its comparatively lower resolution, ranging from 100 to 300 µm, as compared to other technologies such as photopolymerization or selective powder melting, it finds application in a wide range of structural projects. In most cases, parts manufactured by FFF can be used without post-processing, except for surface or dimensional adjustments when stricter tolerances are required [1,6,51].

With regard to process sustainability, FFF is distinguished by a straightforward, low-maintenance technical infrastructure, which curtails the consumption of resources linked with equipment preparation and operation. The thermo-mechanical nature of the process, predicated on localized heating of the filament and its layer-by-layer deposition, implies a reduced energy demand in comparison with techniques that necessitate high-power sources, such as lasers [18]. However, the sustainability of FFF may be constrained by the necessity for support structures in complex geometries, which increase the volume of material utilized and the duration of printing processes. Proper process planning, incorporating aspects such as part orientation and the optimization of parameters like layer height and extrusion speed, is imperative to mitigate these effects and curtail both waste and reprints [20].

#### 3.1.3. BJ

BJ is an AM technique that involves the selective deposition of a liquid binder onto a bed of powder, forming successive layers that reproduce the three-dimensional geometry defined by a digital model. A print head, analogous to that of an inkjet printer, deposits the binder in specific areas, causing the particles to adhere locally, while a new layer of powder is spread after each cycle. This technology demonstrates compatibility with a wide variety of powder materials, including metals, ceramics, polymers, and composites, provided these materials exhibit suitable flow and packing density properties. Depending on the type of material and application, the manufactured parts often require heat or infiltration treatments for consolidation [12,24].

The primary benefits of this technology include the capacity to manufacture large-volume components without the need for support structures, due to the inherent properties of the powder, its high manufacturing velocity, and its capability to print multiple components in a simultaneous manner. This technique is particularly well-suited for short production runs and functional prototypes, especially in cases where the materials are challenging to process using alternative additive technologies. The typical resolution of the process is between 50 and 100 µm, allowing for the capture of fine details. However, it does not achieve the precision of methods such as SLM or SLA. The cost of the process is relatively moderate, primarily due to the reuse of unbound powder and the simplicity of the equipment. However, additional costs arising from the post-processing required to achieve the final properties must be taken into account [12,51].

In the context of the BJ process, sustainability is predominantly associated with its capacity to efficaciously employ unconsolidated powder, which can be recovered and reused in subsequent manufacturing cycles, thereby diminishing material waste [22]. Moreover, as it does not necessitate high-intensity energy sources for melting the material, energy consumption per piece is relatively low, placing BJ in an advantageous position from an environmental perspective [23]. However, it is imperative to consider the environmental implications of the use of binding agents, as their handling and disposal can generate chemical waste that necessitates specialized treatment. Moreover, post-processing steps have been shown to increase energy and resource consumption, thereby mitigating some of the initial sustainability benefits [24].

#### 3.1.4. SLM

SLM utilizes a high-energy laser to consolidate metal powders in a localized manner. The process is meticulously executed in a controlled environment, where a roller or scraper distributes fine layers of powder onto the platform. A laser scans and selectively melts the areas defined by the digital model, solidifying the material immediately. Subsequent layers of powder are applied in a descending order, with the platform repeatedly descending to allow for the application of each new layer. This process is repeated until the part is fully completed. This procedure enables the fabrication of components with high density and mechanical properties analogous to those of conventional materials [12,25].

SLM primarily utilizes metal powders, including stainless steels, titanium, and aluminum alloys. These powders must meet stringent criteria concerning fluidity, morphology, and particle size to ensure uniform melting. The primary benefit of this method is its capacity to manufacture components with intricate geometries, exhibiting resolutions ranging from 80 to 250 µm. This renders it well-suited for the fabrication of customized or high-performance functional components. Despite the substantial expense associated with equipment and operational costs, this is counterbalanced by the production of densified metal components that do not necessitate the use of tools or molds. This process reduces development times and facilitates the reuse of unsmelted powder [6,26].

In the SLM process, sustainability is contingent upon the high energy consumption resulting from the utilization of high-power laser sources and the necessity to maintain controlled inert gas atmospheres, which amplifies the environmental impact of the manufacturing cycle [6]. However, a significant advantage from a material point of view is the possibility of reusing a large proportion of the unmelted metal powder in successive cycles, provided that quality controls are applied to ensure the stability of its physical and chemical properties [12]. This enhanced recyclability has been demonstrated to lead to a substantial reduction in material waste. However, it is important to note that this process necessitates the implementation of additional screening and monitoring procedures, which also incur resource consumption [29].

#### 3.1.5. EBM

EBM is a technology in which a concentrated beam of electrons serves as the energy source, enabling the selective melting of layers of metal powder. The process is conducted within a vacuum chamber, a critical component for generating and manipulating the electron beam, and follows a layer-by-layer approach analogous to other powder bed-based methods. A distinguishing feature of EBM is the preheating of the powder bed prior to melting, which reduces thermal gradients and helps minimize residual stresses. This technique is particularly well-suited for the manufacturing of components made of titanium alloys, cobalt-chromium, and nickel superalloys. In this process, powders with spherical morphology, controlled particle size, and high purity are utilized [6,7,12].

The primary benefits of this process include the ability to produce dense and homogeneous metal parts with high mechanical strength and low internal stresses through the use of preheating. Its resolution, ranging from 50 to 100 µm, facilitates the fabrication of functional elements with intricate geometries and regulated metallurgical properties. Furthermore, the utilization of a vacuum environment during the process serves to impede the oxidation of the material. Notwithstanding the exorbitant expenses associated with equipment and operational expenditures, EBM facilitates expeditious manufacturing of medium-sized components and enables concurrent production of multiple parts, rendering it a viable option for applications that prioritize structural integrity and material quality [6,26].

In the context of EBM, the sustainability of the process exhibits distinct characteristics in comparison to other PBF techniques. The utilization of an electron beam as an energy source, under vacuum conditions, facilitates efficient fusion with reduced residual stresses in the component, thereby diminishing the necessity for subsequent heat treatments and, consequently, the energy consumption associated with post-processing [6,12]. Moreover, as in SLM, unconsolidated powder can be recovered and reused to a significant extent, thereby reducing material waste and optimizing resource utilization. However, the process of maintaining a vacuum environment throughout the manufacturing cycle necessitates significant energy consumption. Moreover, the stringent control of process parameters requires a more complex infrastructure, which can lead to an augmented environmental impact [6].

#### 3.1.6. SLS

SLS consolidates fine particles through laser-induced thermal sintering, without achieving complete fusion of the material. The process initiates with the application of powder in layers on a mobile platform. Subsequently, a laser scans each section of the three-dimensional model, generating heat through surface diffusion to bond the particles. The process is repeated until the final part is formed, following a similar pattern to other powder bed-based technologies [25].

The most common materials utilized in SLS are powdered polymers, including polyamides, polyether ketone ketone (PEKK), and polystyrene. However, composites, and in certain instances, metals or ceramics, can also be employed, contingent on specific process adjustments. A primary benefit of this method is the elimination of support structures, as the powder itself functions as a support medium. This characteristic enables significant geometric flexibility, facilitates the creation of internal cavities, and allows for the integration of movable components. Furthermore, it offers a favorable combination of resolution, mechanical properties, and dimensional stability in polymer parts, as well as the possibility of using recyclable materials, making it ideal for advanced prototyping and short runs [6,22].

In the SLS process, as in the two previous processes, sustainability is related to the efficient use of polymer powder. A significant portion of the residual material can be recovered and reused in subsequent cycles, thereby reducing waste [22]. From an energy standpoint, SLS necessitates considerable expenditure due to the preheating of the chamber and the continuous use of high-powered lasers, thereby increasing its environmental impact [22]. Conversely, post-processing activities, such as sandblasting or the elimination of binders, entail augmented energy expenditure and yield by-products that necessitate proper management [25].

#### 3.1.7. LENS

LENS uses a high-power laser to melt metal powder deposited directly through nozzles that direct the flow to a localized melting point. In contrast to powder bed-based processes, the material is fed in real time onto the substrate surface or onto layers that have previously undergone solidification, thereby enabling layer-by-layer construction of the desired geometry. The movement of the laser head and nozzle is guided by the CAD model, while the platform maintains a fixed position or adjusts its height with each layer [25,51].

LENS primarily utilizes metal powders, including steels, titanium alloys, and nickel superalloys, necessitating precise regulation of particle size and fluidity. The primary benefits of this material include its capacity for producing dense, large-volume metal components and its aptitude for repair and coating applications. The potential for modulating the composition of the powder during the process engenders a degree of flexibility in the fabrication of multi-material components or components with functional gradients. Despite its relatively lower typical resolution (250–500 µm) compared to other additive techniques, it is deemed suitable for structural applications. Its high cost is counterbalanced by its versatility and industrial potential [6,24,26].

In the LENS process, sustainability is strongly conditioned by the high energy consumption associated with the use of high-power lasers and the need for inert gas protection systems, factors that increase the environmental footprint [26]. However, the direct additive nature of LENS offers significant advantages in terms of material efficiency. This is due to the fact that the metal powder is deposited only in the necessary areas of the part, which significantly reduces waste compared to subtractive techniques [24]. Moreover, its capacity to repair and re-functionalize damaged metal components is a pivotal aspect from a circular economy perspective, as it prolongs the service life of complex parts and mitigates the necessity to manufacture new components. However, the handling and recovery of unused powder necessitate additional screening and conditioning processes, which are costly in terms of resources and energy [24,26].

#### 3.1.8. DIW

DIW is based on the controlled extrusion of inks or pastes with rheology adapted to maintain their shape after deposition. In contrast to the FFF process, which operates at elevated temperatures, the present method is conducted at ambient or moderate temperatures. This enables the layer-by-layer fabrication of three-dimensional structures using nozzles that follow digital trajectories. The formulation of the ink is pivotal, as it must possess viscoelastic properties that ensure both its extrusion and immediate geometric stability [21,27].

DIW has been demonstrated to exhibit compatibility with a broad array of materials, encompassing ceramic pastes, loaded polymers, hydrogels, bioinks, metal compositions, and intricate functional formulations. The advantages of this method include the ability to process heat-sensitive materials, technical simplicity, low equipment cost, and the possibility of manufacturing customized structures with high geometric freedom. Typically, its resolution is less than 200 µm, a measurement determined by the nozzle diameter and ink properties. The necessity of subsequent consolidation phases, such as drying, sintering, or chemical/thermal curing, is contingent upon the material’s composition [8,15,26,28].

In the case of DIW, the sustainability of the process is linked to its low energy consumption, since the ink extrusion is carried out at relatively moderate pressures and temperatures, without the need for laser sources or vacuum systems with high environmental costs [28]. Furthermore, the process deposits only the volume of ink required for the designed geometry, thereby reducing material waste and increasing resource utilization [8]. However, the sustainability of DIW also depends on the management of unused or surplus inks, which in many cases include solvents or chemical reagents that require proper treatment to minimize their environmental impact [21,26].

#### 3.1.9. 3DBP

3DBP is aimed at creating three-dimensional structures through the controlled deposition of bio-inks composed of hydrogels, biopolymers, cell suspensions, or biomolecules. Its applications include tissue engineering, biomedical research, and pharmacology. In contrast to DIW, 3DBP involves the incorporation of living cells into the printing material, necessitating gentle conditions to ensure their viability. The process can be carried out by pneumatic or piston extrusion, jet injection, or laser-assisted printing. It is based on layer-by-layer deposition guided by digital models [21,22].

This technology facilitates the utilization of a diverse array of natural and synthetic bio-inks, a significant proportion of which have been functionalized with nanoparticles or growth factors. A notable benefit of this approach is its capacity to engineer intricate structures that systematically arrange cells in designated positions. This capability confers a high degree of spatial control, thereby enabling the replication of authentic biological environments. The typical resolution, which is the ability to distinguish between two adjacent points, varies between 50 and 300 µm depending on the method used. The most accurate methods are jet and laser systems. Despite the substantial expense associated with equipment and consumables, their cost-effectiveness in specialized applications is noteworthy. These structures frequently necessitate post-processing procedures, including cross-linking, cultivation under controlled conditions, or functional stabilization [15,29,51].

In the domain of 3DBP, the sustainability of the process is contingent upon its capacity to function under mild conditions, which curtail energy consumption by eschewing high-power sources such as lasers or electron beams [51]. The localized deposition of bioinks enables the highly efficient utilization of material, as only the volume necessary for the construction of the tissue construct is printed, thereby minimizing waste. Furthermore, the additive nature of the process enables the direct fabrication of customized biological scaffolds and models without the need for molds or intermediate processes, thereby reducing waste generation and resource expenditure on tooling [15,51]. Nevertheless, the sustainability of 3DBP is contingent upon the limited shelf life of many bioinks. The handling and storage of bioinks necessitate controlled conditions, which can result in increased energy consumption. Additionally, there is a requirement for auxiliary sterilization and environmental control equipment [29].

#### 3.1.10. 4DP

4DP represents an evolution of AM, incorporating the dimension of time through the use of smart materials capable of modifying their shape, properties, or functionality after printing in response to external stimuli such as temperature, humidity, pH, or magnetic fields. Despite the fact that the manufacturing process is based on conventional techniques such as photopolymerization, extrusion, or ink deposition, the key lies in the use of programmable materials that allow the controlled transformation of printed structures under specific conditions [19].

The materials employed in this study encompass shape memory polymers, sensitive hydrogels, responsive elastomers, composites with functional nanoparticles, and multicomponent systems. The selection of these materials is guided by the anticipated stimulus and the requisite properties. The advent of additive printing technologies, such as FFF and SLA, has led to the adaptation of these processes to a variety of materials. A notable advantage of these materials is their capacity to self-assemble or transform without the need for mechanical intervention, a property that holds significant potential for applications in soft robotics, tissue engineering, and responsive devices. The resolution of the material is contingent upon the fundamental process and the material’s capacity to respond at the micrometer scale. While the initial cost of the equipment is comparable to that of conventional methods, the ongoing expenses associated with material development and research contribute to an elevated operational cost [8,20].

#### 3.1.11. Comparative Summary

Following a thorough analysis of the predominant AM technologies identified in the reviewed literature, Table 4 offers a concise summary, emphasizing their salient characteristics and synthesizing the fundamental aspects of each technology in terms of materials employed, advantages, limitations, achievable resolution, and estimated cost. The objective of this study is to provide a comprehensive overview of the available technologies, facilitating a comparative analysis and serving as a guide for selecting the most appropriate technology according to the specific needs of each application.

### 3.2. Fabrication Materials

The functionality and success of lattice structures in biomedical applications are contingent not only on their geometric design, but also on the materials from which they are manufactured. In the field under consideration, requirements such as biocompatibility, bioactivity, mechanical strength, controlled degradability, and integration with host tissue, among others, impose challenging criteria that cannot be met solely by structural topology. The capacity of additive technologies to process a diverse array of materials has enabled the translation of these designs into practical applications. However, the selection of material remains a pivotal limiting factor. Consequently, the material selected dictates critical properties, including cell response, bone integration velocity, and in vivo dimensional stability [22].

**Figure 6:**
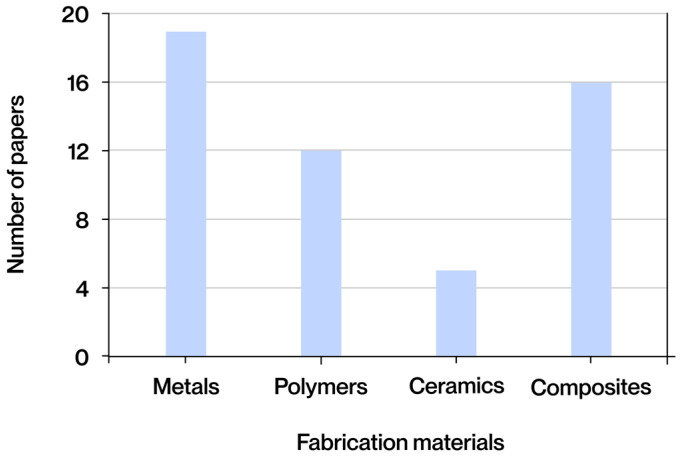
Fabrication materials most frequently found in the literature obtained.

A comprehensive review of the extant literature reveals the identification of four predominant categories of materials that are utilized repeatedly in manufacturing processes that employ additive technologies. The classification system employed in this study is based on the fundamental nature of the materials utilized, with distinctions made according to their base composition. This classification differentiates between metals, polymers, ceramic materials, and composite materials. Each of these possesses a distinct set of properties, applications, and limitations that influence the structural design and functional performance of the manufactured parts. As illustrated in Figure 6, the frequency of occurrence for each material group across diverse documents obtained from the extant literature is depicted.

Metals, the most common category found in the literature, stand out in AM due to their high mechanical strength, good conductivity, and ability to withstand heavy loads without deformation. They are mainly processed using SLM or EBM. Polymers, widely used for their structural diversity and processability using techniques such as FFF, SLA, or DLP, offer low melting points, compatibility with additives, and great adaptability to required properties. Ceramics, although less common, provide hardness, chemical stability, and thermal resistance, making them useful in demanding contexts despite their fragility, and can be manufactured using BJ or DLP. Finally, composite materials combine matrices (polymeric, metallic, or ceramic) and reinforcements (fibers, particles, or nanoadditives) to optimize rigidity, strength, and functionality, and are processed using techniques such as FFF or DIW for structural and customized applications.

In order to facilitate comparison between the main groups of additive materials, Table 5 summarizes their most relevant characteristics.

While materials can be classified into broad categories based on their composition and predominant behavior, it is crucial to acknowledge that within each category, there exist materials with properties that deviate significantly from the standard for their group. This diversity of properties enables the extension of materials’ applicability to specific functions that may initially appear to be incongruent with their inherent nature. Consequently, the properties enumerated in this table are oriented towards the biomedical sector, which encompasses a diverse array of applications, each with distinct requirements based on the function to be performed. However, there are certain properties that must be met in most cases, regardless of the specific clinical context. These include biomechanical properties, biocompatibility, biodegradability, corrosion resistance, structural porosity, the rheological behavior of the material during processing, and, in some cases, additional functional characteristics such as conductivity or response to stimuli.

The biomechanical properties of materials are determined by their capacity to withstand loads and deformations within the functional environment for which they are intended. In the biomedical field, it is imperative that materials exhibit not only structural stability, but also a mechanical behavior that is as analogous as possible to that of the tissue with which they interact. Excessive stiffness can result in stress shielding, while insufficient strength can compromise load transmission or induce mechanical failure [30]. The disparity between the elastic modulus of the implant and that of the recipient tissue has the potential to induce complications, including interference with regeneration or functional failure of the assembly [31]. Consequently, properties such as tensile strength, compression strength, fatigue strength, fracture toughness, and an elastic modulus suitable for each type of tissue are of particular importance [22]. Lattice structures enable the precise adjustment of these properties without altering the chemical composition of the material. This is achieved by manipulating parameters such as overall stiffness, relative density, or stress distribution. The subsequent section will delve into the intricacies of this geometric control strategy. Table 6 provides a synopsis of the primary mechanical properties of interest, contingent upon the type of fabric.

Biocompatibility is a pivotal property in materials intended for biomedical applications, as it dictates their acceptance by the body and their clinical viability. The concept extends beyond mere toxicity, encompassing the prevention of immune responses, the release of deleterious compounds, and the mitigation of inflammation. Additionally, it ensures stable interaction with the physiological environment [30]. This interaction can be classified as either passive or active, depending on whether it facilitates processes such as cell adhesion or functional integration [32]. The requisite level of biocompatibility is contingent upon the intended application, with higher standards applied to permanent devices than to temporary scaffolds [28].

In the context of the biomedical environment, where numerous implants are subjected to prolonged exposure to physiological fluids containing ions, proteins, and pH variations, corrosion resistance is paramount. Uncontrolled corrosion has the potential to compromise the mechanical integrity of the material and result in the release of toxic or immunogenic products [33]. The property in question is contingent upon the chemical composition, surface stability, microstructure, and potential passivation treatments or coatings. It has been demonstrated that even low levels of corrosion can result in failure over time, particularly in areas that experience high levels of stress or that are in contact with sensitive tissues. Consequently, its evaluation should encompass testing in simulated environments and electrochemical analysis under real conditions [28].

Porosity is a pivotal structural property in materials intended for medical applications, particularly when integration with living tissue or biological regeneration is the objective. The promotion of nutrient exchange, oxygenation, cell migration, and vascularization is of particular significance in the context of osseointegration and tissue regeneration [34]. However, it is imperative to note that this process also exerts a significant influence on the mechanical properties of the material. Consequently, meticulous design is essential to ensure a balanced synergy between biological functionality and structural integrity. The manipulation of these parameters, enabled by additive technologies, allows for precise control over the size, distribution, interconnectivity, and total pore percentage of the material, thereby facilitating the desired level of control. Lattice-type structures offer novel possibilities by allowing for regular or gradual porous patterns defined by geometric functions, facilitating spatial modulation adapted to the target tissue and morphological patterns such as those of trabecular bone, cartilage, or partially supported soft tissues [35].

The rheological properties of a material are instrumental in determining its behavior in a fluid or semi-fluid state during the printing process. This is particularly salient in techniques such as 3DBP or the extrusion of pastes and gels. The material must possess sufficient viscosity to ensure precise deposition and rapid solidification, thereby preserving the shape and resolution of the model [32]. This equilibrium is attained by modifying parameters such as shear-dependent viscosity, thixotropy, or viscoelasticity through alterations in concentration, temperature, chemical crosslinking, or additives. Moreover, in biomedical applications, these properties must ensure cell compatibility, biological viability, and mechanical integrity under physiological conditions [29].

In addition to the properties previously delineated, there are other salient characteristics contingent upon the type of tissue, the physiological environment, or the function of the implant. In contexts characterized by elevated heat transfer or the necessity of energy dissipation, thermal conductivity assumes paramount importance [6]. Electrical conductivity is imperative in excitable tissues, such as nerves or muscles, or in electroactive stimulation strategies to promote regeneration [15]. In the domain of 4DP, the focus is on materials that exhibit a response to external stimuli, characterized by alterations in their structural form or functional characteristics following implantation. These properties are obtained through specific formulations, functional nanoparticles, or advanced structural design, and require effective integration between composition, architecture, and environmental engineering [19].

Subsequent to the identification of the properties of the most relevant materials in the biomedical context, the subsequent step is to examine each of the established material categories in greater detail.

#### 3.2.1. Metals

The utilization of metals within the biomedical field is predominantly propelled by the necessity to guarantee mechanical stability in scenarios where alternative materials prove to be inadequate. Their exceptional resistance to cyclic loads, humidity, and fatigue renders them particularly well-suited for implants subjected to prolonged stress. Furthermore, their biological behavior can be modulated through the use of alloys, coatings, or surface treatments [17]. AM technologies enable the optimization of their structures, thereby enhancing biological integration and adjusting properties such as elastic modulus or fatigue resistance [6]. Metals exhibit superior toughness and fatigue tolerance in comparison to other materials, though their rigidity can induce stress shielding. Certain alloys necessitate control of their ionic release for reasons pertaining to biocompatibility [36].

The classification of metals utilized in AM for medical applications is contingent upon their behavior within the biological environment, which can be categorized into two distinct groups: non-biodegradable and biodegradable.

In the biomedical field, the most prevalent non-biodegradable metals are those that possess high mechanical strength, excellent chemical stability, and favorable long-term biocompatibility. The primary objective of the present study is to provide a permanent structural solution that does not require degradation or reabsorption, rendering them suitable for use in joint prostheses, dental implants, or osteosynthesis systems. Among non-biodegradable metals, the most commonly used in the biomedical field are pure titanium and its alloys, cobalt alloys, 316L steel, tantalum, and nickel alloys [17]. These materials are distinguished by their elevated mechanical strength, favorable long-term biocompatibility, and exceptional corrosion resistance, rendering them especially well-suited for permanent implants, including hip prostheses, joint prostheses, and dental implants. However, their high rigidity can result in adverse effects, such as stress shielding. Furthermore, the potential ionic release of elements like cobalt, chromium, or nickel necessitates rigorous control measures [6].

The concept of biodegradable metals represents a more recent development in the field, aimed at applications requiring temporary structural support, with the implant intended to degrade safely within the body over a limited period. This strategy obviates the need for subsequent surgical interventions to excise the material and has the potential to stimulate guided tissue regeneration [23]. Among the metals that have demonstrated the greatest potential are magnesium and its alloys, iron and zinc and their alloys. Among the group of metals that can be biodegraded, magnesium is distinguished by its biocompatibility, osteoinductive effect, and elastic modulus, which is comparable to that of bone [28]. However, its rapid corrosion in physiological environments poses significant structural challenges. Iron offers superior mechanical strength and biological tolerance; however, its slow degradation limits its practical applications. Proposed solutions include manganese alloys and porous designs. Zinc, characterized by intermediate degradability, favorable biocompatibility, and antimicrobial properties, impedes the occurrence of hydrogen during resorption. However, its inherent weakness in terms of strength and ductility necessitates enhancement through alloying processes [37].

The subsequent Table 7 offers a synopsis of the salient properties of the metals previously delineated, with particular emphasis on their characteristics when subjected to the fabrication process utilizing the AM method.

The AM of metals for biomedical applications is predominantly executed through the implementation of PBF techniques, including SLM and EBM. Titanium and its alloys have been identified as materials of choice due to their favorable processability. However, it should be noted that these materials necessitate the implementation of heat treatment to mitigate stresses and avert the formation of cracks [25]. Cobalt and tantalum alloys have also been shown to offer favorable outcomes; however, these results are contingent upon the utilization of high power and scanning strategies to circumvent thermal accumulation or the formation of brittle phases [12]. The processing of nickel alloys is a more intricate process due to their narrow thermal window, which necessitates precise regulation of energy input and environmental factors. However, functional components have been successfully obtained using SLM in small geometries [38]. The utilization of biodegradable metals introduces a set of unique challenges. Magnesium is highly reactive and prone to ignition, zinc tends to evaporate due to its low melting temperature, and iron, although stable, requires strategies to accelerate its degradation. Nevertheless, viable structures have been fabricated under controlled conditions [30].

The sustainability of these materials is contingent upon the procurement of raw materials and the subsequent management of the implant throughout its life cycle. The generation of high-purity metal powders, a prerequisite for processes such as SLM or EBM, entails substantial energy consumption and concomitant emissions [12]. However, the potential for recycling unconsolidated powder and reusing it in multiple cycles has been demonstrated to significantly reduce material waste and improve resource utilization [17]. Concurrently, the emergence of biodegradable alloys, such as Mg, Fe, and Zn, signifies a paradigm shift towards clinical sustainability. The controlled degradation of these alloys within the body obviates the necessity for surgical removal, thereby ensuring cost savings and a reduction in surgical waste generation [33]. Another salient aspect pertains to the durability of non-degradable alloys, which prolongs the useful life of implants and reduces the frequency of replacements, thereby exerting a favorable impact on the cumulative environmental footprint [35].

#### 3.2.2. Polymers

Polymers have become a prevalent material in the field of biomedicine due to their chemical versatility, low density, ease of processing at low temperatures, and good biocompatibility. These materials are composed of long macromolecular chains, which can be either natural or synthetic. The structural integrity of these materials can be adapted to offer different levels of rigidity, elasticity, or degradability. This compositional flexibility enables the design of specific formulations for soft, hard, or temporary tissues, with adjustments to the physiological environment or printing process. Despite their comparatively diminished mechanical strength and thermal stability when compared to metals or ceramics, these materials possess notable applications in fields such as regeneration, controlled release, and temporary scaffolds, thus establishing their importance in biofabrication [21].

The extensive array of polymers available in the biomedical field is attributable not only to their chemical diversity but also to the capacity to adapt them to specific clinical functions. A categorization of these materials based on their application reveals three primary groups: structural polymers for mechanical devices, polymers for tissue regeneration and scaffolding, and polymers formulated as hydrogels or cell matrices for 3DBP.

Structural polymers are utilized in applications that necessitate a specific degree of rigidity, durability, or dimensional stability. Among these, the most notable in the extant literature is polyetheretherketone (PEEK), a semi-crystalline thermoplastic with excellent mechanical strength, high thermal stability, and great chemical resistance, approved for permanent implantable applications. Its elastic modulus, which is comparable to that of cortical bone, renders it a compelling substitute for titanium in spinal, dental, and orthopedic surgery. Other materials, including polyamide 12 (PA12), find application in external prostheses, surgical guides, and support devices. These applications typically involve moderate mechanical requirements, with a focus on printing ease and geometric stability.

Polymers for scaffolding and tissue regeneration are among the most extensively studied in the biomedical field. Notably, polylactic acid (PLA) and polycaprolactone (PCL) have garnered particular attention due to their inherent biodegradability, compatibility with biological systems, and the ease with which they can be printed using the FFF technique. PLA, a more rigid material that rapidly degrades, is employed when structural support is required to be lost within weeks or a few months [21]. PCL, which exhibits enhanced flexibility and gradual degradation, is particularly well-suited for structures that necessitate maintenance of their structural integrity over an extended period, such as in the context of bone or cartilage regeneration. The material’s low melting point and adequate thermal stability enable its processing without causing damage to biological components, thus facilitating its use in hybrid platforms. Copolymers such as poly (lactic-co-glycolic acid) (PLGA) are also employed, as they permit the modulation of degradation rates and the enhancement of interaction with the surrounding environment [29].

Hydrogel polymers play a pivotal role in 3DBP and the design of cell matrices. In contrast to structural polymers, their purpose is to generate biocompatible, moist, and functional microenvironments that house living cells, transport biomolecules, or release drugs in a controlled manner [39]. The materials utilized in this study include GelMA, alginate, collagen, and chitosan, which have demonstrated excellent cell compatibility and rheological properties that are well-suited for AM applications. The formulation of these materials must address three fundamental properties: viscosity, stability, and cell viability. These materials are often combined with nanoparticles, ceramics, or drugs to enhance their functionality. Their capacity to replicate the microarchitecture of tissue renders them indispensable in the development of soft tissue models, the field of controlled release, and the realm of personalized medicine [32].

The subsequent Table 8 offers a synopsis of the most salient properties of the polymers previously delineated that are produced through the utilization of AM.

The processability of polymers using additive technologies is a significant advantage of these materials. A variety of techniques have been developed to manufacture customized scaffolds using FFF. These techniques allow for the production of customized scaffolds with PLA or PCL, combining the ease of printing with porous geometries adapted to the target tissue. To achieve optimal precision, DLP photopolymerization provides high resolution; however, it necessitates UV-compatible formulations and caution to circumvent residual toxicity. SLS enables the processing of powdered polymers, such as PA12 or thermoplastic polyurethane (TPU), without the need for support structures, thereby favoring the creation of complex internal geometries. However, this method is associated with a reduced range of biocompatible options and a diminished control over porosity or degradation. In bio-inks or soft formulations, systems such as DIW are employed, where viscosity, rheology, and rapid gelation are critical to maintain shape without compromising cell viability. In essence, the selection of printing method and parameters must be adapted to the polymer and the intended function of the device, ensuring precision, stability, and biological compatibility [40].

In the context of polymers, sustainability is predominantly associated with their provenance, processability, and end-of-life management. Some of the polymers previously mentioned, such as PLA and PCL, are derived from renewable resources or are biodegradable, which contributes to a reduction in their environmental impact and facilitates their management following clinical use [18]. This characteristic is particularly advantageous when compared to petroleum-derived synthetic polymers, such as PEKK or PEEK, whose strength and chemical stability make them more difficult to recycle and dispose of. However, the latter option demonstrates high durability in long-term applications, which in turn reduces the need for frequent replacements and may offset their initial environmental impact [32].

#### 3.2.3. Ceramics

Ceramics are defined as a group of inorganic, non-metallic materials that are characterized by high hardness, chemical stability, wear resistance, and biocompatibility in physiological environments. In contrast to metals and polymers, these substances possess a crystalline or vitreous structure, which endows them with remarkable rigidity but concomitant low fracture toughness, thereby rendering them inherently brittle. These properties impose limitations on their application in load-bearing structural contexts; however, they are particularly advantageous in environments where bioactivity or chemical inertia is imperative [22].

The utilization of ceramic materials in the biomedical sector through the implementation of additive technologies can be categorized into three primary classifications: calcium phosphates, which exhibit a high degree of similarity to bone tissue; technical ceramics, which are distinguished by their exceptional mechanical strength and chemical stability; and bioactive glasses, which possess the capacity to stimulate the formation of mineralized tissue. The mechanical, biological, and functional properties of these materials vary, and the suitability of a material for a particular application is determined by its profile [22].

Among the most widely used calcium phosphates is hydroxyapatite (HAp), a calcium phosphate that constitutes the primary mineral component of human bone. Its excellent biocompatibility, osteoconductivity, and bioactivity have firmly established it as the benchmark material in the manufacture of bone scaffolds and implants. However, its low tensile strength and fracture resistance limit its use in load-bearing applications; as such, it is often combined with other materials or used in a porous form [34]. Another notable material is tricalcium phosphate (β-TCP), which has been observed to degrade more rapidly than hydroxyapatite. This property facilitates the replacement of the material by regenerated bone tissue. Its controlled resorption makes it an intriguing option for bone defects that necessitate active regeneration stimulation [35].

Within the category of bioinert ceramics, alumina (Al_2_O_3_) and yttrium-stabilized zirconia (Y-TZP) stand out as the most significant. Both materials exhibit elevated levels of wear resistance, substantial thermal stability, and favorable biocompatibility. Zirconia demonstrates enhanced toughness due to its phase transformation mechanisms. These properties render them particularly well-suited for components subject to friction, such as femoral heads or dental crowns [17].

Finally, bioactive glasses, such as Bioglass 45S5, exhibit a distinctive property in their capacity to form a surface layer of apatite when exposed to physiological fluids. This characteristic renders them optimal candidates for functional coatings in metal implants, ion release systems, or resorbable matrices in bone regeneration [22].

The following Table 9 offers a concise overview of the most salient properties of the materials previously described.

The fabrication of ceramics presents significant challenges in comparison to metals or polymers due to the fragility of the material in its initial state, the low ductility of the material, and the necessity of high-temperature sintering. Consequently, the prevailing techniques center on methodologies that facilitate the manipulation of ceramic suspensions or pastes, such as DIW. This method enables the fabrication of complex geometries with adequate resolution; however, it necessitates meticulous regulation of the formulation, particle size, and thermal cycle to circumvent defects such as cracks or deformations. BJ is also employed in the printing of ceramic powders with removable binders [41]. However, the resulting components exhibit increased porosity and reduced density, which diminishes their strength without the implementation of supplementary treatments. In all cases, the final quality of the part is contingent upon the homogeneity of the material, the shrinkage during sintering, and the presence of microdefects [22].

The sustainability of ceramics is determined by the mineral origin of the raw materials and by the energy consumption of the synthesis and sintering processes. The production of bioceramics, including hydroxyapatite and tricalcium phosphate, necessitates elevated processing temperatures, thereby augmenting the energy consumption [22]. However, these ceramics are biodegradable, which allows for their partial reabsorption into the body and reduces the need for subsequent interventions for their removal, thus decreasing the consumption of clinical resources. Furthermore, the long service life and chemical stability of bioceramics in structural support applications help to minimize replacements and, consequently, reduce the cumulative environmental impact throughout the implant’s life cycle [34].

#### 3.2.4. Composites

Composite materials are defined as systems consisting of two or more phases with different properties. These systems are designed to combine the individual advantages of each component and generate optimized overall performance. In the biomedical field, composites have been shown to overcome the limitations of traditional single-phase materials by integrating mechanical, biological, and functional properties into a single system. This strategy is particularly useful when it is necessary to balance structural strength, bioactivity, degradability, and compatibility with cells or other biological elements [13].

The utilization of composites in biomedical applications through the implementation of additive technologies can be classified in accordance with the nature of their matrix and the function of the reinforcement. In general, polymer-ceramic, polymer-fiber, and functionalized hydrogel systems are predominant, each with a property profile that is tailored to different clinical needs.

Among polymer-ceramic composites, PLA/HA and PCL/β-TCP have demonstrated particular promise. These ceramics combine the processability and biodegradability of polymers with the bioactivity of osteoconductive ceramics. These systems are widely used in the manufacture of porous scaffolds for bone regeneration, where ceramics stimulate mineralization and improve the overall rigidity, while the polymer matrix regulates degradation and elasticity. The proportion and distribution of these elements must be meticulously calibrated to circumvent agglomeration and ensure the stability of extrusion during the printing process [29].

In the domain of fiber-reinforced polymer composites, such as PLA/carbon fiber or PU/biodegradable fibers, the objective is to enhance mechanical strength while preserving flexibility and geometric adaptability. The incorporation of fibers into the material has been shown to enhance its load-bearing capacity, dimensional stability, and durability, while maintaining low weight and good conformability. Despite their clinical application being less widespread than that of ceramic composites, they have proven useful in customized orthotics, surgical guides, and temporary support devices [42].

In the domain of 3DBP, functionalized hydrogels, exemplified by GelMA with ceramic or metallic nanoparticles, have demonstrated notable efficacy. These compounds provide soft scaffolds with additional properties such as controlled stiffness, conductivity, antimicrobial activity, and response to external stimuli. The design of these materials necessitates a balance between maintaining cell viability and ensuring the functionality of the additive, while also preserving the rheological conditions essential for extrusion printing [43].

Table 10 below provides a brief summary of the key properties of the composites discussed.

The utilization of fiber-reinforced composite materials in various industrial applications is hindered by the inherent heterogeneity of their constituent components and the incompatibility between the phases during processing. In polymer-ceramic composites, the most widely used technique is FFF, where it is essential to control the distribution of the reinforcement, the extrusion temperature, and the load content to avoid obstructions or internal defects. In fiber-reinforced systems, performance is contingent upon the orientation of the fibers and the matrix-reinforcement adhesion, necessitating specific technologies if continuous fibers are employed. Metal–ceramic composites necessitate techniques such as SLS, though they are susceptible to cracking due to thermal incompatibility. In the context of functionalized hydrogels, direct extrusion necessitates the regulation of rheology and cross-linking conditions to ensure the preservation of the printed shape and the maintenance of cell viability [19].

In the context of composites, sustainability is contingent on the inherent characteristics of their constituent elements and the compatibility between their phases with respect to processing and recycling. The integration of polymer matrices with bioceramic reinforcements has been demonstrated to enhance mechanical and biological properties, while concurrently augmenting the sustainability profile through the incorporation of bioactive and potentially resorbable materials [26]. In a similar vein, compounds derived from biodegradable polymers, fortified with natural fibers or nanoparticles derived from renewable sources, have been shown to reduce reliance on petrochemicals [19]. However, the presence of phases with vastly different degradation and recycling behaviors poses a significant challenge, as the separation of components is often intricate and limits the reuse of composites at the conclusion of their useful life [42].

### 3.3. Lattice Structures

Lattice structures have emerged as a highly promising design strategy for the manufacture of advanced biomaterials. This is due to their ability to simultaneously integrate mechanical and biological properties by modulating their internal architecture. These structures are composed of three-dimensional networks of periodically arranged unit cells, which form structures with interconnected porosity. This morphology confers a distinctive combination of functional, topological, and mechanical properties that cannot be attained in solid materials. While their origins lie in the aerospace industry, where the strength-to-weight ratio is a priority, the advent of AM has led to their proliferation in the biomedical sector by enabling designs with unparalleled geometric freedom [12]. These structures have been shown to reduce weight without compromising mechanical integrity, and they optimize fluid transport, cell anchoring, and tissue integration. By modifying structural parameters, implants can be designed with controlled stiffness, localized biomechanical response, and specific properties tailored to each medical application [8].

One of the primary advantages of lattice structures in the biomedical field pertains to their capacity to modulate mechanical properties through the alteration of geometric parameters. This approach enables the design of devices that are tailored to the specific requirements of the surrounding tissue, as evidenced by the findings presented in Table 6 [35]. The ability to manipulate the relative density and distribution of the material enables the modification of the elasticity modulus and resistance to static or dynamic loads. Consequently, the development of scaffolds and implants with varying degrees of stiffness and energy absorption can be achieved, customized to specific anatomical regions [12]. It is also noteworthy that the structure’s orientation can be engineered to align its response with the body’s principal load directions, a property that is particularly advantageous in joint systems and complex bone structures [22]. Furthermore, the specimens exhibited favorable fatigue behavior and structural stability under repetitive loads, a crucial attribute for applications necessitating prolonged durability, such as prostheses or spinal systems [36].

Porosity is a pivotal parameter in the design of lattice structures for biomedical applications, as it exerts a simultaneous influence on their mechanical, functional, and biological properties. It is defined as the fraction of the total volume occupied by voids and can be precisely adjusted through geometric design [12]. At the structural level, it directly affects relative density, elastic modulus, compressive strength, and behavior under repeated loads. An increase in porosity has been shown to reduce stiffness and load-bearing capacity, while concomitantly improving energy absorption and anatomical adaptation. The nature of this relationship is contingent upon the distribution of the pores, the thickness of the elements, and their interconnections. This approach enables the conceptualization of less rigid implants in critical areas without compromising the fundamental mechanical integrity of the structure [13,23]. From a biological standpoint, high connected porosity has been demonstrated to promote cell migration, vascularization, and nutrient exchange, which are essential for bone regeneration and tissue integration. Furthermore, porosity dictates the surface area available for cell adhesion and new tissue formation. The extant literature suggests that achieving an optimal balance between mechanical support and biological functionality is paramount. This objective is more readily attainable with FA-designed lattice structures than with conventional porous systems [22,35].

The geometry and characteristics of pores have been demonstrated to exert a substantial influence on the functional behavior of lattice structures in biomedical applications. Beyond the porosity percentage, factors such as porosity shape, size, orientation, and connectivity influence their interaction with the biological environment and their mechanical response [44]. Interconnectivity is of particular importance, as it facilitates fluid flow, nutrient transport, and cell migration, thereby enabling vascularization and waste removal from the early stages of regeneration [38]. The functionality of the implant is determined by the size and shape of the pores. Small pores impede cell penetration, while large pores can compromise stability. A controlled design enables a balanced integration of mechanical support and biological performance [26]. Furthermore, local curvature and surface continuity have been demonstrated to influence cell adhesion and tissue formation. The promotion of smooth, edge-free surfaces has been shown to foster a more biologically stable environment and to reduce stress concentrations [10].

The conceptualization of lattice structures for medical applications is predicated on two fundamental considerations: biocompatibility and the capacity for interaction with surrounding tissue. The influence of architecture on cellular response is a multifaceted phenomenon that is contingent, at least in part, on the characteristics of the underlying material. A salient factor in this regard is the specific surface area. A reticular network with high porosity and connectivity generates internal surfaces that promote cell adhesion, proliferation, and extracellular matrix formation. These surfaces also facilitate the anchoring of the implant to the host tissue [24]. The surface microarchitecture exerts a significant influence on cell behavior. Controlled roughness, generated during the process of AM, has been demonstrated to enhance osteoconductivity and osteoblastic response in metal implants [15], thereby promoting cell retention, initial fixation, and tissue maturation. Furthermore, these structures have been observed to function as provisional supports, thereby facilitating tissue regeneration. The influence of porous connectivity, channel orientation, and free space distribution on cell migration and growth patterns is a subject of ongoing research. These factors have the potential to align with load paths and natural growth axes, thereby promoting more efficient integration [22].

Finally, permeability constitutes an additional pivotal property that lattice structures can optimize in the biomedical field. This phenomenon is pivotal in determining the implant’s capacity to facilitate the movement of fluids, nutrients, gases, and metabolites. This process is imperative for the promotion of deep tissue regeneration and the maintenance of a physiologically active environment. In contrast to compact materials or disorganized porosities, the lattice networks under consideration permit precise control of permeability by adjusting connectivity, channel size, or flow orientation [1]. Enhanced permeability has been demonstrated to improve cellular oxygenation and facilitate the diffusion of biochemical signals, particularly during the initial phases of integration. Furthermore, the presence of well-defined flow paths has been shown to promote the removal of waste, thereby reducing the risk of necrosis or localized inflammation [24,45].

**Figure 7:**
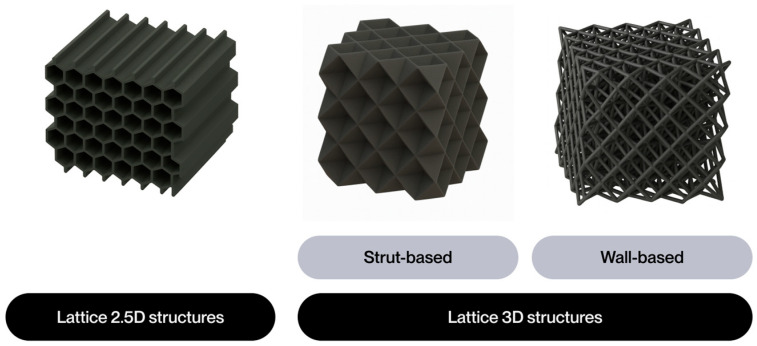
Classification of lattice structures.

Lattice structures can be classified into two primary categories based on the geometric nature of their architecture: 2.5D lattice structures and 3D lattice structures (see Figure 7). The former is distinguished by their two-dimensional periodic repetition and limited three-dimensional extension [8]. This structural typology comprises configurations such as honeycomb cells or reentrant designs, which are prevalent in applications where the principal loads act on delineated planes. In contrast, 3D lattice structures exhibit structural continuity in all three directions of space and offer enhanced load distribution capabilities, whether isotropic or anisotropic, contingent upon their design. These systems are further subdivided into two primary categories: strut-based systems, which consist of networks of interconnected struts that define nodes and straight beams; and wall-based systems, which are based on continuous walls. This latter differentiation is particularly salient in biomedical applications, as it determines both the mechanical behavior and the biological interaction of the implant [10].

**Figure 8:**
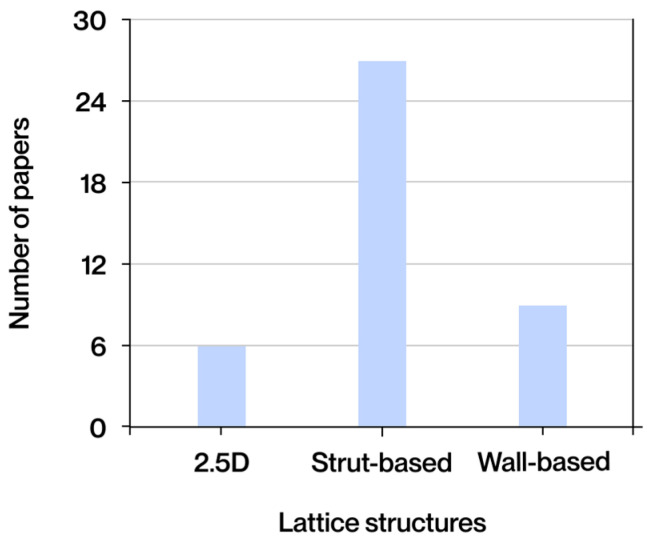
Lattice structures most frequently found in the literature obtained.

As illustrated in Figure 8, strut-based structures are the most prevalent, appearing in 26 documents. Wall-based structures are also prevalent, appearing in 9 documents. Conversely, 2.5D structures are present in a limited number of publications, indicative of their reduced applicability within the prevailing biomedical context.

It is important to acknowledge that stochastic structures, which are distinguished by a random and unpredictable distribution of porosity, have been employed in a significant portion of the extant literature. In contrast to the precision afforded by lattice structures in shaping geometry, stochastic structures exhibit a greater capacity for emulating specific biological morphologies, such as trabecular bone, while concomitantly offering reduced potential for design and functional optimization. This limitation complicates the adjustment of critical parameters, including stiffness, permeability, and localized mechanical response [24].

#### 3.3.1. Lattice 2.5D Structures

Lattice 2.5D structures are distinguished by the presence of two-dimensional periodic patterns that extend across the plane, exhibiting uniform and controlled extension in the third dimension. The design of these structures is predicated on simple geometric configurations, which are typically organized in flat layers. This organizational principle results in structures that exhibit primarily anisotropic and directional mechanical behavior. While their utilization has historically been more prevalent in lightweight structural applications within the aerospace and architectural domains, their integration into the biomedical sector has recently begun to be explored, particularly in solutions that demand flat resistance, localized flexibility, or controlled deformation mechanisms. This exploration is driven by their closed-cell configuration, which, prior to this, had not garnered significant interest due to their low permeability [22].

**Figure 9:**
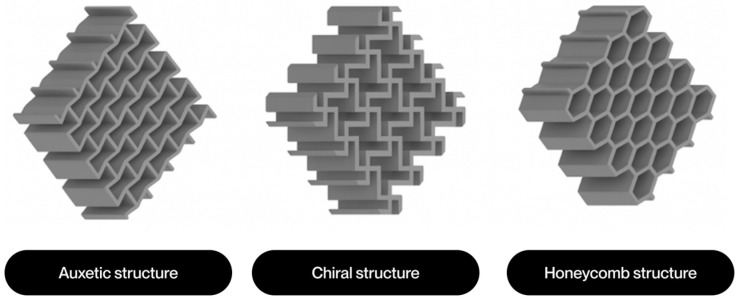
Main types of 2.5D lattice structures.

Within this category, several geometric configurations are recognized, including reentrant or auxetic structures, chiral structures, and honeycomb structures (hexagonal, square, or triangular), among others (see Figure 9). The distribution of nodes and ligaments is a distinguishing characteristic of each structure, determining its rigidity, deformability, energy absorption capacity, and response to dynamic loads [8].

However, an analysis of the extant literature focused on biomedical applications reveals that lattice 2.5D structures have been the subject of study or mention in only six documents, with the honeycomb and reentrant types being the most commonly used.

Before delving into the two types of 2.5D lattice structures, it is imperative to delineate the two types of overall mechanical behavior they can exhibit: bending-dominated behavior and stretching-dominated behavior. Bending-dominated behavior manifests when the majority of structural elements undergo deformation due to bending when a load is applied. This phenomenon typically leads to the development of more flexible structures with reduced stiffness but augmented energy absorption capacity. Conversely, stretching-dominated behavior manifests when deformations are predominantly attributable to axial tension or compression of the ligaments [3]. These structures exhibit enhanced rigidity and resistance, resulting in a more efficient mechanical response to applied loads. In order to objectively ascertain the predominant type of behavior in a 2D unit cell, Maxwell’s stability criterion can be applied. This criterion relates the number of bars (b) and the number of nodes or joints (j) using the following Expression (1):

M = b − 2j + 3(1)

In the case that M is less than zero, the structure’s behavior is dominated by bending. Conversely, if M is greater than or equal to zero, the structure’s behavior is dominated by stretching. In the context of 2.5D geometries, honeycomb configurations, for example, tend to exhibit behavior dominated by bending, while reentrant designs may present areas with mixed behavior or even locally dominated by stretching, depending on the orientation of the load and the geometry of the links. This distinction exerts influence not only on structural stiffness and strength, but also on the cell’s capacity to dissipate energy or adapt to external deformations [3].

Honeycomb structures are based on regular hexagonal cells, although square and triangular variants also exist. Their primary benefit lies in the high structural rigidity they exhibit in the direction perpendicular to the cell plane, which is accompanied by effective material utilization. However, their behavior is highly anisotropic, with low out-of-plane strength and limited deformation capacity. In the biomedical sector, this geometry has been predominantly utilized in reinforcement sheets, bone barriers, or flat supports, where the requirement is to sustain a lightweight yet resilient structure within a designated plane. The implant’s geometric simplicity facilitates its manufacture using additive layer deposition techniques, and its dimensional stability allows for precise control of the final thickness [12,35].

**Figure 10:**
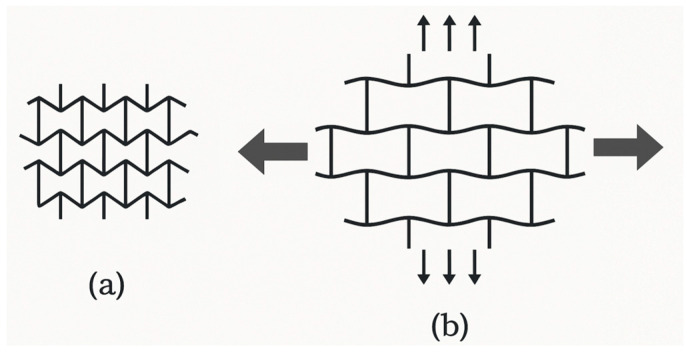
Diagram of the behavior of an auxetic structure when subjected to traction along its longitudinal axis. (**a**) Auxetic structure without loads. (**b**) Auxetic structure when subjected to traction.

In contrast, reentrant structures exhibit an inverted geometry in their ligaments, resulting in auxetic behavior, characterized by a negative Poisson’s ratio. This property enables the specimens to undergo an expansion in the transverse direction when subjected to longitudinal tension, thereby enhancing their capacity to dissipate energy, distribute stress, and adapt to irregular or deformable surfaces (see Figure 10). These qualities render reentrant structures particularly promising in applications where cushioning, anatomical adaptation, or unconventional mechanical response is required. In the biomedical field, these phenomena have been the focus of extensive research, particularly in the context of designing deformable scaffolds, functional coupling zones, and implants with dynamic mechanical behavior [8,22].

Due to the parametric and highly customizable nature of lattice structures, it is not appropriate to provide fixed numerical values for their mechanical or functional properties. The behavior exhibited by each geometry is contingent upon variables such as cell size, relative density, element thickness, structural orientation, and material composition. The vast number of possible combinations makes it impossible to establish comparable ranges of values without a specific design context. Consequently, in lieu of providing quantitative data that could give rise to interpretations that are difficult to generalize, Table 11 below illustrates the relative behavior of each geometric configuration based on its most salient properties. The evaluation is conducted using a qualitative scale that ranges from one to four asterisks (*). A single asterisk denotes low or limited performance, while a maximum of five asterisks indicates excellent performance.

The broad array of geometric configurations, materials, and manufacturing parameters inherent to lattice structures complicates the establishment of universal ranges of properties. Nevertheless, it is feasible to augment Table 11 with numerical data from authentic applications documented in the extant literature. An exemplar of this phenomenon is elucidated in the article by Dziaduszewska & Zieliński [17], which examines honeycomb structures in Ti6Al4V fabricated using SLM, designed with regular hexagonal cells measuring 1 mm in side length and 200 μm in wall thickness, yielding a porosity of 30%. This resulted in a compressive strength of 3881 MPa. The utilization of cell characteristics exhibiting marked similarity to those of Ti13Nb13Zr, a porosity of 33%, and a compressive strength of 1623 MPa was quantitatively analyzed. This discrepancy does not fully align with the disparity in properties exhibited by these two alloys, underscoring the notion that mechanical properties can exhibit substantial variation even under analogous geometric configurations and with comparable manufacturing parameters. Such variability can be attributed to various factors, including the quality of surface finish, the presence of internal defects, the extent of densification achieved, and disparities in microstructure resulting from the processing conditions. The relatively low porosity of both configurations indicates a design approach aimed at maximizing mechanical strength, which may be advantageous for applications requiring high load-bearing capacity. However, this implies low permeability and specific surface area [17].

Another exemplary illustration is provided by the study conducted by Jin et al. [15], wherein reentrant star-shaped auxetic scaffolds were meticulously designed and fabricated from polycaprolactone (PCL) through the implementation of melt electrospinning (MEW) technology. The structural units were defined with reentrant angles ranging from 110° to 140° and thick fibers with a diameter of 400 ± 20 µm, which constituted the primary mechanical structure. Subsequently, thin fibers were deposited to enhance biocompatibility and promote cell adhesion. Tensile tests demonstrated elastic deformation ranging from 3% to 6% prior to failure, exhibiting variation according to the reentrant angle. The elastic modulus exhibited a positive correlation with the narrower angles of the structures, attributed to the reinforcement in the bonding areas. However, the study did not provide a specific numerical value for the elastic modulus. A substantial enhancement in energy absorption capacity was also observed in comparison to conventional scaffolds with straight fibers, as evidenced by an increase in the area under the stress–strain curve in the tests. These results demonstrate that auxetic structures can exhibit a highly customizable mechanical response, modifiable according to geometric parameters, with direct implications for their application in tissue engineering [15].

While it is not feasible to establish universal rankings applicable to all extant variants, the analysis of specific cases allows for the identification of key trends and the assessment of the adaptability of these architectures to meet specific needs. Consequently, 2.5D structures are establishing their position as a versatile option, particularly in applications that necessitate structural rigidity, energy absorption, and anatomical adaptability concurrently.

#### 3.3.2. Lattice 3D Strut-Based

Three-dimensional strut-based lattice structures are among the most prevalent architectures employed in the design of porous materials manufactured using additive technologies. The structure consists of a spatial network of straight struts interconnected at nodes, which define a unit cell that is repeated periodically in all three directions of space. In contrast to 2.5D structures, which possess a predominantly flat geometry, strut-based structures offer true three-dimensional continuity, thereby conferring enhanced load distribution capabilities in multiple directions, more uniform porosity, a more versatile structural response, and adjustable permeability in all directions [12]. It is noteworthy that among all geometric configurations studied in the literature, strut-based structures have received the most research attention (see Figure 8).

Within this category, two major design approaches are recognized: non-parametric design, also known as geometry-based design, and parametric design.

**Figure 11:**
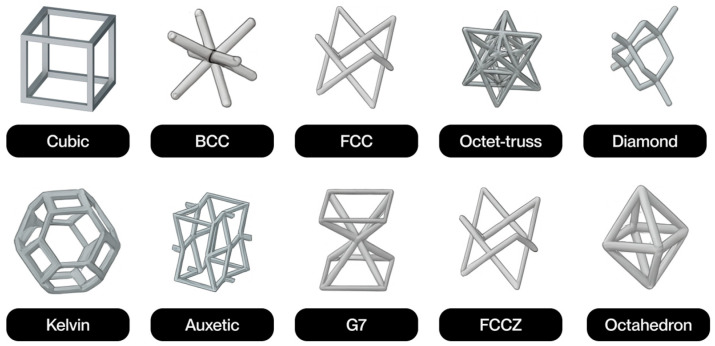
Most common types of non-parametric strut-based lattice structures.

The non-parametric design of strut-based lattice structures is based on the construction of regular unit cells with explicitly defined geometry, generally inspired by crystal lattices or classic geometric polyhedral. These cells are replicated periodically in space to form three-dimensional structures with predictable and symmetrical properties. The most representative structures in this group are shown in Figure 11, among which widely studied configurations such as simple cubic, body-centered cubic (BCC), face-centered cubic (FCC), octet-truss, diamond, Kelvin cell, octahedron, and FCC with Z-strut (FCCZ) stand out. This family of designs has been widely validated in the literature for its structural efficiency, compatibility with AM techniques such as SLM or EBM, and applicability in bone scaffolds, trabecular substitutes, and customized structural devices [36].

**Figure 12:**
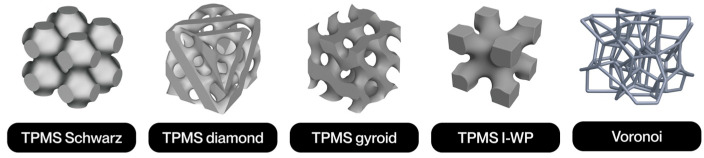
Most common types of non-parametric strut-based lattice structures.

Conversely, the parametric design of lattice structures utilizes continuous mathematical functions or computational algorithms to generate complex geometries, thereby enabling precise control over the shape, connectivity, and spatial distribution of the unit cell. The design of parametric structures typically involves the application of mathematical formulations, including but not limited to periodic minimal surfaces (TPMS), Voronoi diagrams, and other generative algorithms (see Figure 12). This geometric flexibility enables the enhancement of properties such as specific surface area, controlled anisotropy, and porous interconnectivity, rendering them particularly well-suited for applications involving the imitation of the trabecular architecture of bone or the maximization of interaction with biological tissue [36].

As was the case in the preceding instance, these structures may also exhibit global mechanical behaviors that are dominated by bending and tensile or compressive stretching. In this instance, given the three-dimensional nature of the unit cells, it is imperative to modify the preceding expression in accordance with Maxwell’s stability Criterion (2):

M = b − 3j + 6(2)

Conversely, if M < 0, the structure predominantly exhibits bending behavior, while if M ≥ 0, the structure predominantly exhibits tension behavior. In such cases, simple configurations, such as cubic or BCC, are dominated by bending, and their links tend to bend when a load is applied. This results in lower structural stiffness but greater deformation capacity. Conversely, structures such as octet-truss, FCC, or diamond demonstrate behavior characterized by stretching, given their struts’ alignment, which facilitates direct load support along their axis. The latter offer a more efficient mechanical response, with greater specific stiffness and strength, making them particularly suitable for applications where structural support under physiological loads is required. In the case of parametric structures, the behavior cannot be easily described by a single mode, as they exhibit a continuous and distributed combination of stresses [3].

The non-parametric structures that are most frequently encountered in the extant literature include cubic, BCC, FCC, octet-truss, diamond, and auxetic configurations.

The cubic structure is notable for its geometric simplicity, modularity, and compatibility with various direct printing technologies. While it does not attain the highest values of strength or rigidity, it exhibits stable mechanical behavior, high permeability, and adequate internal accessibility, rendering it suitable for applications in soft fabrics or areas with intermediate mechanical requirements [13].

The BCC structure has been shown to distribute stress more effectively in comparison to the cubic structure due to the presence of a central node, which facilitates greater homogeneity in porosity. The material has found application in the construction of scaffolding and coatings due to its ease of manufacture and satisfactory balance between mechanical properties and permeability. However, its resistance to high loads remains moderate [21].

The FCC structure offers enhanced element density without compromising porosity, thereby achieving improved structural rigidity with minimal weight. The material’s inherent symmetry enhances the multiaxial distribution of stresses, rendering it particularly advantageous for applications in joint regions. However, it demands enhanced precision in the printing process due to its geometric intricacy [6].

The octet-truss structure exhibits a combination of low density and high mechanical performance, rendering it particularly well-suited for utilization in load-bearing applications. The architecture of the structure in question has been shown to facilitate stress dissipation and to have adequate permeability. Despite the fact that its fabrication is more arduous, it is viable with contemporary technologies and has been utilized in vertebrae and mandibular prostheses [6].

The diamond structure is distinguished by its unique balance of rigidity, permeability, and specific surface area. The three-dimensional morphology of the tissue in question has been shown to distribute stresses evenly and facilitate vascularization. The material’s compatibility with processes such as EBM or LPBF renders it a suitable candidate for utilization in implants that are coated with bioactive materials, aiming to enhance osseointegration [17].

Finally, auxetic structures exhibit expansive behavior under load, thereby affording them high energy absorption capacity and adaptability to complex geometries. Despite the increased precision required for their fabrication, these devices have been successfully developed for applications such as osteochondral scaffolds and deformable joint devices, a feat made possible by high-resolution AM [15].

In the context of parametric structures, the extant literature identifies two primary categories: TPMS structures and Voronoi structures.

Strut-based TPMS structures, also known as skeletal TPMS, are defined by surfaces that have zero mean curvature at all points. These surfaces are periodic in three orthogonal directions and have no self-intersections, dividing the space into continuous labyrinthine domains. The generation of these phenomena is predicated on the application of periodic trigonometric functions to spatial coordinates (x, y, z), modulated by spatial frequency factors (w_x_, w_y_, w_z_), which serve to regulate the number of cells per unit volume. The constant C delineates the surface cut-off threshold; by varying its value, the wall thickness or the degree of porosity of the solid can be adjusted. The equations are presented in Table 12 for the TPMS structures that are most frequently encountered in the extant literature. These structures exhibit a homogeneous stress distribution, high porous connectivity, large specific surface area, and excellent structural efficiency, rendering them optimal candidates for use as bone scaffolds and tissue regeneration. However, the implementation of these models necessitates substantial computational capacity and precision in 3D printing, a necessity arising from their intricate topological structure [26].

In contrast, Voronoi structures emerge from the segmentation of space into regions based on proximity to a set of randomly selected points. This division leads to the formation of irregular cells that exhibit a trabecular morphology similar to that of bone. These structures can be modeled from real data obtained by computed tomography, with adjustments made to the distribution and shape of the cells according to the statistical values of thickness, number, and trabecular separation of cancellous bone. Consequently, the Voronoi model enables the manipulation of parameters such as pore diameter, porosity, and ligament thickness, a critical aspect for the design of implants that are tailored to specific anatomical and mechanical requirements. Despite their inherent limitations regarding reproducibility and computational cost, extant studies have demonstrated their excellent mechanical and biological performance, with results indicating an increase of up to 300% in critical load compared to other geometric configurations [21].

As in the previous section, Table 13 below presents a comparison of the most salient properties of the strut-based 3D lattice structures most found in the literature.

Notwithstanding the substantial geometric and topological variability that typifies strut-based 3D lattice structures, a considerable body of experimental research has facilitated the quantification of their mechanical properties under specific design and manufacturing conditions.

A notable example is the study by Benedetti et al. [6], which compared various configurations of BCC, BCC-Z, and Diamond strut-based lattice structures, all of which were manufactured in Ti6Al4V using SLM. The design of all structures was executed with a relative density of 30%. In conditions that were static, the diamond-type geometry yielded optimal outcomes, attaining a compressive strength of approximately 120 MPa and a Young’s modulus of 4.5 GPa. In comparison, the BCC-Z structure achieved 90 MPa and 3.5 GPa, respectively, while the BCC structure demonstrated the lowest values, with a strength of 75 MPa and a modulus of 2.8 GPa. In terms of fatigue resistance, the Diamond structure demonstrated a noteworthy advancement, exhibiting a fatigue limit close to 35 MPa at 10^6^ cycles. This is in comparison to the 25 MPa observed for BCC-Z and the 20 MPa recorded for BCC. The study demonstrated that all three geometries exhibited adequate print fidelity and dimensional stability during processing, thereby validating their viability from an AM perspective [6].

An additional illustration of this phenomenon can be observed in the study by Dziaduszewska & Zieliński [17], wherein diamond-type cell scaffolds were engineered through the use of SLM in a Ti6Al4V alloy. Among the various configurations that were evaluated, the most efficient configuration from a mechanical standpoint exhibited a porosity of 66.1% and struts with a diameter of 400 μm. These characteristics, when considered in conjunction with the compressive strength of 140 MPa and the Young’s modulus of 5.15 GPa, indicate that the material’s properties are within the range that is compatible with human bone tissue. In this configuration, up to five tests were performed, varying the thickness of the bar, with corresponding variations in porosity and mechanical properties. The study also observed good anatomical adaptation of the structure, thanks to its open architecture and favorable mechanical behavior [17].

A notable example of a more comprehensive approach is the study conducted by Davoodi et al. [12], which compared the permeability, shear stress distribution, and anatomical adaptability properties of diamond, octet-truss, and rhombic dodecahedron porous structures manufactured using SLM in Mg. The samples exhibited porosities ranging from 58% to 88%, with pore sizes varying from 500 to 1300 μm. Through simulations and experimental measurements, it was demonstrated that diamond structures exhibited a more homogeneous shear stress distribution, while octet-truss and rhombic dodecahedron configurations exhibited higher permeability, with direct implications for nutrient transport efficiency. The measured permeability values ranged from 0.29 × 10^−9^ m^2^ to 3.91 × 10^−9^ m^2^, falling within the physiological range of human trabecular bone [12].

A seminal study by Benedetti et al. [6] exemplifies the intricacies involved in the analysis of such structures. In this study, cubic lattice structures manufactured using SLM with Ti6Al4V were evaluated. Two geometric variants were analyzed: one with sharp joints between struts and another with rounded joints using fillets. Both configurations were subjected to fatigue testing, revealing that the introduction of filleted nodes significantly increased fatigue resistance, doubling the service life compared to the geometry with sharp edges. Furthermore, it was observed that fatigue cracks in structures with rounded nodes initiate in regions of slight curvature discontinuities. In geometries with straight joints, crack initiation is consistently located at the sharp edges between struts. This topological difference has been shown to enhance fatigue resistance and improve the mechanical behavior under monotonic loads by facilitating enhanced stress redistribution. These findings demonstrate how subtle geometric modifications to nodes can have a significant impact on the mechanical performance of structures [6].

#### 3.3.3. Lattice 3D Wall-Based

Three-dimensional wall-based lattice structures represent an advanced type of cellular architecture, characterized by the geometric continuity of their unit cells and their ability to biomimetically reproduce three-dimensional porous environments. These structures are defined by an architecture devoid of nodes or edges, in which the geometry flows seamlessly in space. Their mathematical and topologically continuous nature offers significant advantages in terms of stress distribution, porosity control, and the ability to emulate complex biological environments, making them a particularly attractive option for advanced biomedical applications [35].

As with strut-based 3D structures, parametric and non-parametric design approaches are recognized.

**Figure 13:**
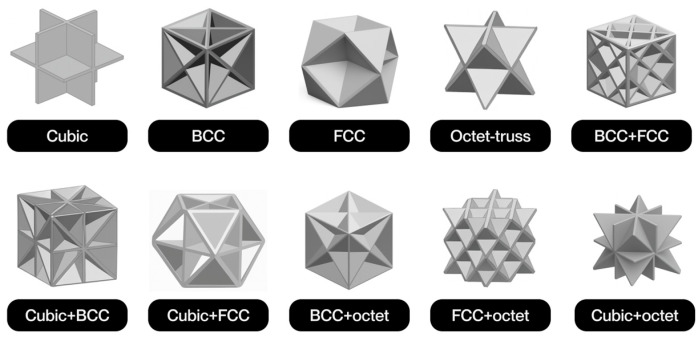
Most common types of non-parametric wall-based lattice structures.

In this case, the non-parametric design of wall-based lattice structures is generated using conventional geometric operations, such as extrusions, lofts, or revolutions of smoothed profiles. These structures feature surface continuity that mimics natural shapes. The most prevalent structures within this category include cubic, BCC, FCC, octet-truss, and hybrid configurations such as cubic+BCC and BCC+octet (see Figure 13). These structures are extensively utilized due to their geometric continuity and predictable mechanical behavior.

**Figure 14:**
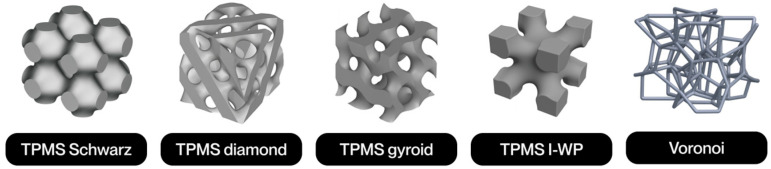
Most common types of non-parametric wall-based lattice structures.

As with strut-based structures, wall-based structures are also commonly used in mathematical formulations, mainly TPMS and Voronoi (see Figure 14), offering the same advantages of controlled anisotropy and porous interconnectivity.

These structures can also exhibit global mechanical behaviors dominated by bending or tension-compression, as defined by Expression (2). In non-parametric configurations, bending typically predominates, which implies lower relative stiffness but greater deformation capacity. Conversely, parametric structures based on mathematical functions tend to exhibit a tension-compression regime, particularly when their thickness and orientation are optimized. This behavior enables the attainment of elevated values for specific elastic modulus and strength, accompanied by more homogeneous deformations and diminished stress concentrations. This property is particularly advantageous in applications necessitating stability under cyclic or distributed loads [3].

The extant literature has identified the cubic structure as the sole non-parametric structure. This configuration is among the simplest and most well-known in three-dimensional lattice designs. The material under consideration consists of unit cells formed by six square faces, which are connected orthogonally to form a regular network in three dimensions. This geometry facilitates the modeling and manufacturing processes and enables direct control of porosity by adjusting the wall thickness. Despite its fundamental nature, this configuration persists in its frequent utilization within the domain of biomedical applications, a phenomenon attributable to its seamless integration with FA processes and its capacity to facilitate diffusion and flow paths within the implant [36].

As with previous structures, the two most common categories of parametric types identified in the reviewed literature are TPMS structures and Voronoi structures.

TPMS wall-based structures are defined in the same manner as strut-based structures (see Table 12). In this case, the implicit mathematical functions allow the space to be divided into two periodically and smoothly interconnected domains. The advantageous properties of TPMS structures in biomedical applications are attributable to their high specific surface area, porous interconnectivity, and homogeneous material distribution. These properties result in several benefits, including better integration with surrounding tissue, efficient nutrient diffusion, and more uniform internal stress distribution [4,26].

In the wall-based version of Voronoi-type 3D lattice structures, the walls of the Voronoi cells are thickened and smoothed to form continuous domains with complex topology and very high three-dimensional connectivity. These structures are of particular interest in the biomedical field due to their structural similarity to trabecular biological tissues, thereby enabling the replication of natural patterns of porosity and functional anisotropy. Furthermore, the incorporation of functional gradients or density distributions tailored to specific clinical needs, such as in customized bone prostheses or scaffolds for tissue regeneration with complex geometric requirements, is facilitated by their algorithmic generation [12].

Table 14 presents an approximate comparison of the most notable properties of the most common wall-based 3D lattice structures in the literature.

A representative example of wall-based 3D lattice structures is shown in the study by Liu et al. [46], where Schwarz-type TPMS structures manufactured using SLM in Ti6Al4V alloy were analyzed. Different configurations with a relative density of 20% were experimentally evaluated using 5 mm cells and a wall thickness of 0.3 mm. In the context of static conditions, these structures demonstrated a Young’s modulus of 1.82 GPa and a compressive strength of 60.2 MPa. Furthermore, the authors emphasized the structural behavior’s high mechanical isotropy, characterized by minimal variations in responses across different load directions. This homogeneity can be attributed to the geometric continuity of wall-based TPMS, which allows stresses to be distributed evenly. These results are consistent with the lower range of cortical bone properties, which, when combined with sufficient porous connectivity and the large specific surface area of these structures, reinforces their suitability for bone engineering applications [46].

Another example can be found in the study conducted by Pugliese et al. [35], which compared the mechanical properties of the four types of TPMS structures studied, manufactured using EBM in Ti6Al4V. The samples were analyzed across a broad spectrum of relative densities, ranging from 20% to 60%. The results demonstrated significant disparities in the topologies with respect to parameters such as stiffness, strength, and internal stress distribution. At relative densities below 50%, the Schwarz structure demonstrated the optimal overall mechanical performance, exhibiting the highest values of Young’s modulus and yield stress. This superior performance can be attributed to its predominance of stretching deformation over the bending mechanisms present in other configurations. To illustrate, at a relative density of 40%, the Young’s modulus of the Schwarz structure surpassed that of the gyroid structure by 28%, and that of the IWP by more than 40%. In contrast, the IWP topology, despite exhibiting the highest connectivity per unit volume, demonstrated the lowest stiffness and was also the most sensitive to geometric variations. In terms of stress distribution modes, the simulations revealed that in the gyroid structure, stresses were concentrated along continuous helical bands running through the volume. In the diamond structure, stresses accumulated in rings around the middle sections of the ligaments. In contrast, the Schwarz sample exhibited a more uniform distribution with lower concentration gradients, which might account for its enhanced structural performance [35].

A notable study on anatomical adaptability and permeability was conducted by Davoodi et al. [12], in which gyroid-type TPMS structures were manufactured in Ti6Al4V using SLM. These structures exhibited porosities ranging from 75.1% to 88.8%, with pore sizes varying from 500 to 1300 μm. The obtained permeability coefficients ranged from 0.29 × 10^−9^ to 3.91 × 10^−9^ m^2^, thus classifying these structures as being within the characteristic range of human trabecular bone. In terms of anatomical adaptability, the models were created using continuous modeling techniques, which eliminate vertices and discontinuities, allowing for precise fitting to complex shapes. This geometry is especially advantageous in the context of customized implants, which are derived from digital models of the patient. Therefore, the study demonstrates that the implementation of gyroid-type TPMS structures fulfills not only the functional requirements of permeability and mechanical support, but also addresses the distinct morphological demands of the bone environment for which they are designed [12].

#### 3.3.4. Optimization of Lattice Structures

The preceding sections have addressed the uniform design of lattice structures, wherein the unit cell is replicated consistently throughout the piece. While this approach is prevalent, its efficacy is constrained in circumstances necessitating local adaptation to anatomical conditions. In contrast, functional optimization techniques enable the generation of structures with variable spatial properties, which can be adapted to specific requirements such as load, bone integration, or tissue stimulation [4]. This transition is made possible by the geometric freedom afforded by additive technologies, which enables the modulation of relative density and cellular architecture within a single piece to control gradients of stiffness, porosity, or permeability. This, in turn, reduces phenomena such as stress shielding and promotes vascularization [12]. This approach draws inspiration from natural tissues, such as trabecular bone, which exhibits a hierarchical organization that responds to functional loads. Consequently, the development of optimized lattice structures aims not only to reproduce global properties but also to emulate the local behavior of the tissue, thereby facilitating the advancement of more personalized and functional medical implants [9].

In this context, topological optimization emerges as a potent instrument for the functional design of lattice structures, enabling the generation of geometric configurations that concurrently satisfy numerous structural and biological criteria. In the biomedical field, this technique is employed to adjust the distribution of material within the implant volume. The objective of this adjustment is to maximize mechanical strength, minimize relative stiffness, and promote tissue integration. The most widely used methods include the following: Solid Isotropic Material with Penalization (SIMP), Bi-directional Evolutionary Structural Optimization (BESO), and the Level Set method.

The SIMP, BESO, and Level Set topological optimization methods have been demonstrated to facilitate the design of porous structures with complex geometric configurations and functional properties adapted to biomedical requirements. The SIMP method is a computational technique that facilitates the distribution of material by assigning each element of the mesh a continuous relative density between 0 (void) and 1 (solid). This method incorporates a penalty function that favors extreme values and eliminates intermediate solutions, thereby allowing for the clear differentiation of structures between material and pore. The SIMP method optimizes rigidity with precise control of porosity [12]. The BESO method utilizes a binary representation of the material (present or absent) and an iterative addition or elimination strategy based on structural efficiency criteria, such as deformation energy. This results in designs that are robust against local minima and easily manufacturable, especially useful in implants with gradient or hierarchical structures [12]. Conversely, the Level Set method utilizes a continuous implicit function to delineate the contours of the structure, thereby regulating its evolution through design equations and enabling the representation of both smooth and complex boundaries with a high degree of precision. This formulation demonstrates a clear predilection for control over properties such as minimum thickness or curvature, resulting in the effective generation of continuous and biomimetic geometries that facilitate both mechanical behavior and cellular integration [4].

In conjunction with these topological optimization methods, the concept of functionally graded lattice structures (FGLS) has emerged. These are distinguished by regulated spatial variations in parameters such as relative density, size, or shape of the unit cell, thereby enabling local adaptation of the mechanical and biological behavior of the implant. The design of these systems is typically informed by the topological optimization methods previously outlined, which delineate the regions exhibiting the most significant structural demands. Subsequently, the geometric characteristics of the cells are modified through the application of FGLS to align with these stipulated requirements. In the biomedical field, these gradations are imperative for establishing progressive transitions between rigid support regions and more porous areas designed to promote osseointegration. A representative example is the design of femoral stems developed by Liu et al. [9], in which both axial and radial gradation strategies were implemented to adapt the stiffness of the implant to the different anatomical regions of the femur. This reduction in risk of loosening, coupled with an improvement in bone integration, is indicative of a successful design [9].

A case study comparing uniform lattice structures with optimized ones can be found in the work of Li et al. [28], which investigated the effect of topological design on the permeability and functional behavior of porous Fe scaffolds manufactured using SLM. Four geometric configurations were evaluated: two with homogeneous porosity and two with gradient porosity, all with porosity values between 58.4% and 84.8%. The findings indicated that the densest structures, or the densest parts of the structures, attained an elastic modulus of 1.22 GPa and a compressive strength of 24.5 MPa. Conversely, the designs or components exhibiting the highest degree of porosity demonstrated a modulus of 0.46 GPa and a strength of 12.6 MPa. In regard to their response to repeated loads, the scaffolds were subjected to cyclic compressive fatigue tests. It was observed that all configurations exceeded 104 cycles when loads equivalent to 40–60% of the elastic limit were applied, indicating a clear dependence between porosity and service life. Specifically, it was noted that the higher the porosity, the lower the fatigue resistance. Preliminary experimental tests and simulations have indicated a significant correlation between permeability and total porosity. However, the spatial distribution of pores has also been found to play a crucial role in this relationship. Scaffolds with higher porosity at the periphery exhibited higher overall permeability compared to those with dense in porosity, despite having similar porosities (approximately 70%). In terms of anatomical adaptability, functionally graded models offered an additional advantage, as they allowed for the replication of the natural structural variation in bone, with more porous outer regions and denser inner cores. This configuration has been demonstrated to facilitate both anatomical fit and cell migration into the implant. The article’s conclusion asserts that a topologically optimized architecture enhances not only permeability but also offers better adaptation to the surrounding bone environment [28].

Another illustrative case can be found in the study by Liu et al. [46], where gyroid and diamond TPMS structures made of Ti6Al4V using SLM were evaluated. The scaffolds were designed with cell size gradients between 0.6 and 2 mm and a relative density of 30%. The samples with 0.6 mm diamond cells demonstrated an elastic modulus of 9.90 GPa, a yield stress of 317.5 MPa, and a collapse resistance of 411.7 MPa. In contrast, gyroid configurations with equivalent cell dimensions exhibited reduced values for these parameters. The material’s elastic modulus was measured to be 7.68 GPa, its yield stress was determined to be 226.3 MPa, and its collapse resistance was found to be 331.5 MPa. The findings indicate that decreasing the cell size enhances both the stiffness and strength of the structures, underscoring the necessity to meticulously adjust the geometric parameters to attain properties suitable for the intended application without compromising porosity or internal connectivity [2].

### 3.4. Characterization of Lattice Structures

The characterization of lattice structures manufactured using AM is crucial to ensure their performance in biomedical applications. This characterization should facilitate comprehension of their mechanical behavior and enable prediction of their interaction with the biological environment. Furthermore, it should validate their functionality in real clinical conditions. The performance of these structures is contingent on numerous interrelated factors, including the base material, the manufacturing process, and the geometric architecture. However, it is also influenced by more complex aspects such as internal connectivity, response to physiological loads, and compatibility with living tissues. Therefore, it is imperative to employ a multidisciplinary approach that integrates experimental testing, computational simulations, and structural analysis to ascertain critical parameters such as elastic modulus, compressive strength, relative density, and bioactivity [6,22,41].

#### 3.4.1. Experimental Methods

The experimental characterization of lattice structures in the biomedical field encompasses a variety of techniques aimed at evaluating their morphological, mechanical, physicochemical, and biological behavior. These methodologies enable the precise determination of the properties that influence their functionality in vivo, as well as establishing correlations between structural design and the response obtained under controlled conditions.

##### Morphological Characterization Techniques

Morphological characterization techniques are imperative for the analysis of the internal geometry of lattice structures, considering parameters such as pore size and distribution, strut thickness, connectivity, and relative density. One of the most widely used tools is scanning electron microscopy (SEM), which allows observation of the surface microstructure, detection of defects, roughness, or partial collapse at cell intersections, and, in metallic structures, analysis of phenomena such as non-homogeneous solidification or oxide formation [46]. In micrometric structures, electron microscopy (EM) is complemented by energy-dispersive X-ray (EDS) analysis to map the elemental distribution in critical areas.

However, given its inherent limitations to superficial or sample sections, SEM is most often accompanied by computed microtomography (micro-CT), a non-destructive 3D analysis technique. This technique involves the projection of X-rays from multiple angles and the subsequent computational reconstruction of the internal structure. It quantifies total and interconnected porosity, strut thickness, pore size and distribution, and detects collapses or deviations from the CAD model [12]. Micro-CT is also employed to compare structures with different densities or architectures, evaluating geometric homogeneity and the continuity of internal channels. These aspects are critical in applications such as scaffolds for bone or cartilage regeneration [47].

Furthermore, its data can be integrated into simulations using finite elements or computational fluid dynamics (CFD), thereby establishing a direct link between geometric characterization and the prediction of mechanical or permeability properties [6]. In scenarios where higher resolutions are imperative, such as in ultra-thin strut structures or materials exhibiting low radiological contrast, advanced imaging modalities such as nano-CT or phase-contrast micro-CT are employed [41].

##### Mechanical Characterization Techniques

The mechanical properties of lattice structures are paramount to their application in the biomedical field, particularly in bone engineering, where they must withstand physiological loads and adapt to the biomechanical environment of the tissue. The evaluation of their behavior entails the implementation of meticulous tests that quantifiably assess parameters such as stiffness, strength, deformation, and toughness. This quantitative assessment is further enriched by the consideration of their open and anisotropic geometry.

The most common method is the uniaxial compression test, in which the structure is subjected to an increasing load until it breaks or reaches a limit deformation. The parameters that are extracted from the resulting stress–strain curves include the elastic modulus, compressive strength, elastic limit, and energy absorption capacity. These tests are designed to capture both the elastic phase and the progressive collapse or distributed plastic deformation that are typical of porous structures [24]. In bio-inspired designs or those with structural gradients, the predominant deformation mode is also analyzed, whether buckling, axial compression, or combined mechanisms [41].

Nanoindentation is a method of evaluating localized resistance in specific areas, especially in heterogeneous materials or those with functional coatings. This technique enables the assessment of hardness and reduced modulus through the controlled penetration of a diamond tip, making it particularly advantageous in structures composed of composite materials, multilayer bio-inks, or coatings containing bioactive nanoparticles, as evidenced in polymer lattices [42].

In addition to static tests, the evaluation of behavior under cyclic loads is imperative in implantable structures subjected to repeated stresses. Cyclic compression fatigue tests permit the estimation of the number of cycles to failure, the fatigue limit, and the crack propagation rate. In the context of lattice structures, the porous geometry has been observed to induce local stress concentrations, thereby favoring crack initiation even at low load levels. Consequently, the architecture, surface finish, and quality of the manufacturing process are critical factors. These tests have been applied to laser-manufactured Ti alloy lattice structures designed for orthopedic applications, where mechanical durability is essential [36].

In more specific cases, techniques such as dynamic mechanical analysis (DMA) are used to evaluate the viscoelastic response to oscillating loads, especially in polymeric materials that are sensitive to frequency or temperature [32]. In a similar vein, micro-tension or three-point bending devices have been employed on test specimens extracted from specific areas to characterize properties under anisotropic or localized conditions [48].

##### Physicochemical Characterization Techniques

In addition to morphological and mechanical analyses, physicochemical characterization provides key information on composition, crystalline structure, surface functionalization, and response to chemical agents or physiological media. These factors are of paramount importance in the biomedical environment, where chemical stability, the presence of contaminants, and interaction with biological fluids directly influence the safety and functionality of the implant.

EDS is among the most widely used techniques, typically in conjunction with SEM. This approach enables the identification and quantification of elements present on the surface or section analyzed, the detection of contamination or residues from the manufacturing process, and the evaluation of the distribution of coatings or bioactive phases [31]. Its use is particularly relevant in metal lattices manufactured by PBF, where unwanted oxides or intermetallic phases can be generated due to high temperatures.

Conversely, X-ray diffraction (XRD) is employed to ascertain the crystalline structure of the material, in addition to potential secondary phases or transitions that may be induced by heat treatment, aging, or functionalization. This phenomenon is particularly salient in shape memory alloys such as NiTi, where it enables the differentiation between martensitic and austenitic phases. Additionally, it plays a crucial role in the analysis of precipitates, including Ni_4_Ti_3_ and Ti_2_Ni, which have been shown to influence implant performance [38]. The application of this technique to ceramics or hybrid materials serves to verify the crystallization of bioactive phases, such as hydroxyapatite, following sintering or coating processes [30].

In polymeric or composite structures, spectroscopic techniques such as Fourier transform infrared spectroscopy (FTIR) allow the identification of functional groups, specific chemical bonds, and changes induced by treatments or exposure to biological media. Its utility is exemplified in bio-inks derived from collagen, gelatin, or fibroin, wherein the necessity arises to verify chemical cross-linking or functionalization with bioactive agents [43].

Finally, differential scanning calorimetry (DSC) is employed to detect thermal transitions such as melting, glass transition, or denaturation, which are pivotal for optimizing the printing, storage, and clinical use of sensitive lattices, including scaffolds for soft tissues and controlled release systems [49].

##### Biological Characterization Techniques

The biological characterization of lattice structures is imperative for the evaluation of their compatibility with living tissues and their functional potential in biomedical applications. The present study focuses on the manner in which cells interact with the microarchitecture of the material, with a view to quantifying key processes such as adhesion, proliferation, differentiation, and cell viability.

The most common in vitro tests commence with the evaluation of cytocompatibility using colorimetric assays, such as MTT, alamarBlue, or Live/Dead, which allow for the measurement of metabolic activity or cell membrane integrity. These tests are performed both in direct contact with the lattice surface and in exposure to extracts, allowing the detection of toxic compounds derived from the manufacturing process or material degradation [28,42].

The analysis of cell adhesion is typically performed through the use of optical or fluorescent microscopy, following the staining of the cytoskeleton and nucleus. This approach is frequently augmented by confocal microscopy to facilitate the observation of cell distribution in depth, a technique that is especially advantageous in the context of large-volume porous architectures. These methodologies are frequently employed in studies aimed at regenerating complex tissues, such as skin, blood vessels, and bioactive membranes [14,49,50].

In the context of tissue engineering applications, the assessment of cell proliferation entails the quantification of DNA or proteins, in conjunction with the expression of specific differentiation markers such as RUNX2, ALP, or OCN. This evaluation is typically performed using quantitative polymerase chain reaction (qPCR) or immunostaining techniques. These tests enable the determination of whether lattice is merely biocompatible or whether it actively promotes the regeneration of the target tissue [43,47]. Perfusion bioreactors are also employed to simulate dynamic physiological conditions through constant media flows, thereby enabling the study of the cellular response to mechanical stimuli such as shear stress and convective nutrient transport. This approach has particular utility in designs intended to regenerate tissues with high mechanical demands or vascularization, such as cartilage, meniscus, or the bone-tendon interface [41].

In the final stages of the research process, in vivo trials in animal models are employed to study implant integration, neotypic tissue formation, and immune response. While not universally present in the early stages, these studies are imperative for validating functional behavior under real physiological conditions [30,37]. A pertinent example is the work of Sezer et al. [30], wherein biodegradable scaffolds implanted in bone defects in rats demonstrated progressive formation of mineralized tissue without chronic inflammation [30].

#### 3.4.2. Computational Methods

In addition to experimental methods, computational characterization of lattice structures is imperative for predicting their structural, functional, and biological behavior in a non-destructive and controlled manner. The geometric intricacy of these porous architectures necessitates numerical simulations to facilitate the analysis of variables that are otherwise challenging to measure. These simulations also enable the exploration of diverse load or biological interaction scenarios and the optimization of designs prior to manufacturing. This approach has been demonstrated to reduce costs and development times, while also facilitating the customization of implants, thereby enhancing their therapeutic efficacy and clinical safety [12].

##### Mechanical Simulation

One of the most widely used computational approaches to characterize lattice structures from a mechanical point of view is simulation using the finite element method (FEM). This allows the structural response to different types of loads to be predicted with high accuracy by simulating real biomechanical conditions. The model starts with a three-dimensional geometry generated from a CAD file or, in more advanced studies, from a micro-CT reconstruction. Boundary conditions, load points, cell contact types, and material properties such as Young’s modulus or Poisson’s ratio are defined on this geometry. With this data, the FEM software calculates stress fields, deformations, and displacements in the structure [6,12].

The simulations make it possible to locate critical regions with stress concentrations, analyze collapse modes, and estimate global properties such as the effective elastic modulus or energy absorption. In periodic designs, simplified unit cell models with periodic boundary conditions are used, which reduces calculation time without compromising accuracy [22]. For more complex structures with density gradients or geometric hierarchies, complete or multizonal models are used to study stress transfer between regions with different stiffness. This strategy is particularly useful in functional scaffolds that combine load-bearing areas with areas designed to facilitate cell infiltration [41].

The validity of the results obtained using the finite element method (FEM) is typically substantiated by a comparison with experimental compression tests. However, it should be noted that the accuracy of the simulation is contingent upon the quality of the mesh, the precise definition of the contacts, and the consideration of geometric imperfections, as these can substantially influence the predictive response of the model [35].

##### Functional and Biological Simulation

In addition to structural performance, lattice structures designed for biomedical applications must ensure efficient transport of fluids, nutrients, and gases, especially in contexts such as tissue regeneration or vascular integration. The functionality in question is characterized through the use of CFD, a tool that has been demonstrated to accurately simulate the flow of biological media through the interconnected channels of the lattice. This simulation accounts for variables such as fluid velocity, local pressure, and shear stresses on internal surfaces.

The model is constructed from a representative geometry on which the fluid properties, inlet and outlet conditions, and parameters of the simulated environment are defined. The simulations facilitate the identification of flow trajectories, recirculation zones, stagnant regions, and shear gradients. These factors directly influence cell adhesion, nutrient diffusion, and the viability of implanted tissues [32].

This analysis is particularly useful in the design of lattice for vascularized bone regeneration, articular cartilage, or dynamic structures such as the meniscus, where adequate perfusion is essential. For instance, the porosity gradient geometries or the TPMS can be optimized to generate physiological shear conditions that promote cell proliferation and differentiation [35,41]. In one of their studies, these authors developed a bone implant with TPMS geometry and simulated the pulsatile flow of the culture medium using CFD, adjusting the design to achieve optimal shear levels. The implants were subsequently manufactured using SLS and evaluated in dynamic mesenchymal stem cell cultures, observing greater expression of osteogenic markers in areas of higher flow [35].

Moreover, the integration of CFD with bioreactor studies enables the validation of predictions under real conditions, thereby correlating high shear or low flow areas with cell distribution or extracellular matrix formation. In certain instances, this methodology has been employed to personalize the internal configuration of implants, meticulously designed to align with the patient’s unique fluid transport requirements, taking into account the anatomical location and physiological characteristics of the recipient tissue [14]

##### Multiscale Models and Artificial Intelligence Techniques

To capture the true complexity of the behavior of lattice structures in biomedical applications, advanced computational approaches have been developed. These approaches are based on multiscale models and artificial intelligence (AI) techniques. These strategies facilitate the integration of disparate levels of analysis and the automation of prediction and optimization processes that would otherwise necessitate exorbitant computational costs.

Multiscale models comprise independent simulations at varying scales. At the microscopic level, the behavior of the unit cell under load is studied. At the meso and macroscopic scales, global properties such as stress distribution or structural deformation are analyzed. This approach has particular utility in designs featuring functional gradients or topological transitions, as exemplified by its use in segmented scaffolds for bone regeneration or in osteotendinous interfaces [22].

Concurrently, the employment of AI has attained heightened significance in the analysis and design of lattices through the utilization of neural networks, genetic algorithms, and machine learning models. These tools facilitate the prediction of mechanical, morphological, or biological properties based on geometric or manufacturing parameters, thereby significantly reducing simulation time. A subset of models is trained with databases obtained from FEM or CFD simulations. These models are capable of instantaneously predicting properties such as elastic modulus, stress distribution, or flow. This capability exists without the need to solve physical equations [38,48].

Furthermore, AI-based generative algorithms have been employed to automatically optimize structural design, exploring thousands of combinations of geometry, density, or orientation in search of optimal configurations in terms of stiffness, permeability, or cellular response. This approach has been applied to the design of TPMS structures tailored to specific anatomical regions, combining bio-inspired design with accelerated simulations for use in personalized medicine [45].

#### 3.4.3. Characteristic Parameters of Lattice Structures

The characterization of lattice structures necessitates a quantitative evaluation that extends beyond a mere qualitative assessment of morphology or behavior. A set of quantifiable parameters is imperative to facilitate comparisons among designs, validate models, and ensure functionality. These key parameters encompass mechanical, geometric, physical, and biological properties. Collectively, these properties determine the performance of the lattice under experimental conditions and in simulated or real clinical environments. While some of these properties have been previously delineated, it is imperative to provide a comprehensive definition within the framework of lattice structures designed for applications in the biomedical sector [6].

##### Relative Density and Porosity

In the field of lattice architectures, relative density emerges as a pivotal structural parameter. This parameter is defined as the ratio between the apparent density of the porous structure and that of the solid base material. This value enables the normalization of mechanical and functional properties with respect to the initial material, thereby facilitating comparisons between disparate designs or levels of porosity. Consequently, total porosity is defined as the percentage of empty volume relative to the total volume of the structure. It has been demonstrated that both parameters directly influence the stiffness, mechanical strength, permeability, and biological interaction of the implant [12].

The relative density can be determined by measuring mass and volume, or, with greater precision, using micro-CT techniques that allow for the segmentation of solid and empty regions, even in complex geometries or with gradient topologies [15]. This technique also facilitates the detection of closed porosity or internal defects that are not visible in CAD models. Gibson and Ashby’s theory posits a nonlinear relationship between relative density and mechanical properties, whereby decreasing density leads to a reduction in elastic modulus and compressive strength, while concurrently augmenting energy absorption capacity. This phenomenon can be advantageous in applications necessitating cushioning or stiffness transition at bone–implant interfaces [46].

Porosity exerts a significant influence on the biological viability of the implant, affecting crucial processes such as cell infiltration, nutrient and oxygen diffusion, and blood vessel formation within the implant. A well-interconnected porous design has been shown to promote cell migration from adjacent tissues, allow progressive colonization, and ensure convective and diffusive transport of compounds essential for cell survival, thereby preventing hypoxic or necrotic areas [37].

##### Elastic Module and Compressive Strength

The elastic modulus and compressive strength are two of the most relevant mechanical parameters for evaluating the capacity of a lattice structure to withstand loads and deformations in a biomedical environment. The stability of these structures is contingent upon the relative density of the structure, the geometry of the unit cell, the orientation of the lattice with respect to the load axis, and the predominant type of collapse (elastic, plastic, or brittle).

The elastic modulus is defined as the effective stiffness of the lattice, that is, its capacity to resist deformation under load within the elastic range. In classical studies, it has been demonstrated that this parameter scales with relative density according to the following Expression (7):

(7) $$E=C\times {\left(\frac{\rho }{\rho_{s}}\right)}^{n}$$

In the context of the material’s deformation, the elastic modulus of the lattice, denoted by E, plays a pivotal role. C, a constant determined empirically or semi-analytically, is a function of the material’s properties and the geometry of the cell. The apparent density of the lattice, denoted by D, is a crucial variable in this analysis. The density of the base solid material, denoted by d, is also a relevant factor. The exponent n, which is determined by the cell’s topology and the predominant deformation mode, is a significant parameter in this study. Consequently, structures with behavior dominated by bending exhibit lower stiffness values compared to those that transmit load by stretching, as is the case in many TPMS architectures.

Conversely, compressive strength is defined as the maximum stress value that the lattice can withstand before significant collapse of its structure occurs. In uniaxial tests, this resistance manifests itself as a sudden drop point or as a load plateau if the structure collapses progressively. In both scenarios, the observed behavior is found to be highly dependent on the network design and potential defects that may have been introduced during the AM process [15].

##### Pore Size, Connectivity, and Strut/Wall Thickness

Geometric parameters are pivotal to the functional characterization of lattice structures, as they determine their interaction with the mechanical and biological environment. The most salient of these are pore size, porous connectivity, and strut or wall thickness, which directly influence permeability, cell migration, and structural strength.

The pore size is directly proportional to the characteristic distance of the empty space between the solid elements. The determination of the material’s composition can be made using micro-CT image analysis, microscopy, or directly from the CAD model. However, the latter method does not reflect possible deviations that may occur during the manufacturing process. An adequate pore size is conducive to cell adhesion and vessel formation, which is particularly salient in the context of bone or cartilage regeneration [28]. In designs incorporating functional gradients, spatial variability can be employed to accommodate diverse mechanical or biological demands within a single implant [28]. Porous connectivity, defined as the three-dimensional interconnection of pores, is imperative for ensuring cell viability. Quantification is achieved through micro-CT, employing metrics such as the Euler connectivity index, the number of open pores, or fluid accessibility. High connectivity facilitates the efficient transport of oxygen, nutrients, and waste, and allows for deep cell colonization, reducing hypoxic or necrotic areas [49].

The thickness of the strut or wall exerts a significant influence on the overall stiffness, stress distribution, and collapse resistance of the structure. The measurement of this parameter is typically conducted through micro-CT with density segmentation or estimation from the CAD model. However, it is imperative to emphasize the necessity for validation following manufacturing. Reducing thickness has been shown to enhance porosity; however, this can potentially compromise mechanical stability if not meticulously designed [31].

##### Surface Properties

Beyond structural and geometric parameters, surface properties of lattice structures are decisive in their biological interaction. The impact of roughness and surface chemistry on critical biological processes, including cell adhesion and differentiation, extracellular matrix mineralization, and inflammatory response, is a subject of significant interest in the field. Consequently, their characterization and potential controlled modification are indispensable components of implant functional validation.

Quantitative assessment of surface roughness can be facilitated by the implementation of methodologies such as atomic force microscopy or optical and contact profilometry. These techniques yield metrics including average roughness (Ra) and average quadratic roughness (Rq). The use of SEM for qualitative observation of micro- and nanometric topographies is also employed. This phenomenon has been observed to impact both the cellular response and the mechanical integration with the host tissue, particularly in the context of bone applications [40].

Regarding surface chemical composition, techniques such as EDS, XPS, or FTIR allow the identification of elements and functional groups, the detection of contaminants, or the validation of functionalization treatments. This analysis is particularly relevant in metallic materials susceptible to oxidation, such as Mg or Ti, or in structures treated with biomolecules, nanoparticles, or bioactive coatings designed to improve integration with the biological environment [31].

##### Bioactivity and Biodegradability

Lattice structures must not only possess adequate mechanical properties and an optimized architecture, but also exhibit bioactivity and, in many cases, controlled biodegradability, especially when intended for temporary functions or dynamic integration into host tissue.

These parameters are critical for evaluating the in vivo performance of the implant, as they determine its capacity to stimulate tissue regeneration and its integration within the biological environment. Bioactivity is defined as the capacity of a material to elicit a beneficial cellular response, including adhesion, proliferation, differentiation, or extracellular matrix formation [42].

The evaluation of these materials involves a series of in vitro tests, including the expression of specific markers such as alkaline phosphatase or type I collagen in osteoblasts, as well as the formation of apatite in simulated media such as simulated body fluid. The enhancement of this property can be achieved through the implementation of osteoconductive materials, bioactive coatings, or the incorporation of functional factors onto the surface of the lattice [42].

Biodegradability is defined as the material’s capacity to undergo progressive decomposition within the physiological environment without generating toxic by-products, concurrently with tissue regeneration. This approach is of particular pertinence in the context of temporary implants, particularly in scenarios involving bone, cartilage, or soft tissue repair. The phenomenon’s manifestation is contingent not solely on the material properties employed, but also on the geometric configuration of the lattice. This is evident in the parameters that govern porosity, element thickness, and internal connectivity. Lattices with high porosity and thin elements possess a greater surface area exposed to the fluid, thereby accelerating dissolution. In contrast, denser structures exhibit greater resistance to degradation. Furthermore, the topology can favor or hinder fluid renewal, affecting the accumulation of corrosion products or local acidification [15].

The quantification of degradation is achieved through immersion tests conducted in simulated physiological fluids, wherein mass loss, ion release, and pH changes are measured. The effect of degradation on residual mechanical properties and cell viability is also analyzed, as too rapid dissolution can compromise structural stability, while too slow dissolution can interfere with host tissue regeneration [30].

## 4. Applications

Lattice structures manufactured using additive technologies have proven particularly well suited to addressing the specific challenges of the biomedical sector, where the interaction between the implanted material and living tissue requires multifunctional, customizable, and biocompatible solutions. In this state-of-the-art development, the rationales for the incorporation of these elements have been thoroughly delineated. These rationales encompass the capacity to replicate the mechanical and topological characteristics of trabecular bone [20], along with the potential to engineer geometries that promote osseointegration, vascularization, and nutrient transport [30]. Consequently, the potential offered by these types of structures in combination with biodegradable materials, bioactive coatings, or drugs has been highlighted, extending their scope beyond structural support to active and regenerative devices [50].

In order to achieve a comprehensive comprehension of the versatility of lattice structures in the biomedical field, please refer to Table 15 below. This table provides a comprehensive list and classification of all applications identified in the 40 articles that were selected during the systematic literature review.

The classification of the applications enumerated in Table 15 is intended to address the need to organize the considerable heterogeneity of devices, tissues, and implants that is documented in the extant literature. It has been observed throughout the analysis that the applications in question are not grouped exclusively by the type of material or geometric architecture, but rather by their biomedical purpose and the anatomical or functional environment in which they are integrated. Consequently, they have been methodically organized into large functional groups, thereby facilitating the identification of common design patterns, technological requirements, and clinical approaches. The initial category, orthopedic and bone regeneration implants, encompasses both permanent prostheses and biodegradable structures designed to replace or regenerate bone tissue in weight-bearing regions. The second, tissue engineering, involves the use of scaffolds to facilitate the functional regeneration of soft tissues such as cartilage, skin, muscle, or nerve. In this process, the structure functions not as a mechanical support but rather as a biomimetic microenvironment that promotes cellular activity. The third category, implantable devices, encompasses applications in which the device’s structure does not replace tissue but rather performs a specific technological or therapeutic function, such as controlled drug release, sensorization, or temporary stents.

**Figure 15:**
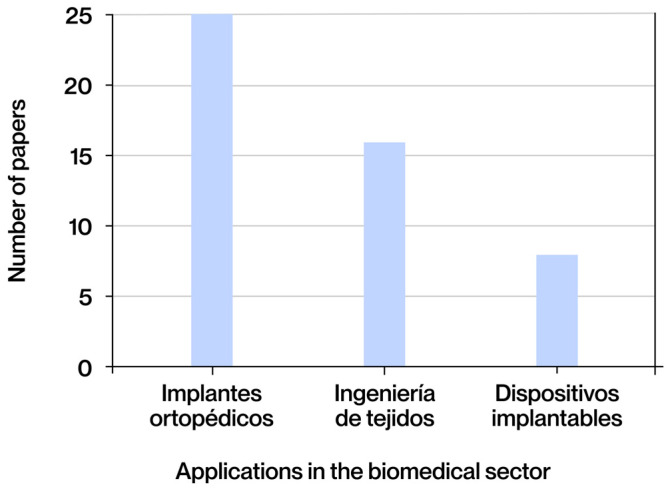
Applications of lattice structures manufactured using additive technologies in the biomedical sector most found in the literature obtained.

Figure 15 illustrates the frequency with which these applications have been investigated in the reviewed documents, demonstrating a clear predominance of studies focused on orthopedic implants and bone regeneration, followed by those aimed at tissue engineering.

In the biomedical context, it is important to note that lattice structures can be implemented at both the macroscale and microscale. Each lattice structure is designed to meet different functional requirements. Macroscale designs are frequently linked to load-bearing components, such as orthopedic implants or structural scaffolds, where the primary objectives are mechanical performance, anatomical adaptation, and long-term stability [25,30,48]. In contrast, microscale configurations are utilized to modulate the cellular microenvironment, control pore-level architecture, and influence biological responses, often within micrometric dimensions [12,36]. While both scales benefit from the design flexibility offered by additive manufacturing, they differ significantly in optimization criteria, manufacturing limitations, and validation approaches. Therefore, it is essential to consider scale as a determining parameter in the analysis and development of lattice structures for biomedical applications [26,49].

### 4.1. Orthopedic Implants and Bone Regeneration

The initial significant category of applications identified in the reviewed literature pertains to orthopedic implants, temporary replacements, and bone regeneration strategies in weight-bearing areas. This group encompasses a range of prosthetic devices, including permanent prostheses such as joint and structural prostheses, as well as biodegradable systems designed to facilitate bone tissue regeneration in localized defects, whether cranial, dental, or vertebral. These solutions address the necessity of restoring skeletal integrity in cases of trauma, tumor resection, or degenerative processes. They are distinguished by their capacity to withstand substantial mechanical loads during implantation and functionalization. In the context of prostheses, the objective is to achieve stable and long-lasting integration with the host bone. Conversely, resorbable implants are designed to degrade in a controlled manner as new bone tissue forms and matures [34].

In these applications, the scale at which the lattice structure is designed and manufactured is critical to its clinical performance. At the macroscale, these devices are principally designed to substitute for or provide support to complete bone segments that are subjected to stress. In these cases, the unit cells are millimeters in size, and the priorities are mechanical strength, anatomical fit, and long-term stability, while maintaining an elastic modulus that is close to that of the surrounding environment [25,48]. Conversely, at the microscale, applications prioritize the optimization of the immediate environment of bone and progenitor cells, characterized by structures with pores ranging from tens to a few hundred micrometers, reduced strut thickness, and high interconnectivity. These configurations are designed to control cell orientation, facilitate the diffusion of nutrients and oxygen, and modulate the biological response through topographical or functional gradients. This renders them particularly relevant in scaffolds for localized bone regeneration, small-scale defect filling, or porous coatings for metal implants [12,32].

It has been demonstrated that all large defined groups have a similar procedure when conceptualizing and carrying out a specific application. While certain aspects of this process may be subject to specific phases or influenced by the anatomical environment or intended function, the general progression of an AM-based device or implant typically adheres to a consistent sequence of stages.

In the context of orthopedic implants and bone regeneration, this process commences with the procurement of precise anatomical models, typically derived from medical imaging techniques such as micro-CT. These techniques facilitate the delineation of the geometry of the defect or region to be operated on with accuracy. According to the models under consideration, the implant is digitally designed, with consideration given to both its geometric integration and its mechanical suitability for the environment. In a subsequent phase, a thorough characterization of the structural topologies to be implemented in the design is conducted. This step is essential for selecting configurations that offer an adequate balance between mechanical properties, volumetric stability, and permeability for bone regeneration. The selection of one geometry or another is based on the analysis of the biomechanical environment and the expected distribution of loads within the implant. This characterization establishes a rational framework for decision-making in the subsequent stages of the design. Subsequent to the integration of geometric and topological characteristics, a computational simulation process is initiated to predict the structural behavior of the implant under varying load conditions. This analysis facilitates the evaluation of phenomena such as stress shielding, stress concentration, and fatigue resistance, which form the basis for an iterative structural optimization process. At this stage, key design parameters are adjusted until a configuration that meets the functional and clinical requirements is achieved. The final optimized design is manufactured using additive technologies and validated through experimental testing involving physical models and fixation environments that are representative of the intended applications. These tests are designed to verify the stiffness, cyclic durability, and overall behavior of the implant, thereby completing the process cycle [25,30].

Hip prostheses represent a well-established application in the domain of regenerative orthopedics. These devices are employed in patients afflicted with severe osteoarthritis, avascular necrosis, or complex fractures, with the objective of restoring mobility and recuperating the structural functionality of the proximal femur and pelvis. The modification of geometric properties has been demonstrated to enhance the initial fit, thereby mitigating complications such as dislocation or accelerated wear [24]. These prostheses must demonstrate durability in the face of high cyclic loads and exhibit minimal stress shielding.

A notable example is the study conducted by Buj-Corral et al. [25], in which customized acetabular cups were designed using SLM from titanium powder. Three distinct lattice configurations were evaluated: strut-based cubic, diamond, and TPMS gyroid. The objective of this evaluation was to modulate the mechanical behavior of the implant under physiological loads. The design was optimized through a combination of numerical simulations and physical model printing, followed by rigorous stiffness and initial stability tests. The geometries exhibiting enhanced connectivity exhibited an effective Young’s modulus approximating 12 GPa, a value considerably lower than that of solid titanium but more akin to that of cortical bone. This reduction in modulus potentially mitigates the risk of stress shielding. Designs with higher relative density exhibited compressive strengths in excess of 400 MPa, and exhibited enhanced stability under cyclic loads. The capacity to modify parameters such as strut size, wall thickness, and pore distribution enabled the establishment of a direct correlation between mechanical and biological properties. This enhancement of bone integration and reduction of micro-movements at the bone–implant interface are significant advancements in bone reconstruction. This case demonstrates the strategic implementation of structural modulation to achieve a balance between mechanical functionality and biological adaptation in customized hip prostheses [25].

Another noteworthy study in the field of hip prostheses is that of Davoodi et al. [12], which evaluated the behavior of different lattice structures made of titanium using SLM technology. The aim of the study was to reproduce the mechanical properties of trabecular bone and improve bone integration in customized acetabular cups. The structures in question were of the following types: strut-based cubic, diamond, octet-truss, and octahedron. The designs exhibited controlled porosity within the range of 65% to 80%, with pore sizes varying from 500 to 800 μm and strut thicknesses of approximately 300 μm. These parameters were meticulously optimized to enhance permeability and vascularization. In regard to mechanical performance, Young’s moduli adjustable between 2 and 10 GPa were achieved, depending on the relative density, as well as compressive strengths greater than 120 MPa, values that are compatible with those observed in cortical bone. Furthermore, enhanced fatigue resistance has been documented, a phenomenon attributed to the configuration of structures that feature gradual stress distribution and substantial internal connectivity. From a biological standpoint, these structural parameters elicited a favorable cellular response in in vitro cultures, accompanied by indications of osteoblastic proliferation and differentiation, with no discernible signs of inflammation. The study underscores the significance of the interplay between adjusted stiffness, high interconnected porosity, and surface roughness in enhancing the mechanical stability and bioactivity of the implant within the acetabular environment [12].

The implantation of joint prostheses is indicated for the replacement of damaged joint bone surfaces, including those of the knee, shoulder, and elbow, in patients afflicted with degenerative diseases, traumatic injuries, or severe joint wear. The objective of this research is to restore mobility and efficiently transmit loads under multiaxial stresses [12]. These components must demonstrate resilience against fatigue, wear, and loosening, adapt to complex movements, and preserve functional anatomy. The design places a premium on low-friction surfaces, anatomical anchors, and regions with adjusted stiffness. The validation process encompasses simulations, cyclic testing, and wear testing. In select cases, the clinical efficacy of customized prostheses has been examined through postoperative follow-up evaluations [35].

For this particular application, Davoodi et al. [12] developed a customized design for joint surface replacement in the knee and shoulder. This design utilizes strut-based cubic and diamond structures in titanium alloy manufactured using SLM. These structures incorporated an internal network with 75% porosity and pore diameters ranging from 600 to 800 μm, with struts measuring 300–400 μm in thickness. This configuration enabled the adjustment of the Young’s modulus of the implant within the range of 3 to 8 GPa, values that are compatible with trabecular and cortical bone. From a mechanical perspective, fatigue tests demonstrated a resistance greater than 10^7^ cycles under repetitive physiological loads, thereby confirming its suitability for this particular joint type subjected to multiaxial stresses. In terms of biological behavior, the high interconnectivity of the porous network promoted remarkable cell adhesion and osteoid tissue formation in in vitro studies, while simulations showed effective load distribution throughout the implanted volume [12].

The utilization of mandibular prostheses following resection is indicated in cases of tumors, trauma, or infection, with the objective of restoring both masticatory function and facial symmetry. These forces must be resisted, and the mandibular continuity must be maintained. Additionally, interference with the temporomandibular joint must be avoided. These models incorporate patient-specific surfaces, lightweight yet rigid internal structures, and specific fixation elements [45].

In a clinical study involving 20 patients, conducted by Davoodi et al. [12], four patients underwent surgery using prostheses designed for their anatomy using 3D printing. These incorporated strut-based diamond-type internal structures were selected for their balance between structural rigidity and osseointegration capacity. The internal geometry exhibited a controlled porosity of 70%, with pore sizes ranging from 500 to 750 μm, and strut thicknesses approaching 300 μm. These configurations reduced the effective Young’s modulus to values of 5–8 GPa, which is significantly closer to that of mandibular cortical bone. In animal models, 12 months after implantation, the implant strength was comparable to that of native bone, with complete structural integration and no signs of loosening. From a clinical perspective, patients who underwent treatment with customized prostheses demonstrated enhanced intraoperative adaptation, decreased surgical duration, and expedited functional recovery. These results underscore the potential of lattice structures not only to adjust mechanical properties but also to optimize the biological response in complex mandibular reconstructions [12].

Cranio-maxillofacial implants are designed to restore bone defects in the skull and facial region that have been caused by various factors, including trauma, tumors, or malformations. The restoration of volume, facial symmetry, and protective function is imperative. These applications necessitate high three-dimensional precision, generated from tomographic images and symmetry techniques. The design incorporates reinforcements, fixation zones, adaptive edges, and lightweight structures. Validation is a multifaceted process that incorporates simulations, anatomical testing, in vitro studies, and, frequently, clinical validation [22,24].

Dental implants are prosthetic devices that are used to restore mastication and aesthetic function in patients who have experienced tooth loss. These components are integrated into the maxillary or mandibular bone as an artificial root, and they are required to withstand cyclic loads, prevent corrosion, and promote osseointegration. The design incorporates surface roughness, stiffness-modulating microstructures, and geometries adapted to the bone bed. The validity of these findings is supported by clinical studies of functionality and osseointegration [25].

Implants for vertebral structures are used to replace vertebral bodies or to stabilize segments following resection or trauma. These elements must demonstrate the capacity to resist axial loads and promote intervertebral bone fusion. The design incorporates adapted geometries, porous surfaces for fixation, and hollow areas for bone grafts [6].

In the domain of spinal reconstruction, numerous studies have demonstrated the efficacy of customized implants fabricated using additive technologies such as SLM or EBM in titanium alloys. These solutions have employed lattice-type structures classified as strut-based cubic, BCC, FCC, diamond, octet-truss, and TPMS gyroid, selected for their capacity to withstand high axial loads while maintaining a homogeneous stress distribution [6,12,25,33]. The adopted geometries exhibited controlled porosities ranging from 60% to 80%, with pore diameters exceeding 500 μm and strut thicknesses ranging from 300 to 400 μm. These configurations enabled the adjustment of Young’s modulus within the range of 3 to 10 GPa, thereby allowing the attainment of compressive strengths exceeding 150 MPa. This achievement effectively replicates the properties of vertebral cortical bone [12]. Furthermore, its elevated internal connectivity and surface continuity have been demonstrated to promote osseointegration in highly demanding biomechanical environments, as evidenced by preclinical studies and functional simulations [6,33]. At the biological level, the combination of surface roughness and interconnected porosity facilitated progressive cell colonization and good vascularization in deep regions, contributing to the formation of new bone tissue and implant stabilization [12,25].

Applications intended for the correction of bone defects are aimed at reconstructing critical discontinuities in bone tissue, generally caused by trauma, tumor resection, or severe infections. These solutions aim to restore the structural continuity of the bone in areas where spontaneous regeneration is not feasible due to the size or location of the defect. The objective is twofold: first, to restore mechanical integrity, and second, to provide a suitable environment for tissue regeneration. In order to achieve this objective, implants must possess sufficient strength to withstand loading in the early stages of the healing process while also facilitating vascularization, cell migration, and new bone formation within the defect. The design frequently incorporates anatomical shapes adapted to the defect, central areas of high porosity to promote bone growth, and more compact peripheral structures that provide support and stability [41]. In several studies, the evaluation of these solutions has been complemented by tests on mineralization, cell infiltration, and resorption of the biodegradable material.

A notable example of bone regeneration in critical defects can be found in the study conducted by Sezer et al. [30], in which biodegradable polymer structures were implanted in critical femoral defects in rats. The scaffolds were fabricated using SLS and designed with a porosity of 70% and a pore diameter of 600 μm, incorporating a decreasing density from the periphery to the core of the implant. This structural gradation facilitated a more accurate replication of the natural trabecular environment, promoting both anatomical adaptation and uniform stress distribution. Eight weeks post-implantation, histological analyses revealed progressive bone tissue formation and partial resorption of the material, with no indications of inflammation or adverse immune response. In terms of functionality, the implant exhibited stable mechanical behavior during the regeneration process, demonstrating sufficient stiffness to withstand physiological loads from the environment without compromising biological integration [30].

Applications that prioritize bone regeneration in weight-bearing regions seek to repair defects located in areas that must withstand substantial mechanical stress from the early stages of the healing process. Such instances frequently arise in long bone diaphyses, weight-bearing joints, and vertebral and pelvic reconstructions. These devices are designed to provide stable structural support, thereby preventing collapse or displacement, while facilitating native bone regeneration. In contrast to other temporary implants, rigidity, compressive strength, and mechanical durability under cyclic loading are critical requirements from the moment of implantation [32]. In the design, configurations with high mechanical efficiency that resist axial and multiaxial loads without sacrificing porosity are prioritized. The validation of these applications is achieved through a multifaceted approach that incorporates compression and fatigue testing, in vivo studies under physiological loads, and computational simulations to analyze stress distribution [28].

A practical example is presented in Jiao et al. [41], where ceramic scaffolds with controlled porosity were developed using BJ. A series of models with variations in structural density were evaluated, and the model with a porosity of 47.7% was identified as optimal. This configuration achieved a compressive strength of 13.41 MPa and an elastic modulus of 128.64 GPa, values that approximate the mechanical behavior of loaded trabecular bone, ensuring adequate initial stability in demanding environments. The structural design comprised a wall-based TPMS network, which exhibited homogeneous stress distribution, high porous connectivity, and a specific surface area conducive to cell colonization. This design enabled the concurrent support of physiological loads from the early stages and facilitated biological integration through the formation of new bone tissue in the interconnected spaces [41].

Temporary bone substitutes are designed to provide structural support during the initial phases of bone regeneration. Subsequently, these substitutes undergo controlled degradation, and are then replaced by native tissue. These devices have been particularly beneficial in pediatric contexts, in cases of non-critical defects, and in situations where the removal of the implant is considered to be medically inadvisable. It is imperative that these devices offer adequate rigidity to rectify the defect while preserving the biomechanics of the adjacent bone. The degradation of these materials must be synchronized with the rate of bone formation, thereby avoiding premature loss of support or the persistence of residues. These designs integrate interconnected porosities, controlled densities, and geometries adapted to the anatomical environment. The utilization of biodegradable metals, including Mg, Fe, and Zn, is predominantly driven by their distinct degradation profiles and specific processing requirements. Their validation includes in vitro degradation tests in simulated media [51].

Osteochondral implants are designed to simultaneously repair articular cartilage and subchondral bone in complex injuries affecting both layers. Such injuries, prevalent in impact sports or degenerative diseases, compromise both the joint surface and the structural integrity of the bone, necessitating integrated solutions that restore joint function. The objective of this procedure is to restore lubrication, load distribution, and biomechanical continuity between cartilage and bone. It is imperative to impede the development of non-functional fibrocartilage while ensuring the maintenance of joint congruency and facilitating stable regeneration of both phases. The design typically incorporates layered configurations or gradient structures, exhibiting varied compositions, porosities, and rigidities. The superficial layer is optimized for chondrogenesis and shear stresses, while the basal zone must possess osteoconductive properties and compressive strength [12,49].

In the context of this particular application, the cost–benefit ratio is especially susceptible to economic influences due to its inherent economic implications. Metal lattices, as manufactured by PBF, facilitate mechanobiological coupling, thereby reducing the incidence of loosening and reinterventions. This, in turn, serves to offset the higher initial cost associated with custom design and manufacturing [46]. However, these architectures are sensitive to manufacturing defects, such as overmelting, surface irregularities, and dimensional deviations. If these defects are not controlled, they can degrade fatigue resistance and increase the risk of clinical failure. Consequently, allocating resources to process parameters, post-processing, and metrology tailored to lattices has been demonstrated to curtail adverse events and, in the long term, diminish cumulative hospital expenditures [50]. A secondary source of profitability is derived from the utilization of biodegradable metals, which obviate the necessity for surgical intervention and concurrently exhibit antibacterial properties in vitro and in vivo, thereby directly reducing infections [12,17]. Concurrently, the integration of lattice structures, particularly those exhibiting gradient topologies, has been demonstrated to enhance load distribution and stability within joints that are subjected to cyclic stresses. This integration has the capacity to prolong the functional lifespan of the implant and mitigate the necessity for revisions, thereby reinforcing the economic viability of the implant in cases where clinical necessity dictates [33]. Finally, implant-grade polymer alternatives with surface activation have demonstrated early osseointegration and higher bone–implant interface quality in vivo, which is expected to reduce early complications and resource consumption in follow-up despite higher material/processing costs [35]. A comprehensive evaluation of the available literature reveals that, while the initial purchase and manufacturing costs of these solutions exceed those of conventional implants, the subsequent reduction in reoperations, decreased infection management, and enhanced functional integration result in a cost–benefit ratio that favors their adoption in appropriately selected orthopedic indications [46,50].

### 4.2. Tissue Engineering

The second category of applications identified in the reviewed literature focuses on the field of tissue engineering, including structures and scaffolds specifically designed to promote the functional regeneration of biological tissues. In contrast to orthopedic implants, these solutions are not primarily designed to support structural loads; rather, they are intended to create microenvironments that stimulate cellular processes such as adhesion, proliferation, and differentiation. The objective of these applications is to identify properties such as interconnected porosity, nutrient permeability, cell compatibility, and controlled biodegradability. Furthermore, a significant proportion of the reviewed cases involved the integration of these systems with cells, growth factors, or biochemical signals, thereby facilitating the convergence of regenerative medicine strategies with advanced bio-inspired design methodologies [13].

In the domain of tissue engineering, the design scale of lattice structures exerts a pivotal influence on their functionality and the type of target tissue. At the macroscale, three-dimensional scaffolds with customized geometries are used to fill and support large tissue defects, maintain the anatomical shape of the area to be regenerated, and provide sufficient mechanical strength to prevent collapse during the regeneration process [14,27,29]. At this level, the cells are typically in the millimeter range, with controlled porosity that allows for tissue growth and progressive remodeling without compromising the structural integrity of the scaffold. At the microscale, applications are geared toward creating highly controlled microenvironments to guide cell adhesion, proliferation, and differentiation [21,32]. In this context, structures possess pores with a range of dimensions, from tens to a few hundred micrometers, exhibit high interconnectivity, and possess surfaces that are optimized at the nanometer or micrometer level to induce specific mechanical and biochemical signals. These scaffolds are indispensable in the fields of soft tissue regeneration, the development of biofabricated organs, and the recreation of complex tissue interfaces, including the osteochondral junction and the blood–brain barrier in in vitro models [39].

As is the case with orthopedic implants, applications in tissue engineering are subject to a structured methodological sequence. However, given the cellular and bioactive nature of these applications, the process may entail specific phases of material preparation, biological manipulation, and tissue evaluation. The process commences with the selection and formulation of the base material, which typically consists of a hydrogel or biopolymer capable of forming stable and biocompatible three-dimensional networks. This material can undergo chemical modification to enhance its reactivity, cell adhesion, or responsiveness to stimuli. Subsequently, the three-dimensional architecture of the scaffold is designed, with parameters such as pore size, interconnectivity, network density, and local stiffness being taken into consideration. This stage can be executed through the utilization of designated CAD tools or by establishing printing parameters based on the rheological characteristics of the bio-ink. In numerous instances, multilayer structures or functional gradients are employed to emulate the characteristic zoning patterns observed in natural tissues. The system’s manufacturing process utilizes high-resolution additive technologies. During this process, the incorporation of living cells into the material is possible, and the configuration of differentiated microenvironments according to the function of each region can be achieved. The resulting system must ensure the maintenance of cell viability and the retention of its physical properties for the duration necessary to induce tissue regeneration. The validation of implants involves a multifaceted approach encompassing physical-chemical testing, biocompatibility studies, and in vitro cell proliferation analysis, complemented by functional assessment in animal models. These evaluations encompass a comprehensive analysis of the extracellular matrix, gene expression, induced vascularization, and the integration of the regenerated tissue with the recipient environment [47].

The regeneration of articular cartilage is considered to be one of the most complex applications in this field. This complexity is attributed to the avascular nature of hyaline cartilage and its limited capacity for self-repair. These strategies target focal lesions that affect the joint surface and, in the absence of intervention, tend to evolve into degenerative processes such as osteoarthritis. The overarching objective of this research is to restore the structural and biofunctional integrity of hyaline cartilage. This is achieved by promoting the formation of an extracellular matrix that is rich in type II collagen and proteoglycans. The formation of this matrix is zonal, and it is similar to that of native tissue. In order to achieve this objective, it is imperative that the scaffolds in question possess the capacity to facilitate the process of chondrogenic differentiation. Moreover, it is essential that the scaffolds demonstrate the ability to maintain cellular viability and withstand moderate compressive loads during the process of tissue integration. These systems are distinguished by their microporous configurations, which facilitate nutrient diffusion and cell migration. Additionally, they exhibit intermediate stiffness, thereby simulating the physiological mechanical environment [47].

A practical case is described by Q. Li et al. [43], where hydrogel scaffolds composed of silk and gelatin were developed using DIW. These implants incorporated a stepped pore architecture designed to promote the zonal organization of hyaline cartilage. The structure was classified as wall-based, with a porous geometry that was optimized to facilitate nutrient diffusion and cell migration. The scaffolds were seeded with cell aggregates and subsequently implanted in a cartilage defect model in rabbits. After a period of 16 weeks, the group treated with the combination of scaffold and cell graft presented a continuous articular surface, organized regeneration of hyaline cartilage, and high expression of type II collagen, indicative of successful differentiation towards a cartilaginous lineage. From a mechanical perspective, a compressive modulus of 384 kPa was achieved, a value that approaches that observed in native tissue, rendering it suitable for withstanding moderate physiological loads during the tissue integration process. The observation that only the group treated with scaffolding and cell aggregates demonstrated regeneration with zonal organization and a compressive modulus approaching that of native tissue suggests that structured biofabrication alone is insufficient. The induction of functional cartilage regeneration is therefore contingent upon a synergistic combination with pre-organized cell units [43].

The objective of skin regeneration through tissue engineering is to treat complex skin injuries caused by burns, chronic ulcers, trauma, or surgery. The objective of these applications is to restore the skin’s barrier function, elasticity, and integration with neighboring tissues. This process aims to mitigate scarring and the risk of infection. The objective is to engineer a skin substitute that can safeguard the wound bed, stimulate angiogenesis, modulate inflammation, and facilitate the migration of keratinocytes and fibroblasts. Regeneration must encompass both the epidermis and dermis, respecting the stratified architecture and cell renewal of the skin tissue. The designs are predicated on multilayer matrices with high porosity and mechanical properties that allow for manipulation and suturing [21]. Their validation includes viability and proliferation tests in human dermal fibroblast cultures, mechanical characterization under traction or flexion, and in vivo tests in wound models [42].

In the domain of skin regeneration, numerous studies have been conducted on the development of hydrogel scaffolds through the utilization of additive technologies such as DIW and 3DBP. The most common formulations combine biopolymers such as GelMA, chitosan, gelatin, and aloe vera derivatives. These are selected for their biocompatibility, antimicrobial activity, and ability to form stable hydrated networks [13,27,42]. These structures are typically designed with 2.5D honeycomb geometries or cubic strut-based and wall-based configurations. They are designed with porosities of 70–85% and diameters greater than 200 μm, allowing for high permeability for gas exchange and cell migration. The compressibility modules obtained range between 10 and 60 kPa, a range comparable to that of dermal tissue, which reduces the risk of structural collapse in open wounds. In animal models, a significant reduction in healing time has been observed when compared to standard dressings, as well as faster and more organized re-epithelialization. A salient finding is that structures printed with regular geometry induce earlier vascularization and more homogeneous alignment of the regenerated tissue, in contrast to amorphous designs. Furthermore, studies incorporating functional nanoparticles or antimicrobial fillers have documented a reduction in local bacterial load and a lower incidence of postoperative infection [13,42].

Tissue engineering strategies are designed to promote muscle regeneration and to facilitate the healing of injuries to skeletal muscle caused by various factors, including trauma, surgical interventions, and degenerative diseases. Such injuries, particularly those involving volumetric loss, are incapable of regeneration through endogenous mechanisms and necessitate functional support to facilitate the reconstruction of contractile tissue. The objective of the treatment is to restore contractile function, fibrillar architecture, and vascularization by promoting myogenic differentiation and neuro-muscular integration. In this regard, systems are being developed that promote the fusion of myoblasts into multinucleated myofibers and cell alignment in the direction of the functional axis, thereby simulating the anisotropic pattern of muscle tissue. The induction of cell elongation and polarization is achieved through the implementation of aligned geometries, longitudinal channels, and surface topographies [29].

An exemplar of this category of applications is delineated in the article by Ogueri and Laurencin [14], wherein the authors expound on the utilization of aligned nanofiber scaffolds fabricated by electrospinning, employing biopolymers such as GelMA, chitosan, and gelatin, fortified with PCL fibers. The design of these systems incorporated cubic strut-based structures and directional orientation, a configuration that enabled them to emulate the mechanical anisotropy characteristic of skeletal muscle tissue. The architectures obtained exhibited elastic moduli ranging from 12 to 35 kPa and elongations at failure exceeding 50%, enabling them to withstand the longitudinal deformation characteristic of muscle movements without compromising structural stability. In vitro tests demonstrated excellent biocompatibility, accompanied by substantial proliferation of myoblasts and cell alignment parallel to the direction of the fibers. This alignment proved to be a critical factor in the expression of early muscle markers, indicating a more efficient myogenic differentiation process in comparison to isotropic or amorphous scaffolds. Furthermore, electrical contraction tests demonstrated that the scaffolds exhibited a propensity for impulse transmission and the formation of functionally active tissue, a critical property in the recovery of volumetric injuries [14].

Cell culture matrices function as three-dimensional platforms that simulate physiological microenvironments, thereby enabling the study of processes such as cell adhesion, proliferation, migration, and differentiation. These cells are not intended for direct implantation; rather, they are utilized in fundamental research, pharmacology, and biomaterial validation. The objective of this study is to provide adequate physical and chemical support for the development of stable three-dimensional cultures, thereby overcoming the limitations of conventional two-dimensional cultures. These matrices possess micro- or nanoporous architectures, which are characterized by elevated specific surface area and connectivity. This structural attribute enables the efficient transportation of nutrients and gases, thereby facilitating the establishment of biochemical gradients within the matrix. In certain designs, topographies or levels of roughness are incorporated to enhance cell adhesion or to emulate tissue heterogeneity. The experimental validation process involves conducting in vitro studies of viability, morphology, and cell distribution. These studies utilize confocal microscopy, fluorescent staining, and proliferation quantification to obtain quantitative data. These matrices also permit the observation of dynamic phenomena, such as cell migration or extracellular matrix secretion, and they can be used for prolonged cultures or multicellular co-cultures [18].

Tissue bioprinting matrices are 3D platforms developed for the purpose of printing together with living cells, growth factors, or bioactive components. In contrast to conventional culture matrices, these structures are designed to be integrated into biological manufacturing processes, with the objective of generating functional tissues under controlled conditions. The objective of the present study is to provide immediate, biocompatible support that promotes the spatial arrangement, viability, and differentiation of the printed cellular components. To achieve this objective, it is imperative to exercise control over cross-linking, ensure initial mechanical stability, and facilitate biochemical interaction, while concurrently permitting subsequent tissue maturation. These bio-inks are formulated with natural or synthetic hydrogels, meticulously adjusted for viscosity and rheological behavior. The architectural design of these platforms can incorporate channels, gradients, or differentiated zones, depending on the cell load or functionalization of the tissue [21]. This design allows for the replication of tissues with functional heterogeneity.

An exemplary study is the article developed by Jin et al. [15], where scaffolds based on GelMA functionalized with bioactive nanoparticles were designed and manufactured using direct extrusion techniques. The obtained structure corresponded to a cubic strut-based configuration, optimized to maintain dimensional integrity during the printing process and subsequent cell culture. Over the course of a week-long viability test, rates surpassing 90% were observed, indicating a high degree of compatibility between the functionalized hydrogel and the seeded cells. Mechanically, the scaffolds exhibited a stiffness of 20 kPa, a value that falls within the optimal range for applications in soft tissues such as dermis, mucosa, or loose connective tissue. Moreover, the incorporation of these nanoparticles endowed the system with antimicrobial properties and a sensitive response to external stimuli, without compromising print resolution or the system’s overall biocompatibility. This methodology enables the incorporation of advanced functionalities without compromising structural stability or cell support capacity, thereby offering a pragmatic alternative to conventional bioinks that prioritize viability but exhibit limited active response [15].

Finally, matrices for direct organoid printing represent an emerging application aimed at creating self-regenerating three-dimensional models that simulate functional organ units. These matrices facilitate the precise deposition of stem or progenitor cells, thereby promoting their self-organization and maturation into complex structures with partial or complete functionality. The objective of this study is to provide a bioactive environment that favors cell differentiation and assembly without interfering with self-organization processes. Furthermore, it is imperative that these structures facilitate the efficient diffusion of nutrients, gases, and signals. The process of validation encompasses the establishment of stable three-dimensional structures, the expression of differentiation markers, and the functional evaluation of the units generated. These units may be evaluated for their capacity to secrete metabolites, form lumen, or respond to stimuli. Immunofluorescence, confocal microscopy, and three-dimensional analysis techniques are applied, and in vivo models are used to evaluate the integration or functionality of the implanted organoid [29,32].

Within the domain of tissue engineering, the cost–benefit ratio is determined by the initial investment required for advanced biomaterials and biofabrication techniques, as compared to the clinical and economic gains they generate in the medium and long term. The application of lattice scaffolds produced by three-dimensional (3D) printing with functionalized hydrogels and customized bioinks increases material and process costs. However, these expenses are offset by the ability to adapt the microarchitecture at the cellular level, which improves cell adhesion and proliferation and promotes faster and more efficient regeneration [39,40]. This acceleration of regenerative processes has been shown to result in substantial reductions in hospital stays and a decreased reliance on complementary treatments, leading to significant savings for the healthcare system [41]. Conversely, the development of scaffolds with tuned porosity gradients and mechanical properties has been shown to reduce the risk of tissue necrosis, rejection, or mechanical failure, thereby minimizing the costs associated with postoperative complications [42]. In this regard, while advanced printing bioprocesses necessitate higher initial expenditures, the clinical outcomes—including enhanced stability, functional integration, and reduced intervention frequency—demonstrate a cost–benefit ratio that is favorable to the patient [43].

### 4.3. Implantable Devices

The third category of applications, as identified in the extant literature, pertains to the development of implantable devices whose function does not lie in replacing damaged tissue or directly guiding its regeneration. Rather, these devices are designed to perform a specific therapeutic, sensory, or functional action within the body. In contrast to the two preceding groups, these devices are distinguished by their technological approach and their active interaction with the biological environment, whether by releasing compounds, detecting physiological signals, or acting as temporary supports in anatomical structures. In these applications, the structural design does not seek to emulate the architecture of a specific tissue; rather, it seeks to optimize the functional response of the implanted system. Additive technologies facilitate the realization of these functions by manufacturing miniaturized, multi-component, or multi-material devices with degrees of complexity that are unattainable by conventional techniques [30].

In the context of this particular application, it is imperative to differentiate between the scale at which the device operates. At the macroscale, these devices include elements that must withstand significant mechanical loads or maintain a stable geometry over long periods, such as internal fixation systems [30]. In such cases, the unit cells are typically millimeter-sized, with designs engineered to resist fatigue, reduce stress shielding, and adapt anatomically to the implantation site. These cells are also engineered to maintain channels that facilitate vascularization and nutrient transport. Conversely, at the microscale, implantable devices prioritize direct interaction with soft tissues or body fluids, exhibiting structures with pores and channels ranging from tens to a few hundred micrometers [20,26,28]. This approach has been successfully implemented in a variety of medical devices, including vascular stents, scaffolds for controlled drug release, devices for nerve regeneration guidance, and sensory microimplants. The microarchitecture is designed to control phenomena such as fluid flow, specific cell adhesion, or modulation of the local immune response, with particular attention to biocompatibility and minimization of fibrous tissue or thrombus formation [26,28].

Despite the fact that the applications encompassed in this group do not aspire to a direct replacement of tissues, they exhibit a congruent approach to development that integrates functional design, bioactive integration, and specific validation according to the device’s function. In contrast to orthopedic implants or cellular structures, these implantable systems are required to meet specific functional criteria. These criteria may include the controlled release of molecules, the detection of internal signals, or the temporary maintenance of a physiological function. The process commences with the precise delineation of the therapeutic or technological function that the device is required to fulfill. The selection of the base material, which may be biodegradable or stable, is made based on these factors, and its key properties are defined, including its diffusion rate, response to stimuli, stiffness, conductivity, and behavior in biological fluids. The three-dimensional geometry of the system is then designed, with the objective of adapting it to the anatomical environment in which it will be implanted and the physiological dynamics of the surrounding tissue. At this stage, structures such as channels, chambers, micro-depots, anchoring zones, or flexible sections are integrated using digital modeling and functional simulation tools. In active devices, this phase may include the incorporation of sensitive elements or biocompatible electronic components. The manufacturing process utilizes additive technologies, enabling the requisite precision in the microstructure, the assembly of multiple materials, and the creation of internal cavities. In certain instances, printing techniques are integrated with functional coatings or controlled encapsulation processes. The objective is to create a device that maintains its functionality under real physiological conditions. Finally, system validation encompasses a series of evaluations, including in vitro functional performance testing (release, response, sensitivity), structural characterization, biocompatibility testing, and, when deemed necessary, in vivo studies. These studies are conducted to assess the integration, performance, and tissue response of the implant during the designated residence time.

Among these applications, devices for controlled drug release represent one of the most developed areas in AM applied to localized therapies. The primary function of these devices is to administer active pharmaceutical agents directly into the target physiological environment, thereby ensuring the maintenance of sustained therapeutic concentrations and the minimization of systemic adverse effects. These systems offer an effective alternative to traditional routes of administration, especially in treatments that require prolonged release, localized action, or avoidance of first-pass hepatic metabolism. The objective is to ensure the stability of drug levels at the site of action by adjusting release kinetics through the control of porosity, material degradation, and active ingredient formulation. In certain instances, the devices are engineered to respond to specific stimuli, such as pH, temperature, or pressure. This enables active or modulated release. From a structural perspective, these systems incorporate porous architectures, internal cavities, or diffusion channels, which are configured to house and release the active ingredient gradually. The geometry of the device functions as a diffusion barrier, and the release rate is modulated by adjusting the thickness of the walls, the exposed surface area, and the degradation rate of the base material [21]. The experimental validation process entails in vitro release studies in simulated media, kinetic analyses, active ingredient stability tests, and biocompatibility tests. In more advanced stages, in vivo studies are carried out to evaluate tissue response, therapeutic efficacy, and degradation behavior [32].

In this field, various studies have employed cubic strut-based and TPMS wall-based structures manufactured using additive technologies such as FFF, DLP, and DIW, using biodegradable polymers such as PLA, PCL, and PLGA, as well as natural biopolymers such as alginate, collagen, or chitosan [16,20,22,27,28,30,45]. These configurations enable precise modulation of porosity, pore size, and strut or wall thickness—parameters that are critical in the modulation of release kinetics. In several cases, porosities greater than 70% were achieved, with material degradation rates adjustable between 2 and 12 weeks, allowing sustained delivery of active compounds to target tissues [16]. For instance, sequential release profiles with distinct phases during the first 24 h, followed by stable release for more than 10 days, were documented. These profiles were found to depend on the geometry and degree of cross-linking of the system. The structures were also designed with specific internal zones—microdeposits and diffusion channels—digitally integrated into the CAD model and reproduced with high fidelity, achieving coefficients of variation of less than 5% in the daily release rate [20]. In preclinical models, these devices achieved localized therapeutic concentrations without systemic peaks or observable toxicity. In contrast to conventional coating or encapsulation methodologies, this structural approach enables the customization of pharmacokinetic function without altering the formulation of the active ingredient, thereby facilitating its integration into combination therapies or post-surgical extended-release treatments [22,30].

Implantable sensory devices represent a nascent field within the biomedical sector, with a focus on in situ monitoring of physiological or mechanical parameters through the utilization of advanced structural technologies. Their incorporation into the biological environment enables the acquisition of data concerning pathological processes, treatment evolution, and functional tissue dynamics without the necessity of external intervention. This development presents novel opportunities in the domains of personalized medicine, postoperative monitoring, and smart therapies. The primary function of these devices is to detect changes in variables such as pressure, tension, humidity, pH, or electrical activity and translate them into quantifiable signals without compromising the biocompatibility or stability of the system [19]. In certain instances, these devices are integrated with active release or therapeutic feedback systems, thereby transforming into multifunctional platforms capable of simultaneous diagnosis and action. These devices are composed of functionalized polymers, piezoelectric composites, or two-dimensional materials such as MXenes, which are capable of converting mechanical stimuli into electrical signals. The structures are printed with three-dimensional configurations that maximize sensitivity, anatomical adaptability, and stability against dynamic cycles [19].

Accordingly, the investigation conducted by Wei et al. [19] examines the evolution of implantable sensors derived from functional materials, including MXenes and piezoelectric polymers. The fabrication of these platforms involved the implementation of 4D printing technologies, which facilitate the integration of shape memory or responsiveness to external stimuli. The structural design of these platforms is characterized by a cubic strut-based lattice framework, featuring high levels of porous connectivity. This structural configuration ensures superior anatomical adaptability, which is crucial for tissues subjected to dynamic cycles, such as the heart or muscles. It also improves sensor sensitivity by promoting localized deformation over active areas of the device. The adjusted elastic modulus of the polymers utilized ranged from 10 to 50 kPa, enabling the device to adapt without causing tissue damage while maintaining its structural integrity in repeated cycles of compression and relaxation. Furthermore, the study documents that, after undergoing more than 105 mechanical loading cycles under simulated conditions, there was no functional fatigue or loss of sensory capacity, thereby supporting its use in post-surgical monitoring or smart therapies. In contrast to external or superficially coupled sensors, these systems enable continuous real-time monitoring of the internal physiological environment, thereby opening new avenues for personalized medicine and integrated therapeutic feedback systems [19].

Another notable application is implantable temporary stents, which are designed to maintain the patency of anatomical conduits for a limited period, usually while the surrounding tissue heals or regenerates. These devices are frequently utilized in various medical contexts, including cardiovascular, esophageal, respiratory, and urinary systems. In these instances, temporary mechanical support facilitates the restoration of physiological function. These devices are designed to prevent the collapse of tubular structures, restore internal flow, or facilitate the temporary drainage of secretions or fluids. In order to achieve this objective, it is essential to ensure that the initial strength is adequate, the degradation is predictable, and the biocompatibility is high, while avoiding the induction of inflammation or adverse reactions. Experimental validation encompasses in vitro studies in simulated media, wherein the degradation profile, mechanical evolution under cyclic loading, and response to degradation products are analyzed. In vivo tests are also performed to evaluate stent integration, tissue response, and functional capacity during the expected period.

A notable example is described by Sezer et al. [30], who developed a biodegradable tracheal device manufactured from Mg alloys using SLM. The structural design entailed a helical tubular strut-based configuration, meticulously engineered to ensure airway patency while facilitating functional recovery of the respiratory tissue. The stent exhibited adequate initial mechanical strength to withstand tracheal collapse forces, and its degradation rate was sufficiently rapid that complete resorption occurred after restoration of lung function. Consequently, the need for a second surgical intervention for removal was avoided. In vivo testing in animal models demonstrated progressive degradation without chronic inflammation, accompanied by complete regeneration of the respiratory epithelium after several weeks. In terms of biocompatibility, the results of the cellular response assays were favorable, and the histological analyses showed no evidence of fibrosis or hyperplasia in the surrounding tissue [30].

In the context of implantable devices, cost–benefit analysis entails a balanced assessment of technological complexity and manufacturing costs, juxtaposed against the clinical safety and long-term functionality promised by these solutions. AM facilitates the development of internal microstructures and customized geometries that are not possible with conventional methods, which increases design and manufacturing costs. However, the potential for integrating additional functions into a singular device—such as controlled drug release, electrical conductivity for tissue stimulation, or bioactive surfaces to enhance integration—significantly reduces the necessity for external therapies and additional invasive procedures, resulting in a favorable economic impact [44]. Furthermore, the integration of advanced biomaterials and lattice structures in the customization of pacemakers, stents, valves, and implantable sensors has been demonstrated to reduce complications such as thrombosis, rejection, and mechanical failure. This, in turn, results in a reduction in hospital admissions and cumulative savings in clinical follow-up [45]. The economic viability of the device is influenced by its superior durability and the reduced risk of early replacement, as these factors extend the device’s useful life and eliminate costs associated with replacement surgery [46]. In this regard, although implantable devices manufactured using additive technologies involve a high initial cost, their clinical benefits—greater safety, advanced functionality, and fewer complications—consolidate a favorable cost–benefit ratio compared to conventional alternatives [47].

## 5. Conclusions

This article presents a comprehensive examination of the current state of the art in the design, manufacture, characterization, and application of lattice structures using additive technologies in the biomedical sector. A systematic literature search and a selection methodology based on the PRISMA approach were employed to analyze forty relevant studies ranging from experimental developments to early clinical applications. This review has facilitated the identification of design trends, predominant technological approaches, materials used, and common validation strategies. Furthermore, it has enabled the establishment of a functional classification of applications into three main groups: orthopedic implants and bone regeneration, tissue engineering, and implantable devices.

The primary conclusions derived from the analysis are presented below, followed by the priority lines of development identified to advance the clinical consolidation of these technologies.

- Lattice structures, developed using AM technologies, have consolidated their role as an effective design strategy in the creation of multifunctional biomedical devices and systems. Their capacity to modulate mechanical, topological, and biological properties renders them a versatile instrument for diverse clinical applications, ranging from tissue regeneration to controlled drug delivery.
- One of the primary strengths identified pertains to the capacity of these structures to function across diverse functional scales, ranging from macro-scale prostheses with customized geometries to cellular microenvironments characterized by biochemical gradients and hierarchical architectures. This adaptability renders it capable of responding to heterogeneous clinical needs with solutions that simultaneously integrate mechanical support, cellular interaction, and degradation control.
- In the domain of orthopedics, lattice has facilitated the development of implants and prostheses with enhanced bone integration by reproducing the porosity, rigidity, and trabecular architecture of natural bone. Furthermore, it has enabled the conceptualization of provisional biodegradable systems that offer structural reinforcement during the regenerative process and are gradually reabsorbed in a regulated manner, obviating the necessity for surgical extraction.
- In the field of tissue engineering, lattice structures have undergone a remarkable evolution, evolving from simple scaffolds to functional microenvironments with the ability to regulate complex biological processes. Their role is particularly important in soft tissue regeneration, in matrices for three-dimensional cell culture, and in bio-printed platforms that allow cells, biomolecules, and factors to be positioned in a precise and controlled manner.

In view of the aforementioned conclusions, as well as the identified limitations and gaps within the extant literature, the primary lines of development that should guide future research in this domain are outlined below:

- The modeling of structures with high unit cell density and complex geometries remains a computationally expensive process. There is an urgent need for specific CAD tools and more efficient simulation software, especially for structures with functional gradients, such as those used in bone prostheses and drug delivery systems.
- The formation of internal defects and geometric deviations during manufacturing remains a critical obstacle to ensuring clinical reproducibility. The enhancement of inspection methodologies, the delineation of resilient process parameters, and the formulation of distinct quality standards for these structures are imperative steps toward regulation and clinical approval.
- Numerous studies have demonstrated encouraging results in computational models or in vitro tests; however, a paucity of biological validation under real physiological conditions is evident in the extant literature. A transition to systematic in vivo studies is imperative to evaluate tissue integration, biodegradability, immune response, and mechanical functionality over the medium and long term.
- The use of advanced architectures with unconventional properties, such as auxetic structures or optimized TPMS, has begun to attract interest due to their ability to integrate functional response, anatomical adaptation, and simultaneous mechanical control. The integration of these topologies with stimulus-sensitive materials and multiscale topological optimization algorithms is anticipated to facilitate the development of bioactive, personalized, and adaptive devices.
- The potential for the development of customized devices, tailored to the individual patient’s radiological data (DICOM), signifies a paradigm shift towards direct integration between clinical diagnosis and in situ manufacturing. The establishment of hospital platforms equipped with certified printers, validated materials, and digital workflows would facilitate the production of customized prostheses, surgical guides, or regenerative scaffolds within clinically viable timeframes.

## Figures and Tables

**Table 1:** Inclusion and exclusion criteria used in the search.

Inclusion/Exclusion Criteria	Feature
Publication period	From 1 January 2020 to 1 June 2025
Search date	1 June 2025
Type of study	Articles: journals, reviews, and conference proceedings
Keywords and synonyms	See Figure 2
Information sources	WoS, Scopus
Databases in WoS	Option ‘All databases’
Language of publication	English
Publication mode	Exclusively open access
Quality criteria required for each publication	Peer-reviewed articles included in WoS. Journal articles limited to Q1–Q2
Quality criteria reviewed for each publication	Reviewed by journal impact factor and journal citations reports

**Table 2:** Main analysis of data from chosen search documents. The symbol ‘X’ indicates that the documents considered refer to or contain content relevant to the specified category.

Documents	Number of References	Publication Type	State of the Art	Process Optimization	Experiments Design	Country	Publication Year
[6]	519	Q1	X			Italy	2021
[12]	166	Q1	X			United States	2022
[13]	86	Q1			X	Finlandia	2021
[14]	93	Q1	X			United States	2020
[15]	97	Q1			X	China	2021
[16]	86	Q1			X	Netherlands	2020
[17]	61	Q2	X	X		Poland	2021
[18]	166	Q1		X	X	Germany	2021
[19]	145	Q1			X	Ireland	2022
[20]	121	Q1			X	Belgium	2022
[21]	251	Q1	X			Pakistan	2022
[22]	70	Q2	X	X		Netherlands	2022
[23]	404	Q1	X			China	2020
[24]	70	Q1	X			United States	2023
[25]	64	Q2	X			Spain	2020
[26]	109	Q1	X			China	2021
[27]	67	Q1	X	X		China	2020
[28]	146	Q1	X	X		China	2020
[29]	78	Q1	X			Switzerland	2020
[30]	139	Q1	X			Qatar	2021
[31]	120	Q1		X	X	United States	2021
[32]	168	Q1	X			Germany	2020
[33]	107	Q2	X			England	2022
[34]	89	Q1			X	China	2023
[35]	76	Q2	X			Italia	2023
[36]	170	Q1	X	X		China	2020
[37]	102	Q1			X	China	2022
[38]	98	Q1	X			China	2023
[39]	87	Q1			X	China	2021
[40]	149	Q1		X	X	China	2023
[41]	93	Q1			X	China	2022
[42]	76	Q2			X	China	2023
[43]	197	Q1			X	China	2021
[44]	169	Q2	X			England	2020
[45]	74	Q1	X			China	2023
[46]	60	Q2	X			China	2020
[47]	71	Q1			X	Switzerland	2022
[48]	92	Q1		X		Netherlands	2020
[49]	69	Q1	X			Australia	2023
[50]	155	Q1	X	X		United Arab Emirates	2020

**Table 3:** AM categories according to international standard ISO/ASTM 52900:2021.

Category		Technologies	Principle	Characteristics
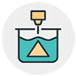	Photopolymerization in tank	SLA DLP CLIP	A liquid photopolymer solidifies layer by layer through controlled exposure to a light source.	High resolution and manufacturing speed. Limited to photosensitive resins. High material cost.
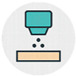	Material jetting	Polyjet MJP	Microdroplets of photopolymer material are selectively deposited and cured using UV light.	Allows multi-material printing. High surface quality. Materials with low mechanical resistance.
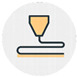	Material extrusion	FFF ADAM	A thermoplastic filament is melted and extruded through a nozzle that moves according to the design.	Economical and versatile technology. Supports multi-material. Low resolution and limited surface quality.
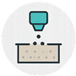	Bonding agent jet	BJ MJF SPJ	A liquid binding agent is selectively deposited on a bed of powder to bind the particles together.	Wide range of materials. Allows color printing. Requires infiltration and post-processing. High porosity.
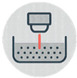	Powder bed fusion	SLM EBM SLS DMLS	A laser or electron beam selectively melts layers of powder in a bed to consolidate them.	High precision and density of parts. Suitable for metals and technical polymers. Requires thermal management.
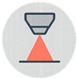	Direct energy deposition	LENS EBAM LMD WAAM	Powder or filament material is deposited with the application of energy for melting and consolidation.	High deposition rate. Suitable for repair and functional grading. Requires post-processing for finishing.
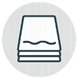	Sheet lamination	LOM UAM	Thin sheets of material are cut and stacked layer by layer by adhesion or welding.	Low cost and good surface quality. Limited to certain materials. Restrictions on complex geometries.

**Table 4:** Comparison between the characteristics of the most common FA technologies found in the literature. ($ Low cost $$ Medium cost $$$ High cost $$$$ Very high cost).

Technologies	Strengths	Limitations	Resolution	Cost
DLP	Excellent resolution. High printing speed. Good surface quality.	Low mechanical properties. Limited range of materials. Need for post-processing.	25–50 µm	$$
FFF	Low cost. Greater thermal conductivity of parts. Ease of use. Wide range of materials.	Low resolution. Pronounced anisotropic mechanical properties. Need for supports.	250–370 µm	$
BJ	Low cost. Very fast process. Full-color printing. No supports. High work volume.	Low mechanical properties. Rough surface. Need for curing and post-processing.	50–200 µm	$
SLM	Excellent mechanical properties. Complex geometries. High density.	High cost. Rough surface. Low resolution. Need for rigorous thermal management.	80–250 µm	$$$
EBM	Excellent mechanical properties. Complex geometries. No supports. Good resolution.	High cost. Rough surface. Requires thermal management.	50–100 µm	$$$$
SLS	High printing speed. No supports. Remarkable mechanical properties. Efficient resource consumption.	High cost Rough surface. Limited construction volume.	76–100 µm	$$$
LENS	Excellent mechanical properties. Possibility of integrating various materials. High printing speed.	Low resolution. Rough surface. Residual stresses. Simple geometries.	250 µm	$$$
DIW	Low cost. Low temperatures. Flexible manufacturing. Good resolution.	Need for supports. Low mechanical properties.	<250 µm	$
3DBP	Wide variety of hydrogels and bioinks. Good resolution. Innovative applications.	Limited by cell viability. Low mechanical properties. High complexity.	<250 µm	$$$$

**Table 5:** Comparison between the characteristics of the most common fabrication materials found in the literature.

Category	AM Technology Associated		Strengths	Limitations
Metals	SLM EBM SLS	LENS 4DP	High mechanical strength. High thermal stability. Corrosion resistance.	High cost. Low biodegradability. Risk of toxicity.
Polymers	DLP SLS FFF	BJ DIW 3DBP	Biocompatibility. Biodegradable. Adjustable mechanical properties. Adaptability through composition.	Low thermal stability. Need for post-treatment. Excessive elasticity modulus.
Ceramics	BJ DIW 4DP		High thermal stability. Bioactivity. Corrosion resistance.	Fragility. Difficult to process. Requires post-treatment.
Composites	SLS FFF BJ	DIW 3DBP 4DP	Adjustable mechanical properties. Compatibility with reinforcements. Adaptability through composition. Corrosion resistant.	High cost. Difficult to process.

**Table 6:** Mechanical properties, porosity, and density of different human body structures or tissues.

Anatomical Structure	Porosity (%)	Density (g/cm^3^)	Yield Strength (MPa)	Compressive Strength (MPa)	Young’s Modulus (GPa)
Cortical bone	3–12	1.85 ± 0.06	50–150	130–193	3–30
Trabecular bone	50–90	0.3 ± 1	10–20	4–12	0.02–0.5
Articular cartilage	70–85	1.1 ± 0.05	0.3–0.6	0.5–1	0.0003–0.001
Dental enamel	<1	2.9 ± 0.1	-	300	70–100
Dentin	15–20	2.1 ± 0.1	80–100	250	12–20
Ligament	55–70	1.15 ± 0.05	50–100	-	0.15–0.6
Tendon	60–70	1.2 ± 0.05	70–120	-	1–2

**Table 7:** Mechanical properties, biocompatibility, and corrosion resistance of metals manufactured using AM most commonly used in lattice structures in the biomedical sector (* Bad ** Regular *** Good **** Best).

Metal	Density (g/cm^3^)	Yield Strength (MPa)	Compressive Strength (MPa)	Young’s Modulus (GPa)	Biocompatibility	Corrosion Resistance
Ti	4.5 ± 0.1	275–480	230–1241	90–120	****	***
Ti alloys	4.35 ± 0.15	800–1000	750–1110	110–120	****	***
Co alloys	8.3 ± 0.45	450–600	600–1000	210–230	***	***
316L Steel	7.9 ± 0.05	500–600	190	193	***	***
Ta	16.6 ± 0.05	230	200–300	186–191	***	****
Ni alloys	6.45 ± 0.15	195–690	400–800	75	***	***
Mg	1.74 ± 0.08	20–50	100–200	40–45	***	*
Mg alloys	1.90 ± 0.1	130–180	100–200	35–50	****	**
Fe	7.87 ± 0.1	200	200–300	210	***	***
Zn	7.14 ± 0.1	10–30	20–30	70–100	***	***
Zn alloys	6.85 ± 0.15	100–160	150–230	80–105	***	***

**Table 8:** Mechanical properties, biocompatibility, and corrosion resistance of polymers manufactured using AM most commonly used in lattice structures in the biomedical sector (* Bad ** Regular *** Good **** Best).

Polymer	Density (g/cm^3^)	Yield Strength (MPa)	Compressive Strength (MPa)	Young’s Modulus (GPa)	Biocompatibility	Corrosion Resistance
PEEK	1.3 ± 0.1	87–95	118–130	3–4	****	***
PA12	1.01 ± 0.08	45–55	65	1.4–1.85	**	**
PLA	1.24 ± 0.1	50–70	60–70	3.5–3.8	***	*
PCL	1.1 ± 0.1	23	39	0.3–0.4	***	*
PLGA	1.34 ± 0.07	45	41–55	1.4–2.8	***	**
GelMA	1.05 ± 0.08	-	-	-	****	*
Alginate	1.0 ± 0.05	-	-	-	****	*
Collagen	1.0 ± 0.05	-	-	-	****	*
Chitosan	0.9 ± 0.1	-	-	-	***	**

**Table 9:** Mechanical properties, biocompatibility, and corrosion resistance of ceramics manufactured using AM most commonly used in lattice structures in the biomedical sector (** Regular *** Good **** Best).

Polymer	Density (g/cm^3^)	Yield Strength (MPa)	Compressive Strength (MPa)	Young’s Modulus (GPa)	Biocompatibility	Corrosion Resistance
HAp	3.16 ± 0.06	80	120–150	80	****	***
β-TCP	3.07 ± 0.04	50	90–100	45	***	**
Al_2_O_3_	3.9 ± 0.1	370–380	2000–4000	370	***	****
Y-TZP	6.05 ± 0.1	900–1200	1500–2000	210	***	****
Bioglass 45S5	2.7 ± 0.15	40–60	500	30–35	****	**

**Table 10:** Mechanical properties, biocompatibility, and corrosion resistance of composites manufactured using AM most commonly used in lattice structures in the biomedical sector (** Regular *** Good **** Best).

Composite	Density (g/cm^3^)	Yield Strength (MPa)	Compressive Strength (MPa)	Young’s Modulus (GPa)	Biocompatibility	Corrosion Resistance
PLA/HA	1.63 ± 0.4	20–40	5–40	2.3–4	***	**
PCL/β-TCP	1.3 ± 0.1	15–30	3–20	0.3–1	****	***
PLA/fibra de carbono	1.3 ± 0.2	50–60	10–20	5–10	**	***
PU/fibras	1.2 ± 0.1	35–50	30–80	0.1–1	***	***

**Table 11:** Comparison of the most important properties of lattice 2.5D structures (* Bad ** Regular *** Good **** Best).

2.5D Structure	Yield Strength	Compressive Strength	Young’s Modulus	Fatigue Resistant	Energy Absorption	Anatomical Adaptability	Specific Surface	Permeability	Ease of Manufacturing
Honeycomb	**	***	***	*	**	*	*	*	****
Auxetic	**	**	**	***	****	****	**	*	***

**Table 12:** Mathematical expressions of TPMS structures found in the literature.

TPMS Structure	Mathematical Expression		
Schwarz	$\varphi \left(x,y,z\right)=\cos\left(w_{x}X\right)+\cos\left(w_{y}Y\right)+\cos\left(w_{z}Z\right)=C$	(3)	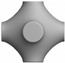
Diamond	$\varphi \left(x,y,z\right)=\cos\left(w_{x}X\right)\cdot \cos\left(w_{y}Y\right)\cdot \cos\left(w_{z}Z\right)-\sin\left(w_{x}X\right)\cdot \sin\left(w_{y}Y\right)\cdot \sin\left(w_{z}Z\right)=C$	(4)	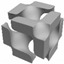
Gyroid	$\varphi \left(x,y,z\right)=\sin\left(w_{x}X\right)\cdot \cos\left(w_{y}Y\right)+\sin\left(w_{y}Y\right)\cdot \cos\left(w_{z}Z\right)+\sin\left(w_{z}Z\right)\cdot \cos\left(w_{x}X\right)=C$	(5)	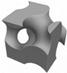
I-WP	$\varphi \left(x,y,z\right)=2\cdot \left[\cos\left(w_{x}X\right)\cdot \cos\left(w_{y}Y\right)+\cos\left(w_{z}Z\right)\cdot \cos\left(w_{x}X\right)+\cos\left(w_{y}Y\right)\cdot \cos\left(w_{z}Z\right)\right]\;\;\;\;\;\;\;\;\;\;\;\;\;\;\;\;\;\;\;\;\;\;\;\;\;\;\;\;\;\;\;\;\;\;-\left[\cos\left(2w_{x}X\right)+\cos\left(2w_{y}Y\right)+\cos\left({2w}_{z}Z\right)\right]=C$	(6)	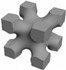

**Table 13:** Comparison of the most important properties of Lattice 3D strut-based structures (* Bad ** Regular *** Good **** Best).

Strut-Based Structure	Yield Strength	Compressive Strength	Young’s Modulus	Fatigue Resistant	Energy Absorption	Anatomical Adaptability	Specific Surface	Permeability	Ease of Manufacturing
Cubic	*	*	*	*	**	*	*	***	****
BCC	**	**	**	**	**	**	**	***	****
FCC	***	***	***	***	**	**	**	**	***
Octet-truss	****	****	****	***	**	**	**	***	**
Diamond	****	****	****	***	**	***	***	**	**
Auxetic	**	**	**	***	****	****	**	***	**
TPMS Schwarz	***	****	***	**	**	***	****	***	**
TPMS diamond	****	****	****	***	**	***	****	**	**
TPMS gyroid	***	***	***	***	***	***	****	***	*
TPMS I-WP	***	***	***	***	**	***	****	**	*
Voronoi	***	***	***	***	***	****	***	***	*

**Table 14:** Comparison of the most important properties of Lattice 3D wall-based structures (* Bad ** Regular *** Good **** Best).

Wall-Based Structure	Yield Strength	Compressive Strength	Young’s Modulus	Fatigue Resistant	Energy Absorption	Anatomical Adaptability	Specific Surface	Permeability	Ease of Manufacturing
Cubic	**	**	**	**	**	**	**	*	****
BCC	**	**	**	**	***	**	**	*	****
FCC	***	***	***	***	***	**	**	**	***
Octet-truss	****	****	****	****	***	**	**	**	***
Diamond	****	****	***	***	***	***	***	**	***
TPMS Schwarz	****	***	****	****	***	****	****	*	**
TPMS diamond	****	****	****	****	****	****	****	***	**
TPMS gyroid	***	***	****	***	***	***	****	**	**
TPMS I-WP	***	**	***	***	***	****	****	**	**
Voronoi	***	***	**	***	***	****	****	**	**

**Table 15:** Applications in the biomedical sector of lattice structures manufactured using additive technologies found in the literature.

Biomedical Application		AM Technologies Most Used	Fabrication Materials Most Used	Lattice Structures Most Used	Most Sought-After Properties	References
Orthopedic implants and bone regeneration	Hip prostheses	SLM, EBM, SLS, LENS	Ta Ti, Co alloys	Strut-based cubic, diamond, tetrahedron, auxetic, TPMS gyroid and Voronoi	High mechanical properties, biocompatibility, fatigue resistance, osseointegration and anatomical adaptability	[12,22,24,25,31,33,48]
	Joint prostheses	SLM, EBM	Ti, Co alloys	Strut-based cubic, diamond, octet-truss, octahedron and truncated octahedron	Biocompatibility, fatigue resistance, osseointegration and anatomical adaptability	[12,24,25,31,35]
	Mandibular prostheses	SLM, EBM	Ti alloys	Strut-based diamond and octet-truss	High mechanical properties, biocompatibility, lightweight, osseointegration, anatomical adaptability and optimized functionality	[12,17,25,45]
	Cranio-maxillofacial implants	SLM, EBM	Ti, Ta, 316L steel Ni, Ti alloys 316L steel	Strut-based diamond, TPMS gyroid and Voronoi Wall-based Voronoi	High mechanical properties, biocompatibility, lightweight, osseointegration, permeability and anatomical adaptability	[12,17,22,24,25,33,38]
	Dental implants	SLM, EBM	Ti alloys	Strut-based BBC and diamond	High mechanical properties, biocompatibility, fatigue resistance, load cushioning, anatomical adaptability	[12,17,24,25,31]
	Vertebral structures	SLM, EBM, SLS, LENS	Ti alloys PEEK	Strut-based cubic, BBC, FCC, diamond, octet-truss, TPMS gyroid	Biocompatibility, high axial load capacity, osseointegration, anatomical adaptability	[6,12,25,33]
Orthopedic implants and bone regeneration	Correction of bone defects	DLP, SLA, SLM, BJ, FFF, DIW	Ti, Ta, 316L steel Ti alloys Fe, Mg, Zn alloys PLA, PCL, PLGA, PEEK HAp, ZrO_2_, β-TCP, Bioglass	2.5D Honeycomb Strut-based BCC, FCC, diamond, octet-truss, TPMS gyroid, schwarz, Voronoi Wall-based TPMS gyroid, diamond	Biocompatibility, biodegradability, high interconnected porosity, osteointegration, anatomical adaptability	[18,23,30,31,32,34,36,37,41,42,45,47,51]
	Bone regeneration under load	DLP, SLM, SLS, FFF, LENS, DIW	Ta Ti alloys Fe, Mg alloys PLA, PCL, PLGA HAp, β-TCP PCL/β-TCP, PLA/HAp	Strut-based BCC, FCC, diamond, octet-truss, TPMS gyroid, Voronoi	Biocompatibility, bioactivity, high interconnected porosity, high mechanical strength under load, osteointegration, anatomical adaptability	[14,17,22,24,28,32,34,35,36,41]
	Temporary bone substitutes	DLP, SLM, SLS, BJ, FFF, DIW	Ti alloys Fe, Mg, Zn alloys PLA, PCL, PLGA HAp, ZrO_2_, β-TCP PCL/β-TCP, PLA/HAp	Strut-based cubic, diamond, octet-truss, octahedron and TPMS gyroid	Biocompatibility, bioactivity, biodegradability, osteointegration, anatomical adaptability	[17,22,23,26,27,28,29,30,35,37,41]
	Osteochondral implants	DIW, 3DBP	GelMA PCL/β-TCP	Strut-based diamond, auxetic, TPMS gyroid Wall-based TPMS gyroid	Biocompatibility, high interconnected porosity, mechanical stability	[12,35,49]
Tissue engineering	Regeneration of articular cartilage	FFF, SLS, DIW, 3DBP	PCL GelMA, alginate, collagen, chitosan	Strut-based cubic, TPMS gyroid, diamond, Wall-based gyroid, diamond	Osteochondral integration, high interconnected porosity, biomimetic mechanics	[14,22,27,29,43,47,49]
	Skin regeneration	DIW, 3DBP	GelMA, chitosan, gelatin	2.5D Honeycomb Strut-based cubic Wall-based cubic	Biocompatibility, permeability, adjustable mechanical resistance, antimicrobial activity	[13,27,42]
Tissue engineering	Muscle regeneration	Electrospinning	GelMA, chitosan, gelatin PCL/spun fibers	Strut-based cubic	Bioelectrical compatibility, elasticity and mechanical adaptability	[14,29]
	Matrices for cell culture	DLP, FFF, MEW	HEMA PCL	2.5D Honeycomb, auxetic Strut-based cubic	Biocompatibility, high cell adhesion, high interconnected porosity, structural stability	[15,18,21,26,29,35,49]
	Matrices for tissue bio printing	FFF, DIW, 3DBP	PCL GelMA, alginate, collagen, chitosan Composite hydrogels	Strut-based diamond Wall-based cubic	Biocompatibility, biodegradability, imitation of the extracellular environment	[21,32,39,49]
	Matrices for direct printing of organoids	DLP, BJ, FFF, DIW, 3DBP	PCL GelMA, alginate, collagen	Strut-based cubic	Biocompatibility, bioactivity, high interconnected porosity, permeability	[21,29,32]
Implantable devices	Devices for controlled drug release	DLP, FFF, SLM, BJ, DIW	Ni alloys PLA, PCL, PLGA Alginate, collagen, chitosan	Strut-based cubic, TPMS gyroid Wall-based TPMS gyroid, Schwarz	Biodegradability, high interconnected porosity, controlled or sequential release	[16,20,22,27,28,30,45]
	Sensory devices	4DP	MXenes	Strut-base cubic	Biocompatibility, biodegradability, high interconnected porosity, structural stability	[19]
	Temporary stents	SLM	Fe, Mg, Zn alloys	Strut-based BCC, diamond, TPMS gyroid, diamond	Biocompatibility, biodegradability, anatomical adaptability	[26,30,33]

## Data Availability

No new data were created or analyzed in this study.
